# Supplementary figures and images for: Assay of cardiopulmonary bypass system for porcine alveolar macrophages removing GFP-E. coli from erythrocyte surfaces
Source: PeerJ. 2025 Mar 4;13:e18934. doi: 10.7717/peerj.18934 (PMC11887565; doi:10.7717/peerj.18934)

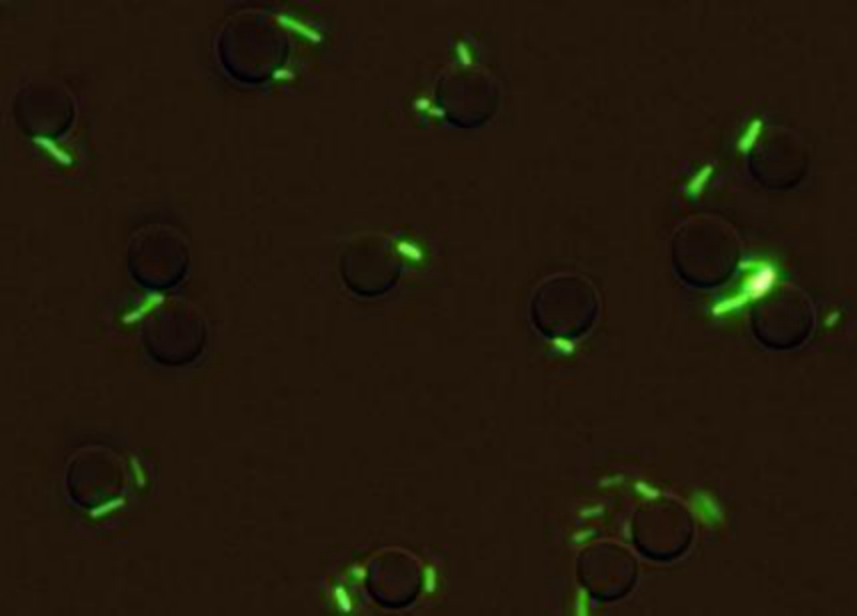

Supplement: Supplemental Information 2 [file peerj-13-18934-s002.zip › Picture supplement/Figure 1/Figure 1.pdf]

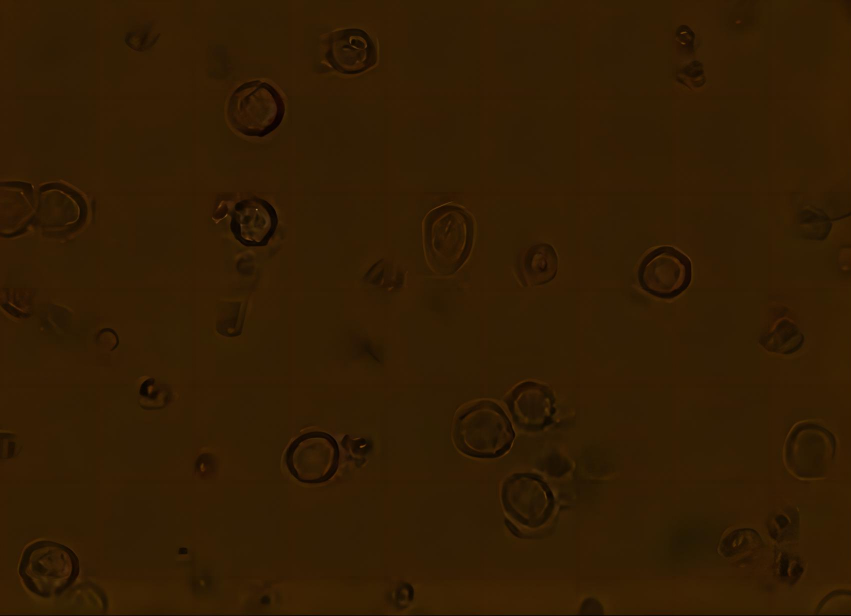

Supplement: Supplemental Information 2 [file peerj-13-18934-s002.zip › Picture supplement/Figure 1/Figure 1A-2.pdf]

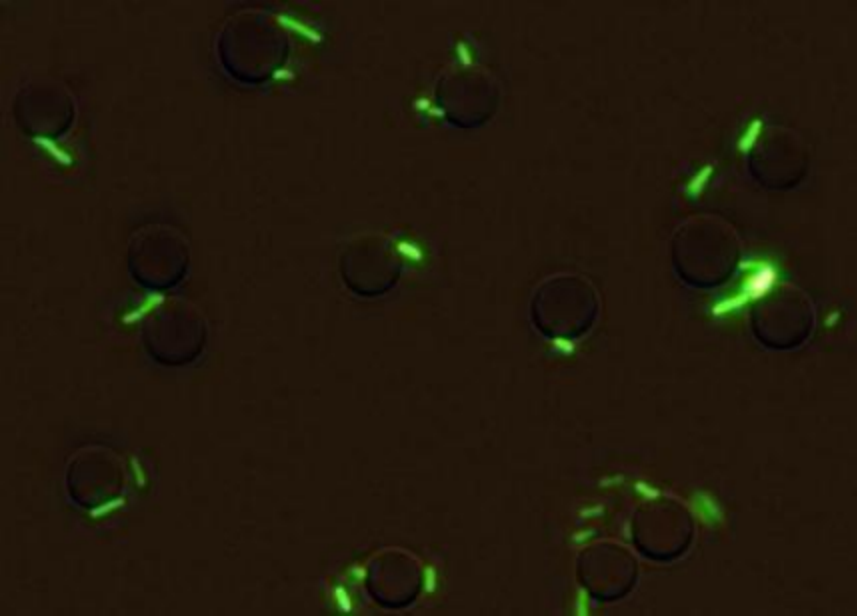

Supplement: Supplemental Information 2 [file peerj-13-18934-s002.zip › Picture supplement/Figure 1/Figure 1B-1.pdf]

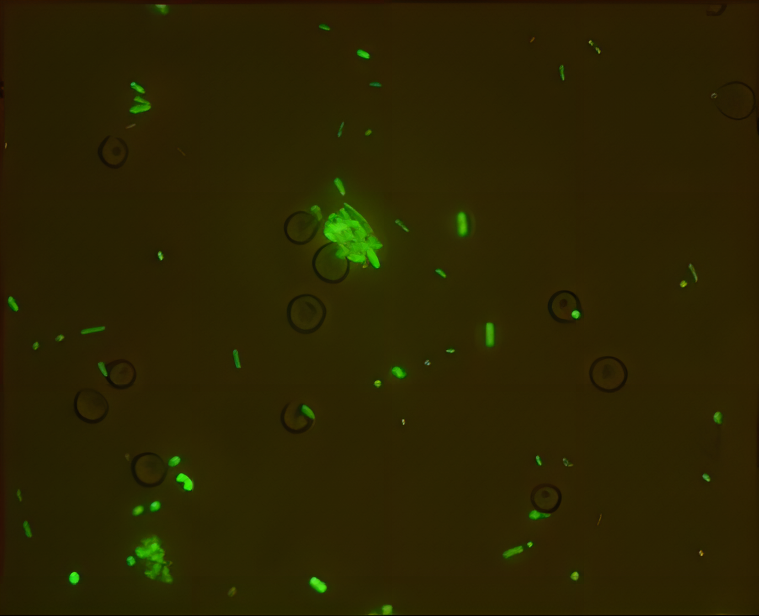

Supplement: Supplemental Information 2 [file peerj-13-18934-s002.zip › Picture supplement/Figure 1/Figure 1B-2.pdf]

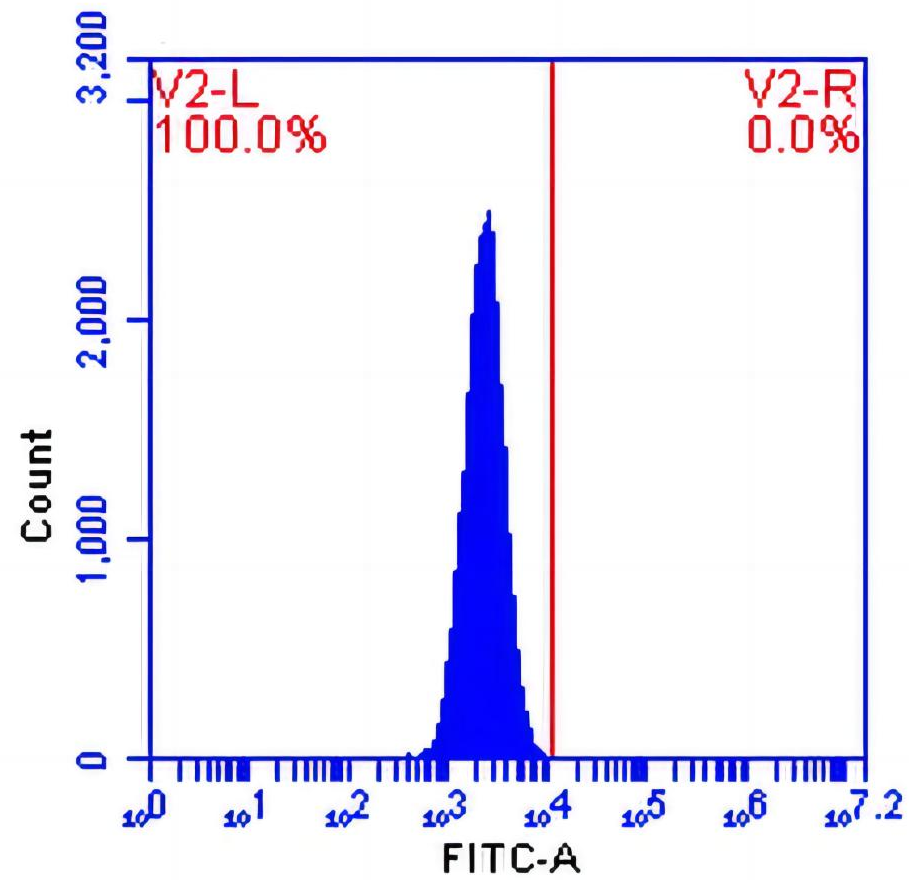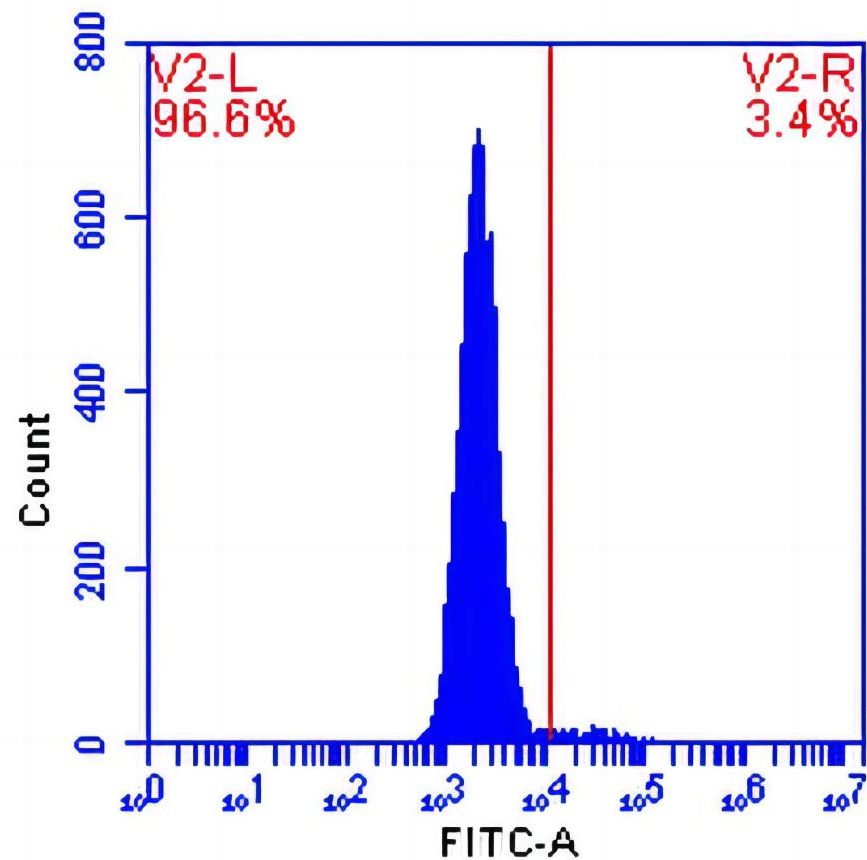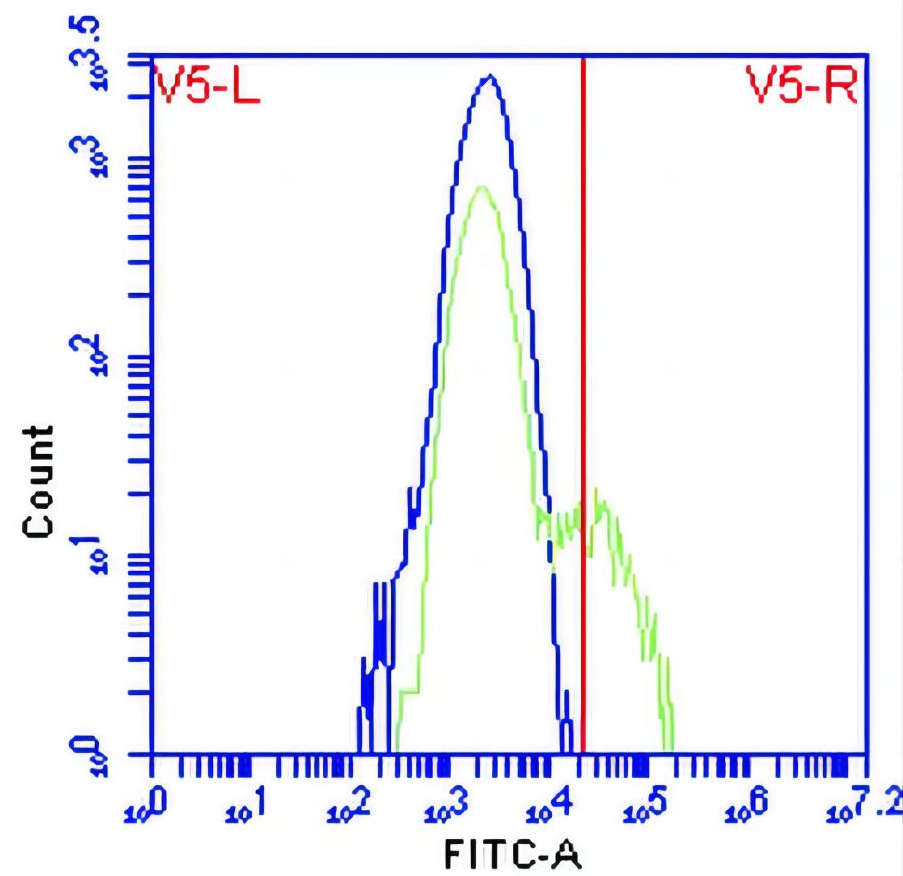

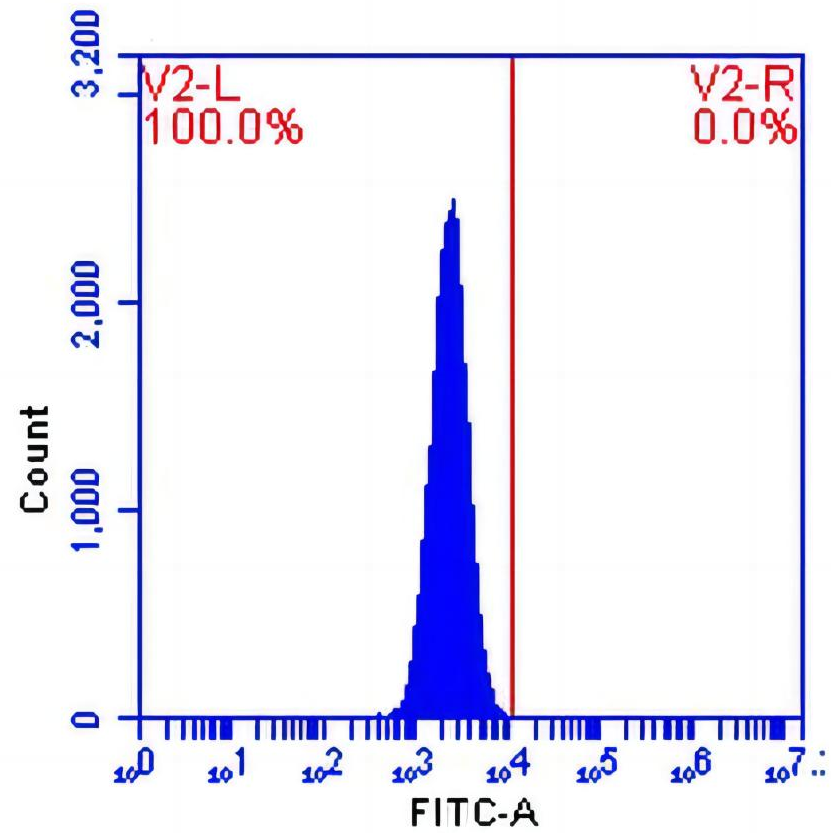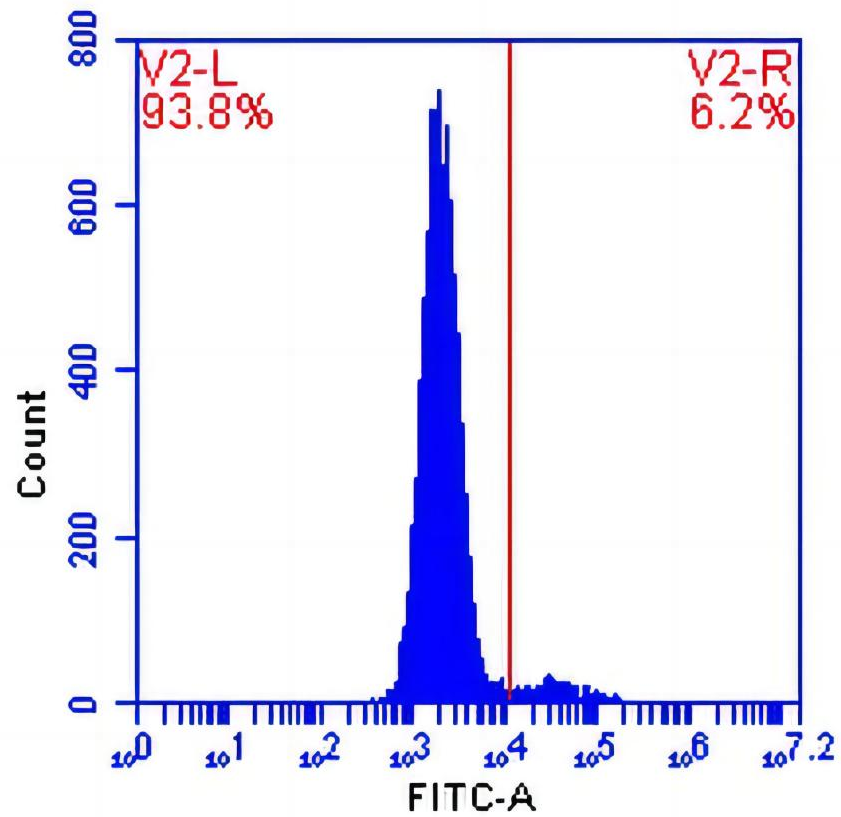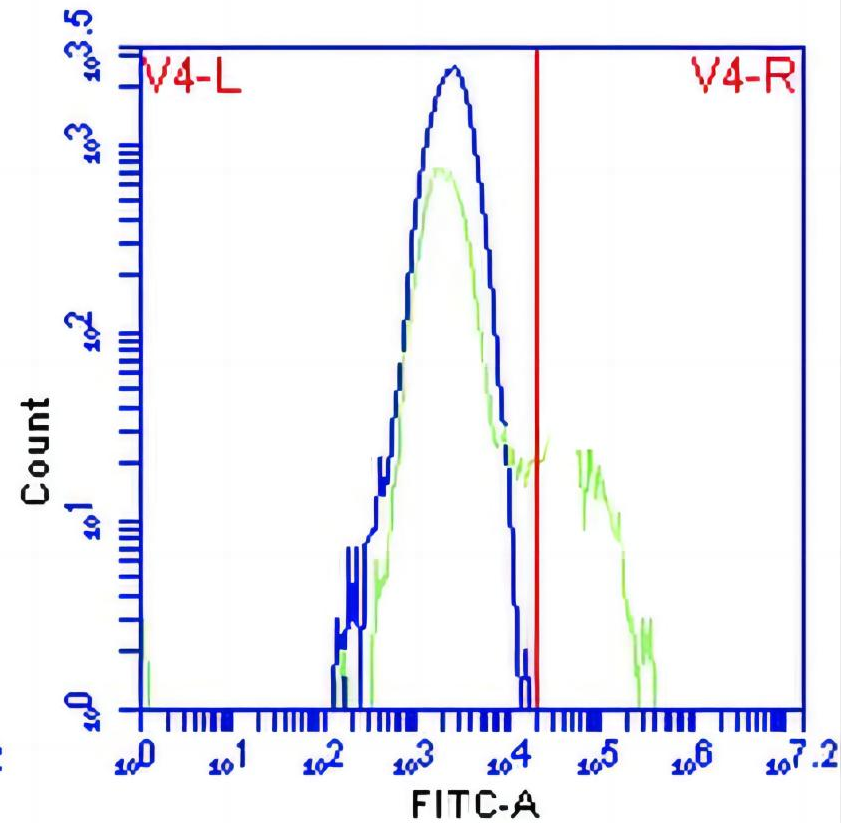

Supplement: Supplemental Information 2 [file peerj-13-18934-s002.zip › Picture supplement/Figure 10/Figure 10.pdf]

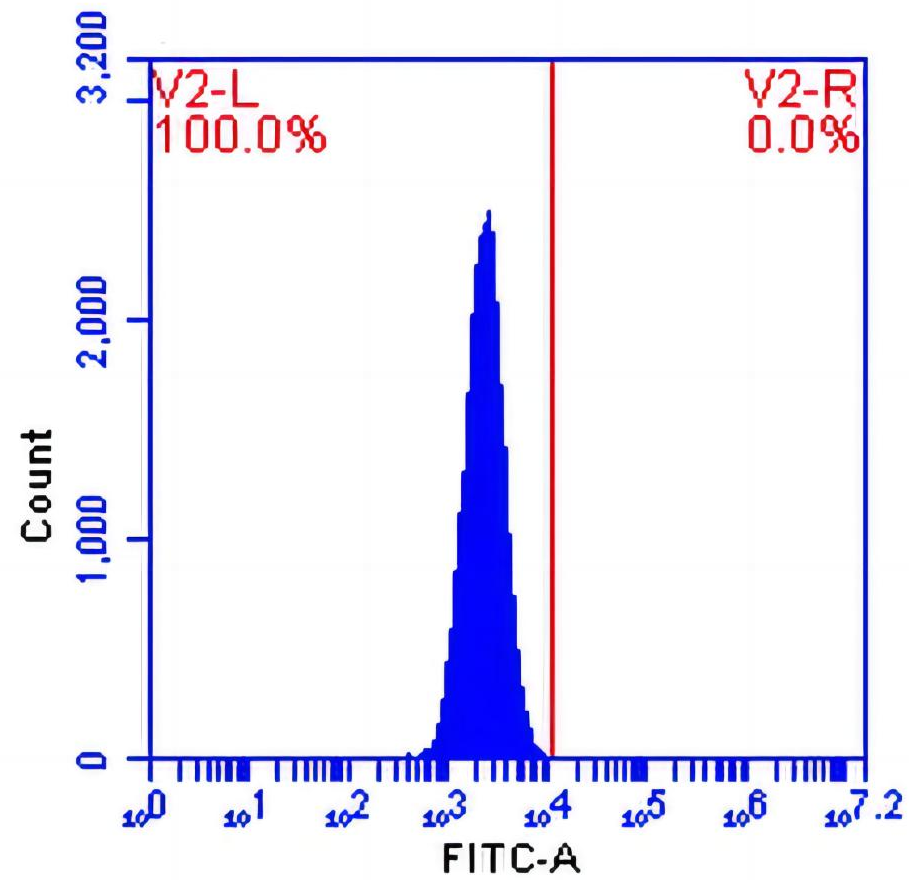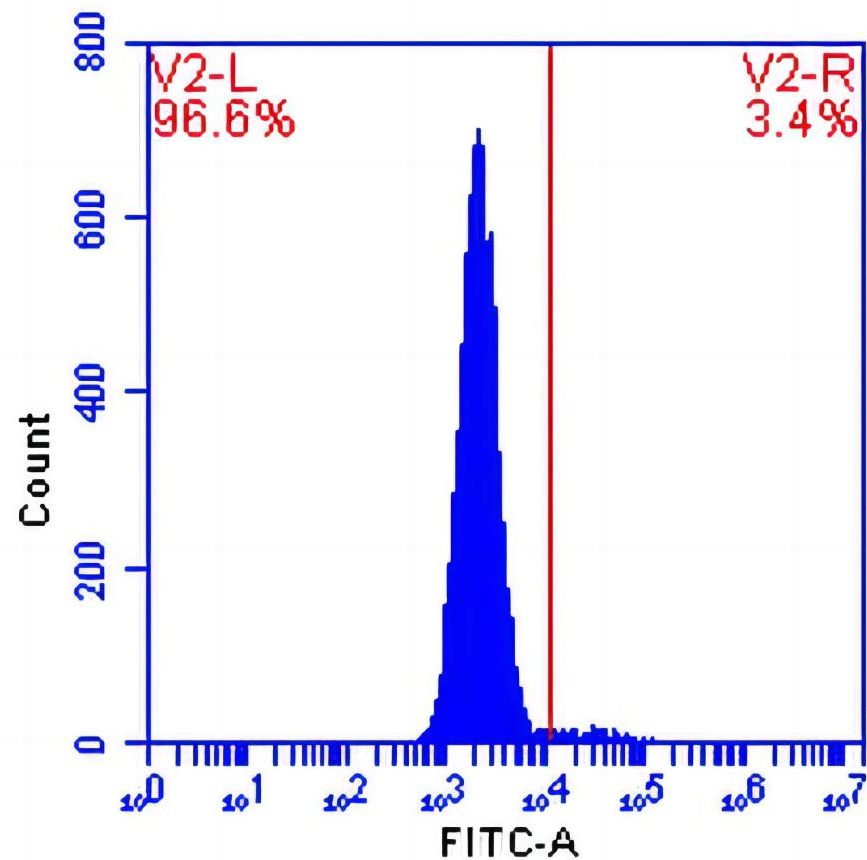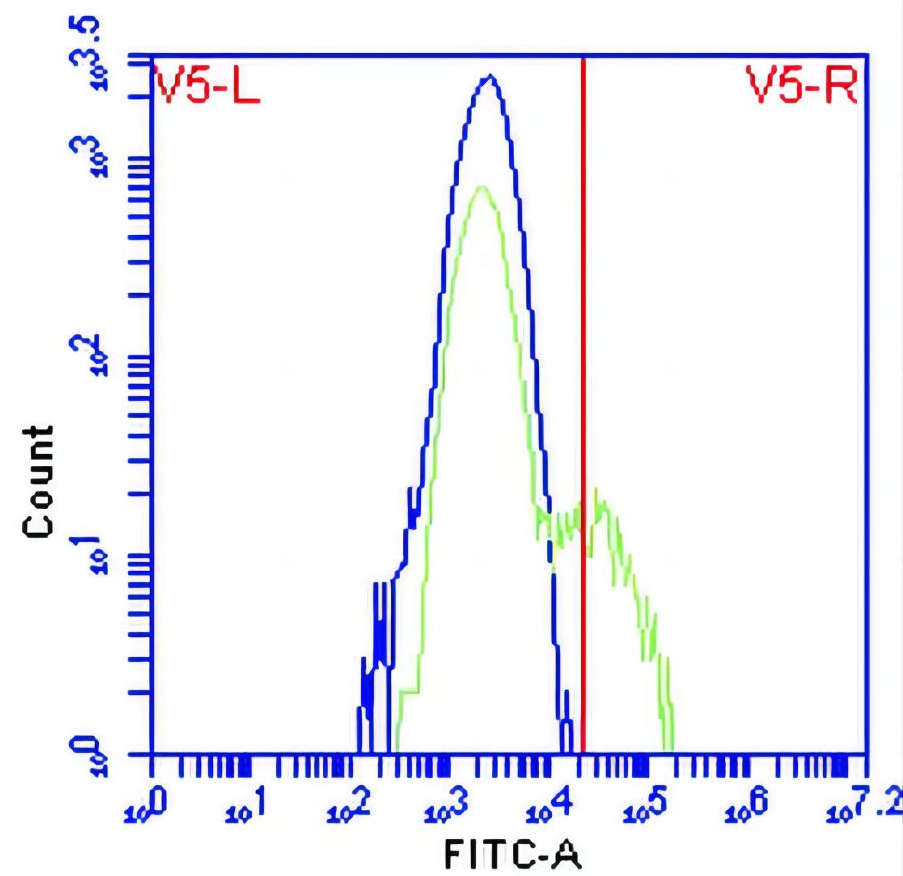

Supplement: Supplemental Information 2 [file peerj-13-18934-s002.zip › Picture supplement/Figure 10/Figure 10A.pdf]

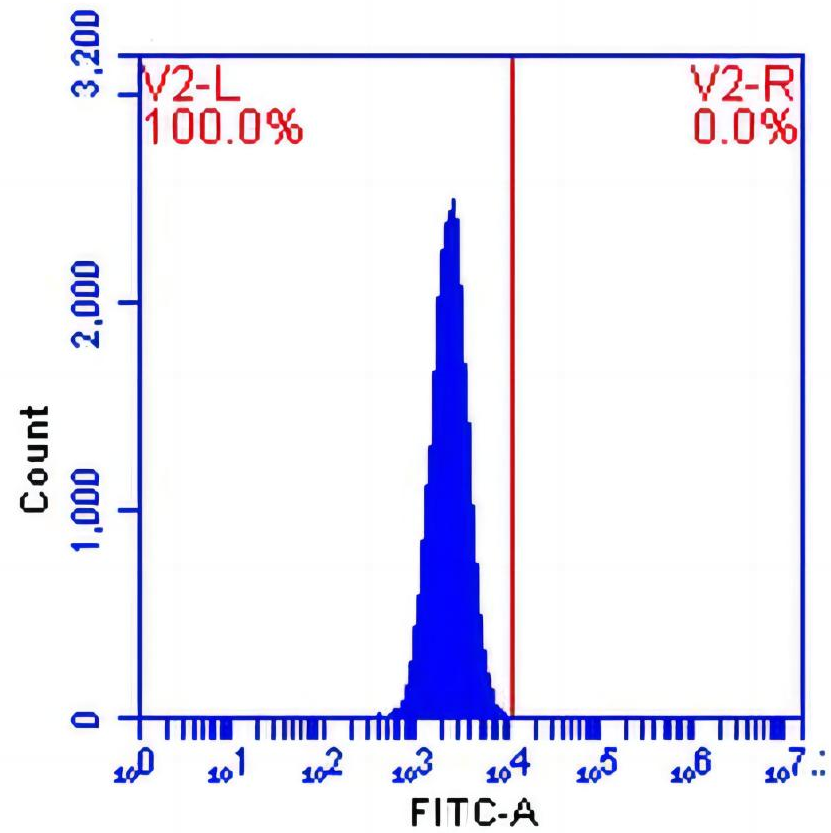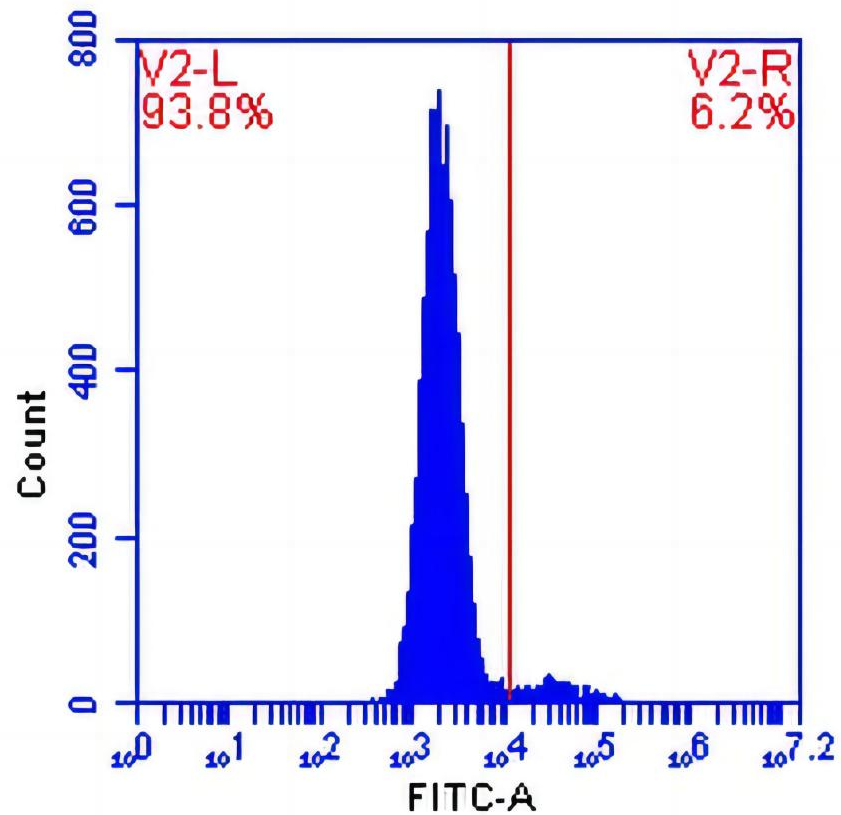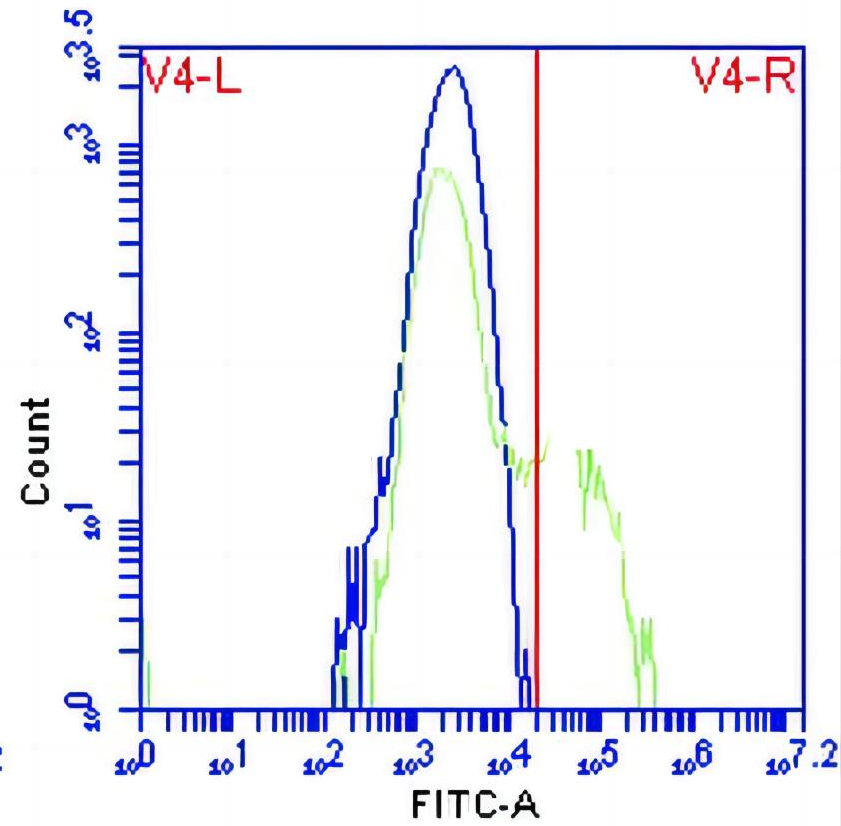

Supplement: Supplemental Information 2 [file peerj-13-18934-s002.zip › Picture supplement/Figure 10/Figure 10B.pdf]

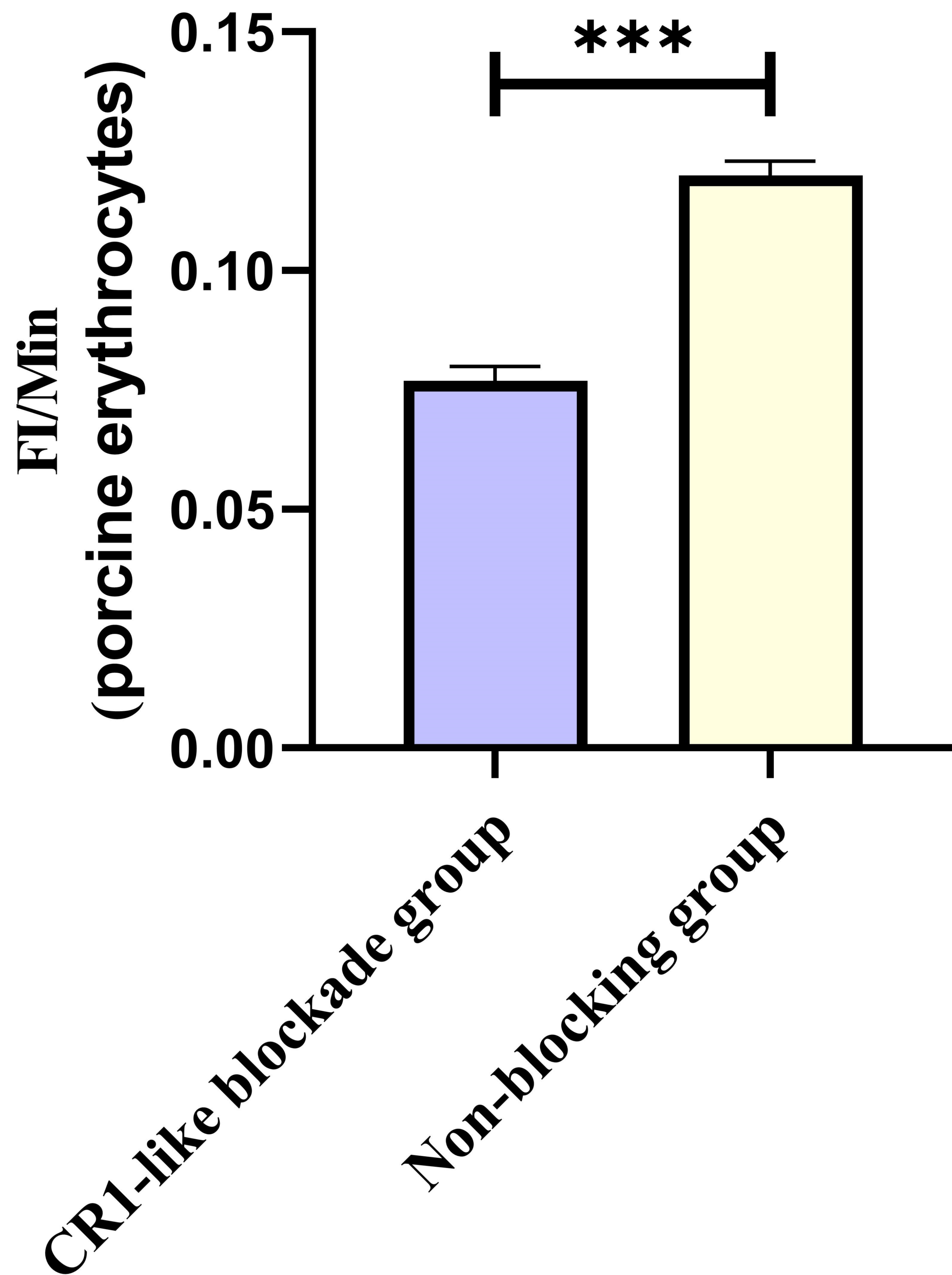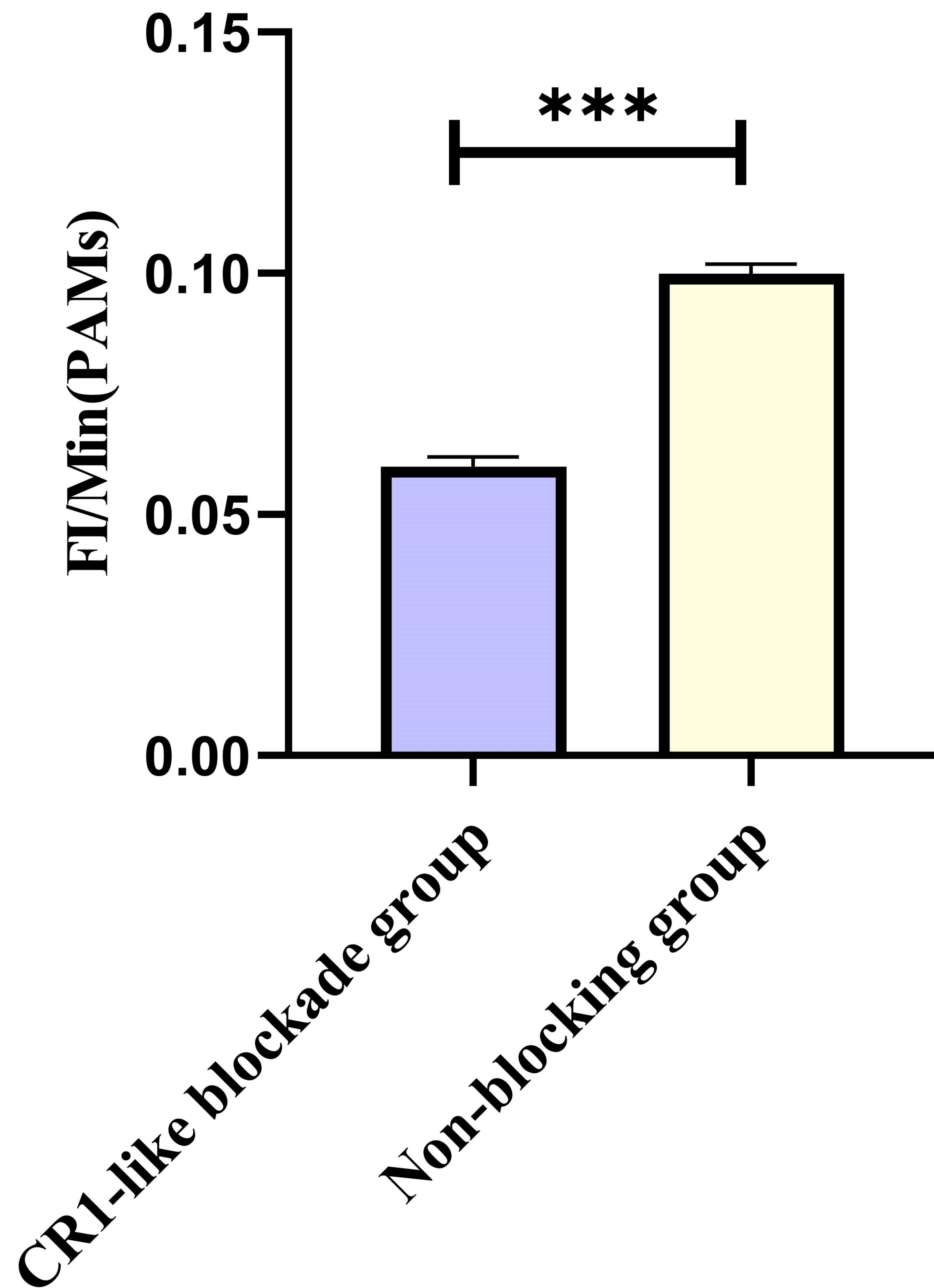

Supplement: Supplemental Information 2 [file peerj-13-18934-s002.zip › Picture supplement/Figure 11/Figure 11.pdf]

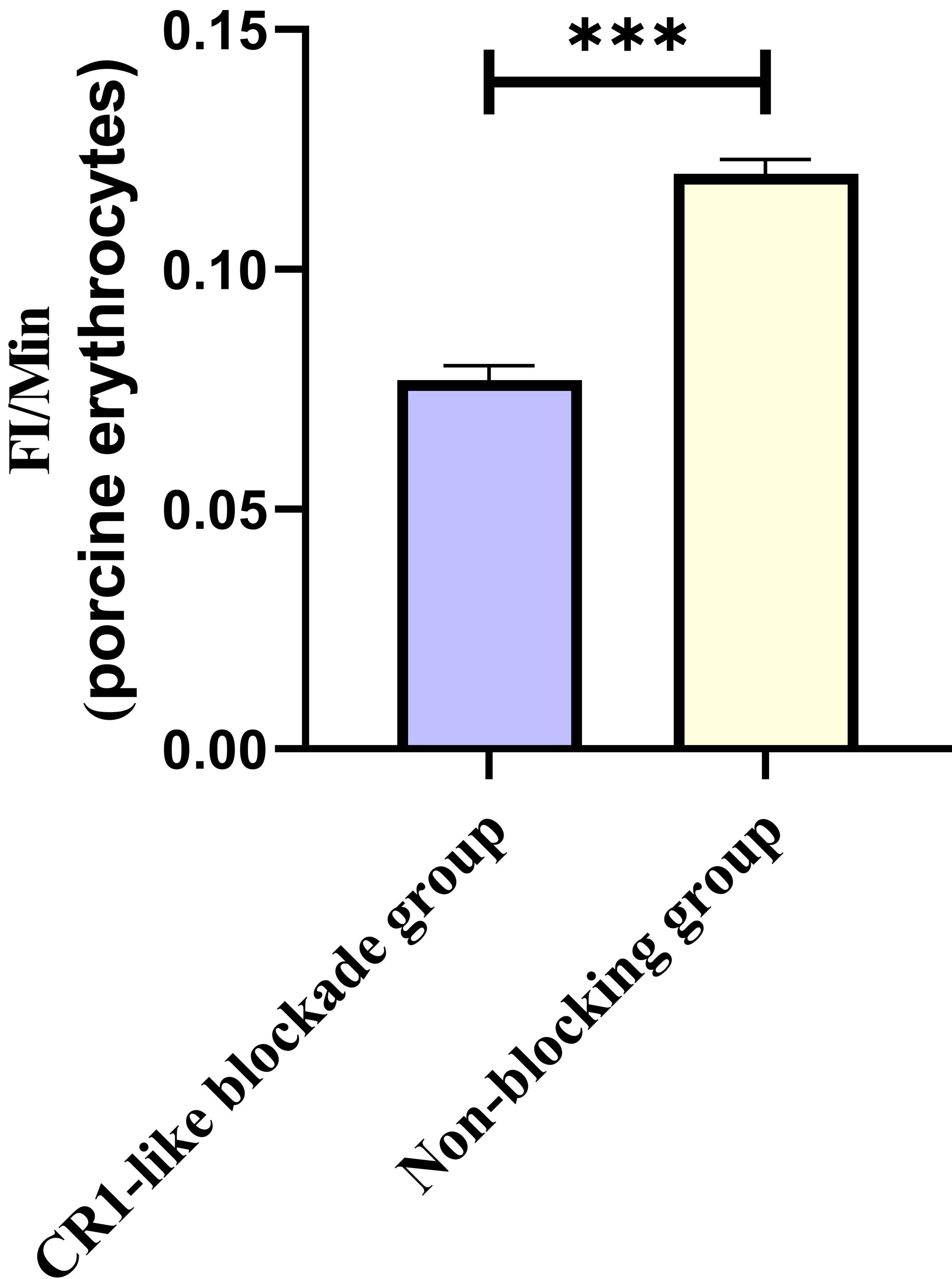

Supplement: Supplemental Information 2 [file peerj-13-18934-s002.zip › Picture supplement/Figure 11/Figure 11A.pdf]

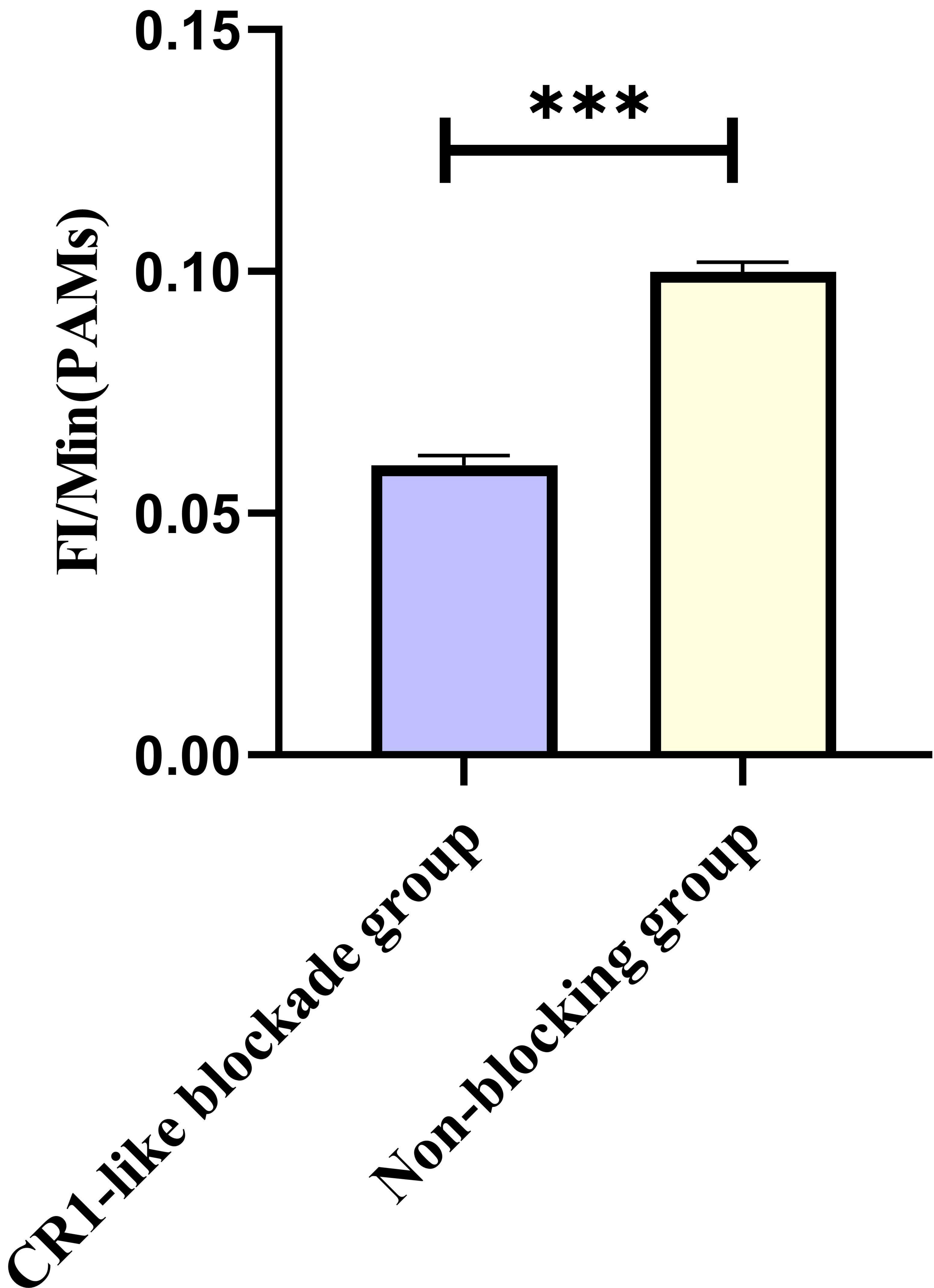

Supplement: Supplemental Information 2 [file peerj-13-18934-s002.zip › Picture supplement/Figure 11/Figure 11B.pdf]

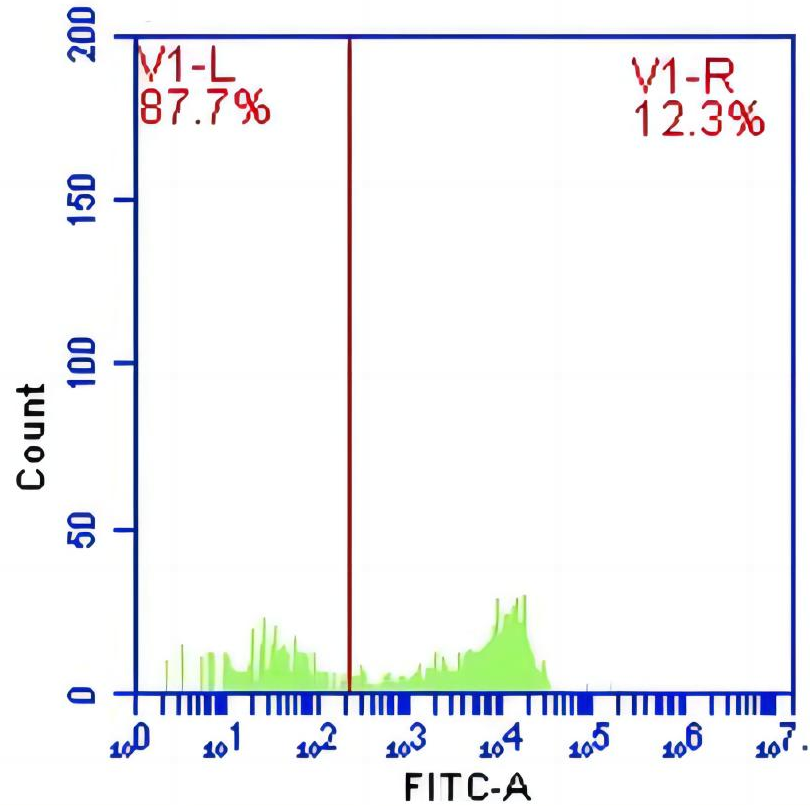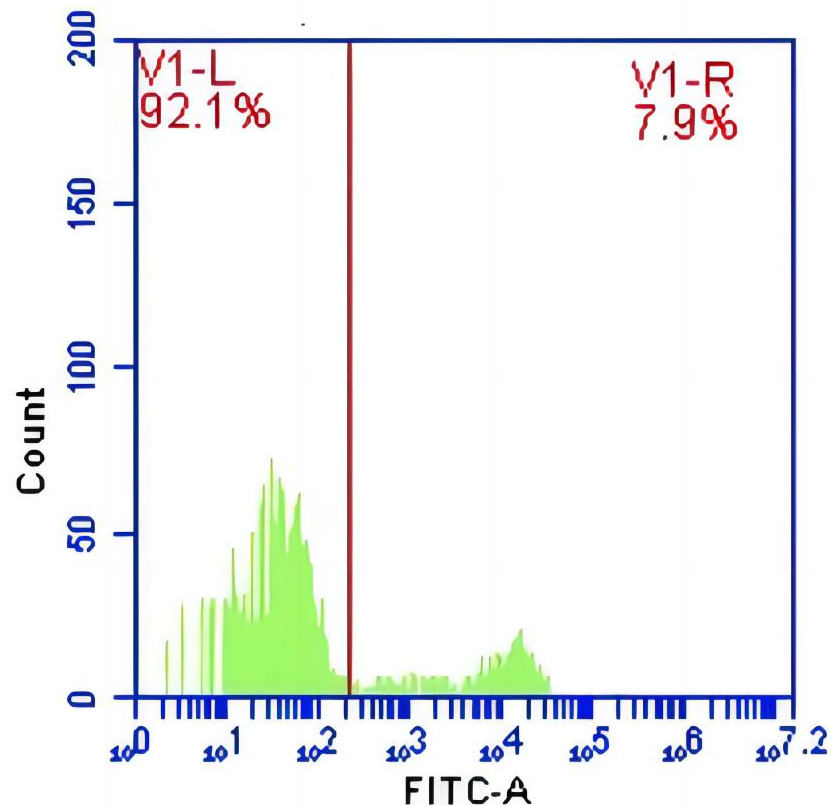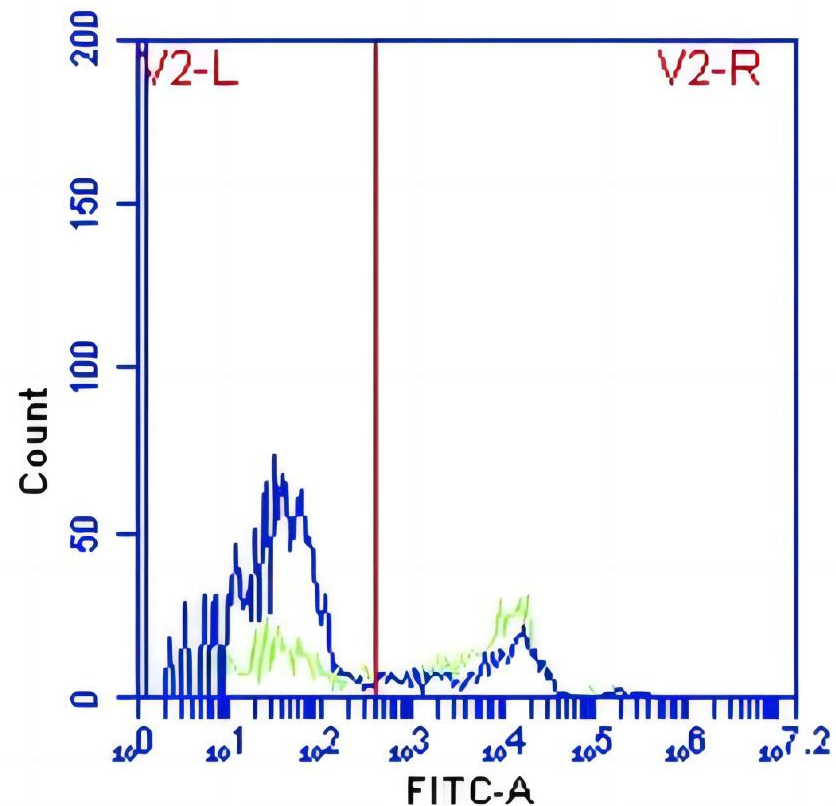

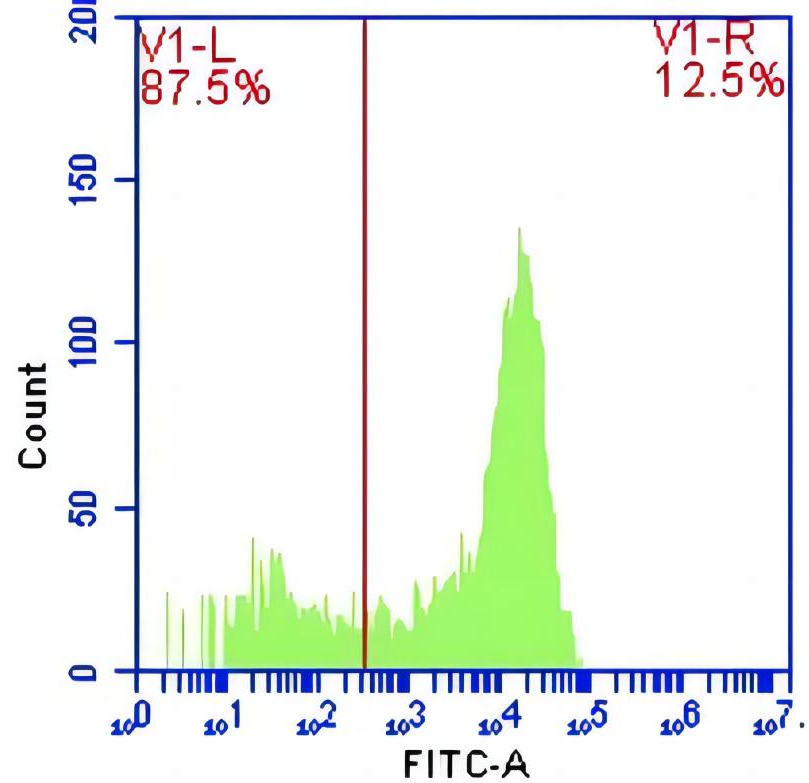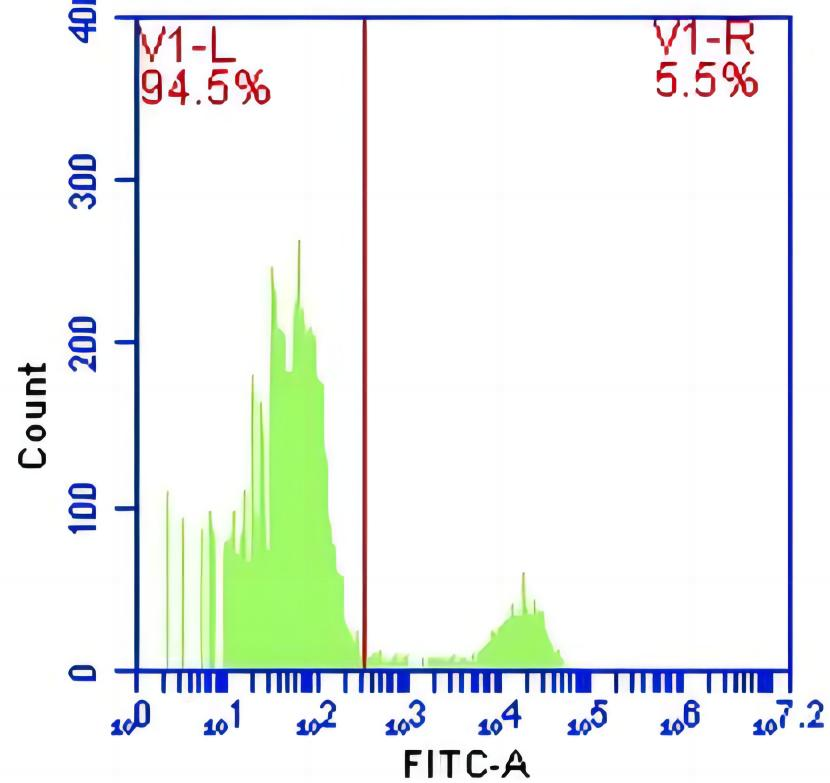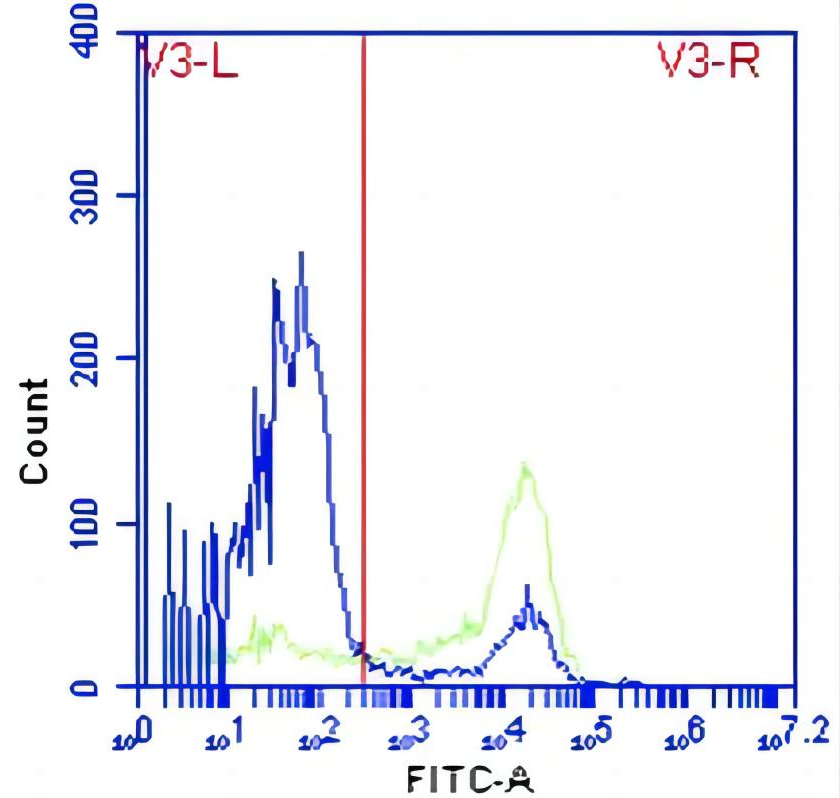

Supplement: Supplemental Information 2 [file peerj-13-18934-s002.zip › Picture supplement/Figure 12/Figure 12.pdf]

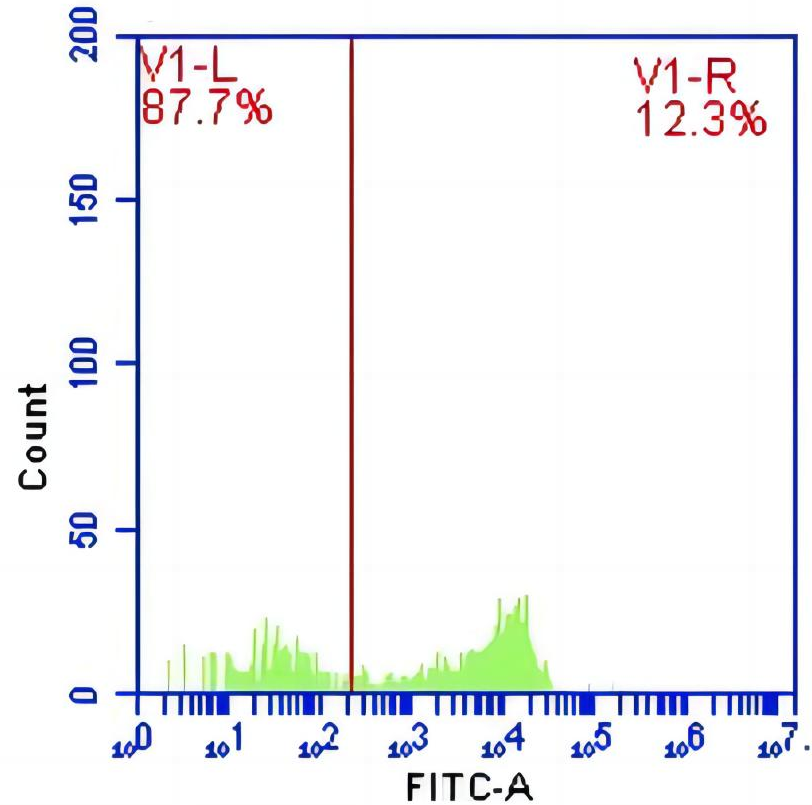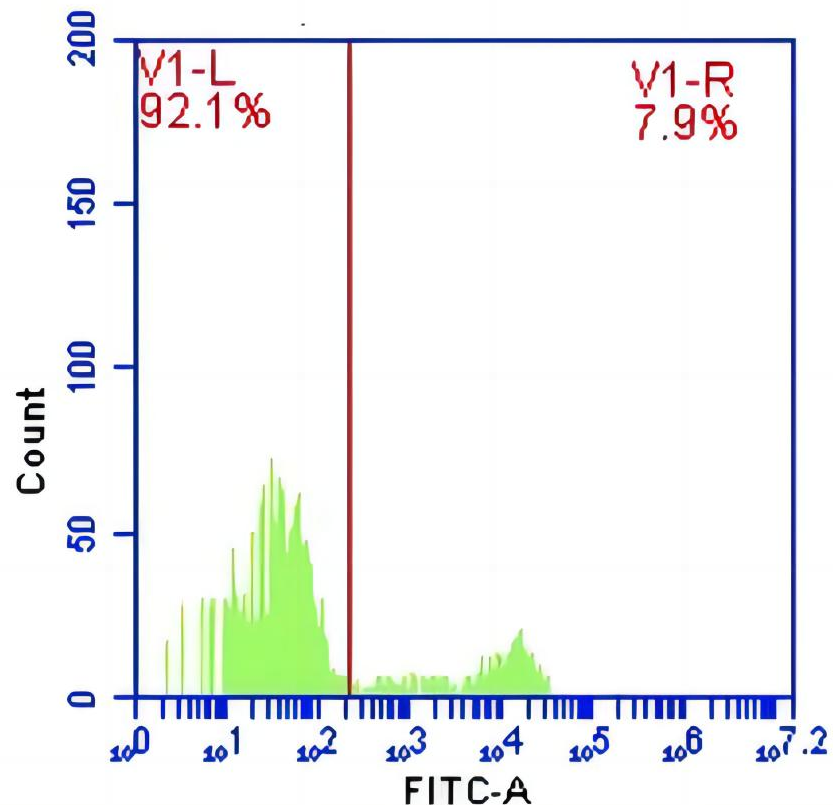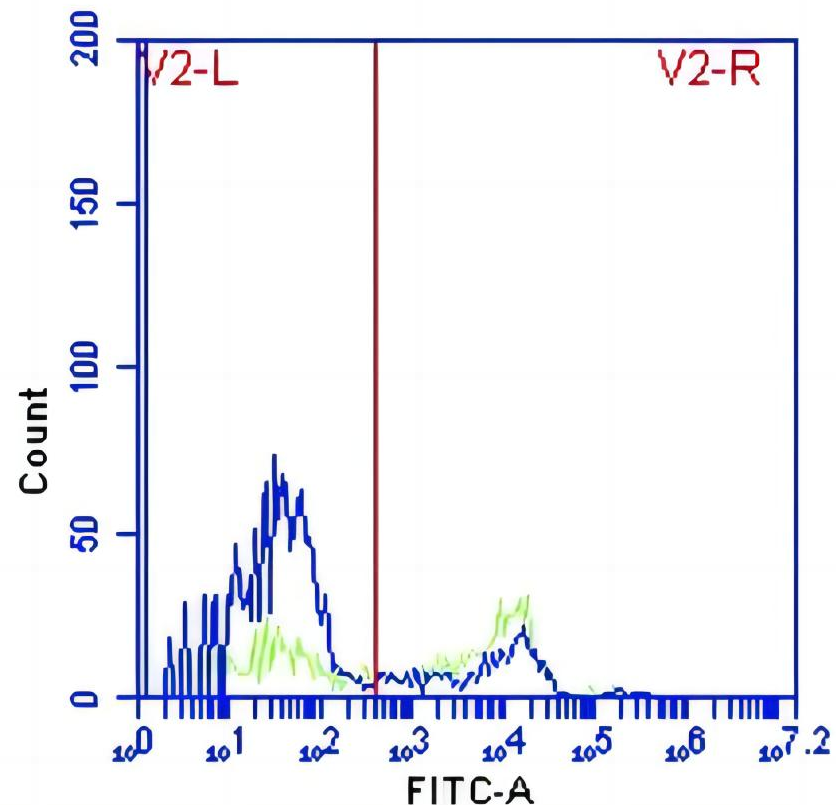

Supplement: Supplemental Information 2 [file peerj-13-18934-s002.zip › Picture supplement/Figure 12/Figure 12A.pdf]

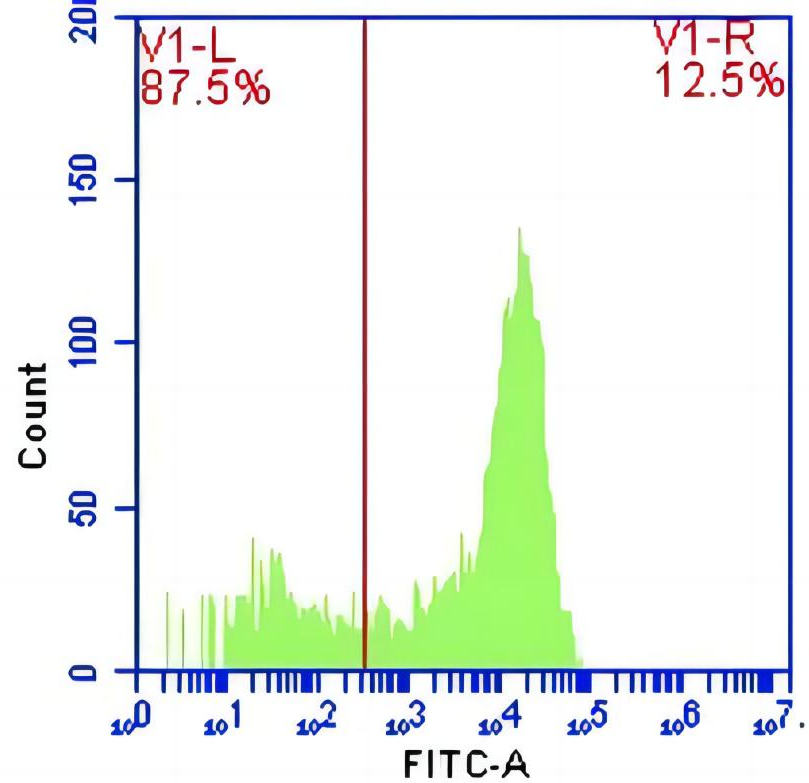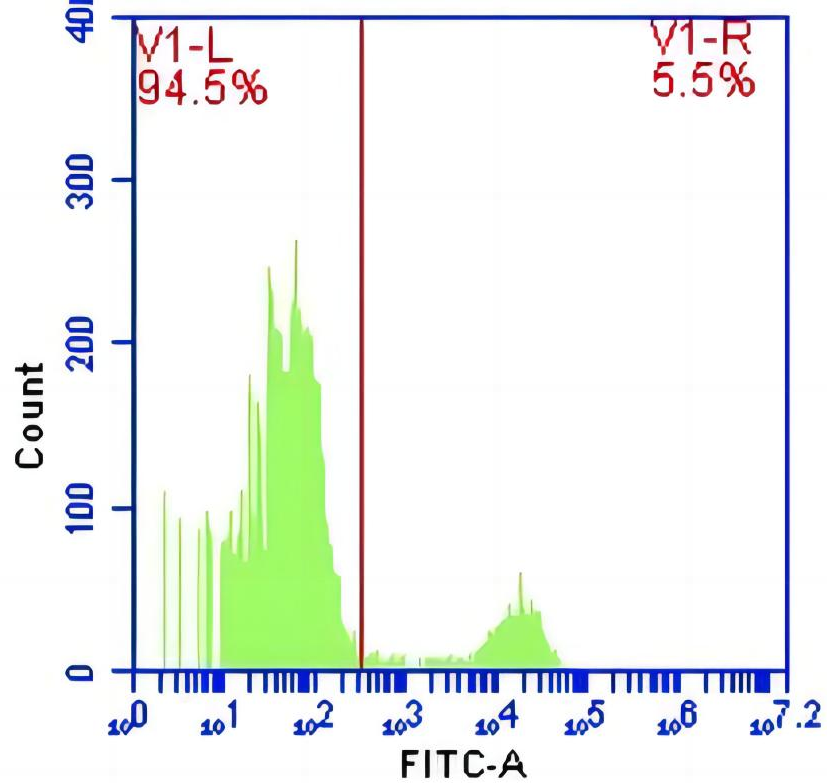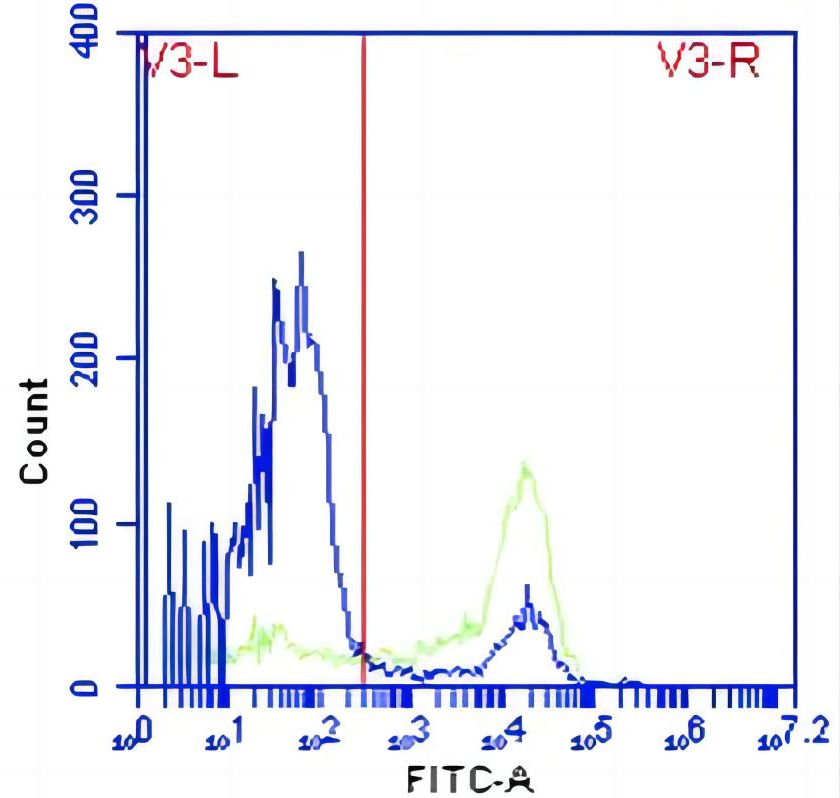

Supplement: Supplemental Information 2 [file peerj-13-18934-s002.zip › Picture supplement/Figure 12/Figure 12B.pdf]

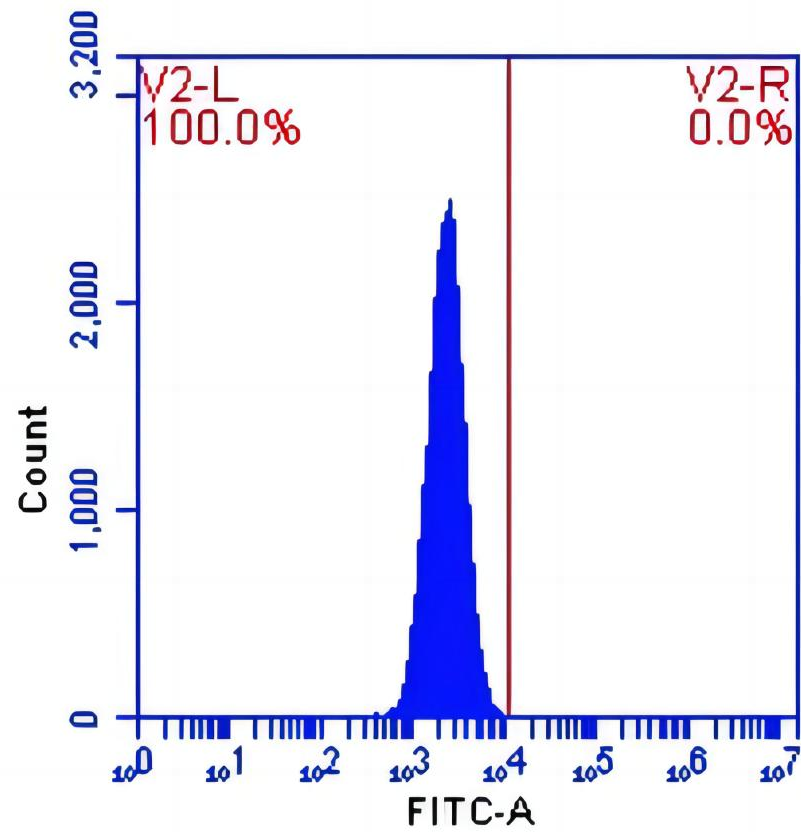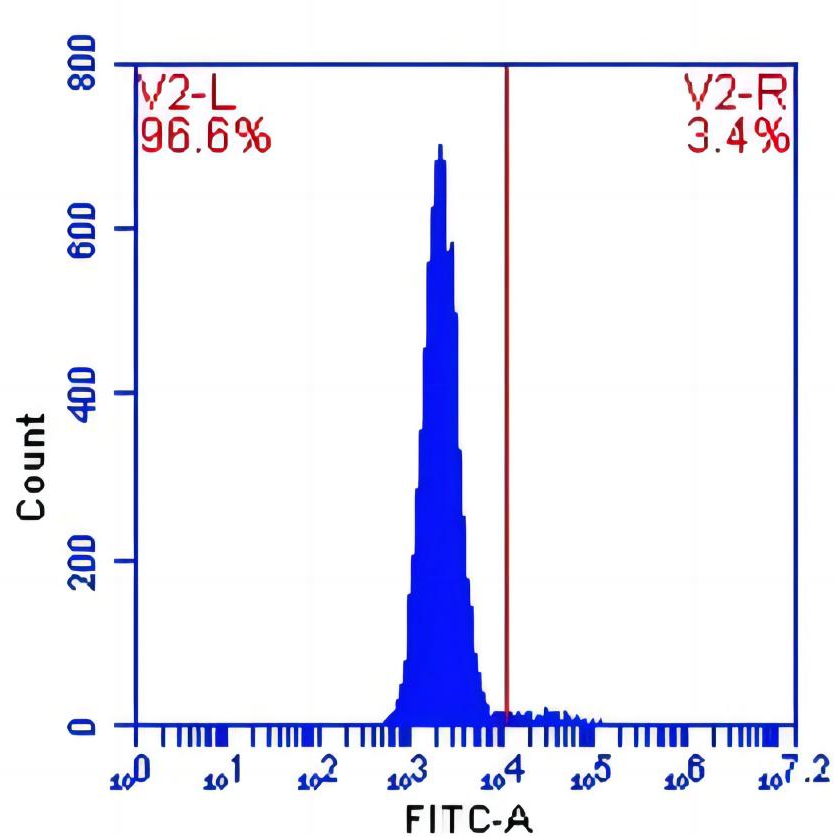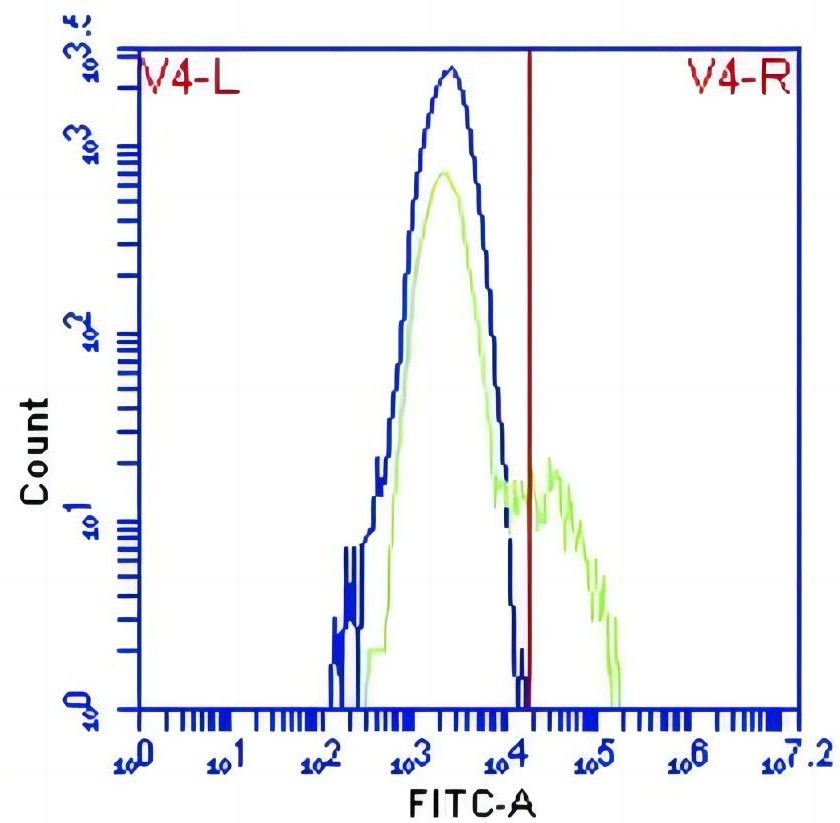

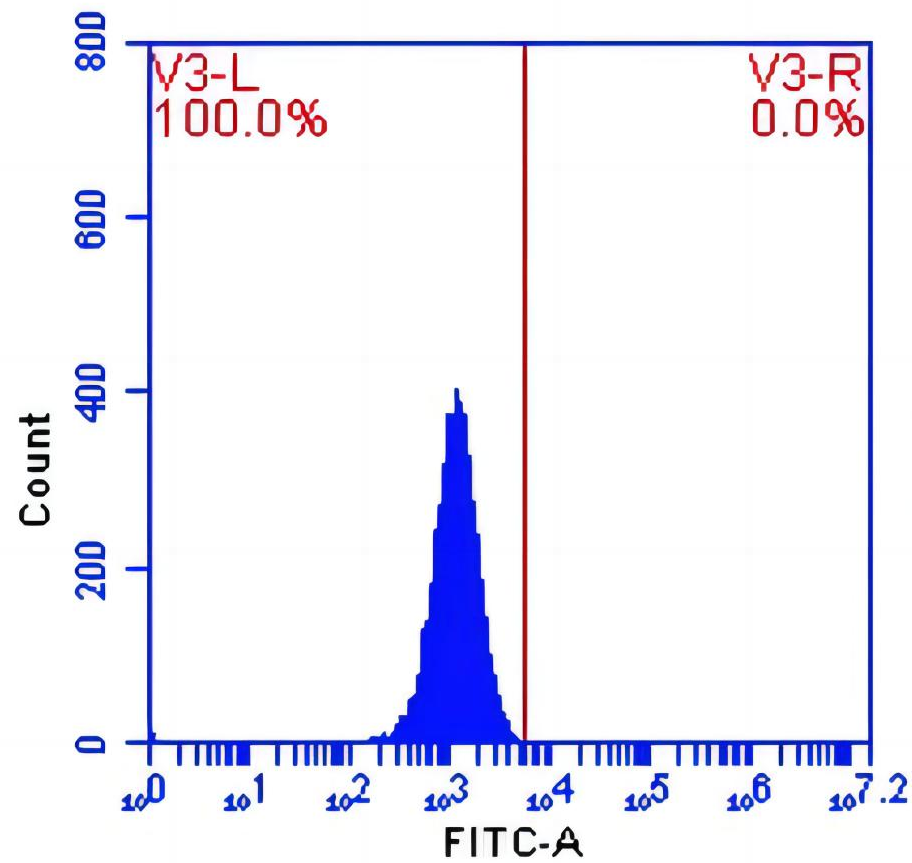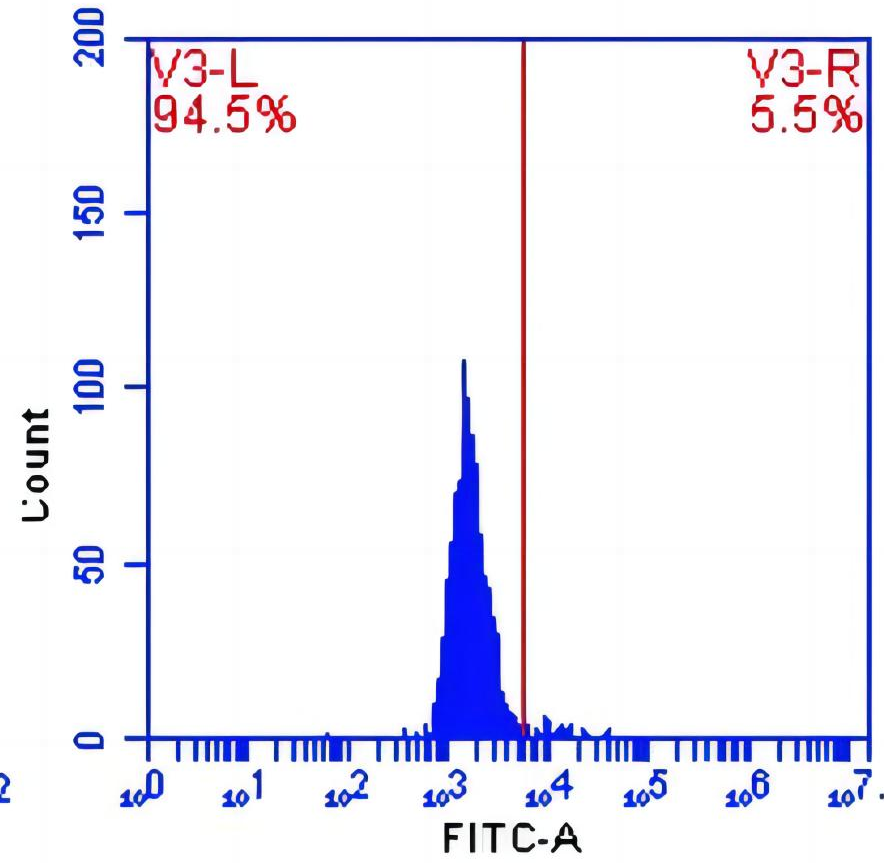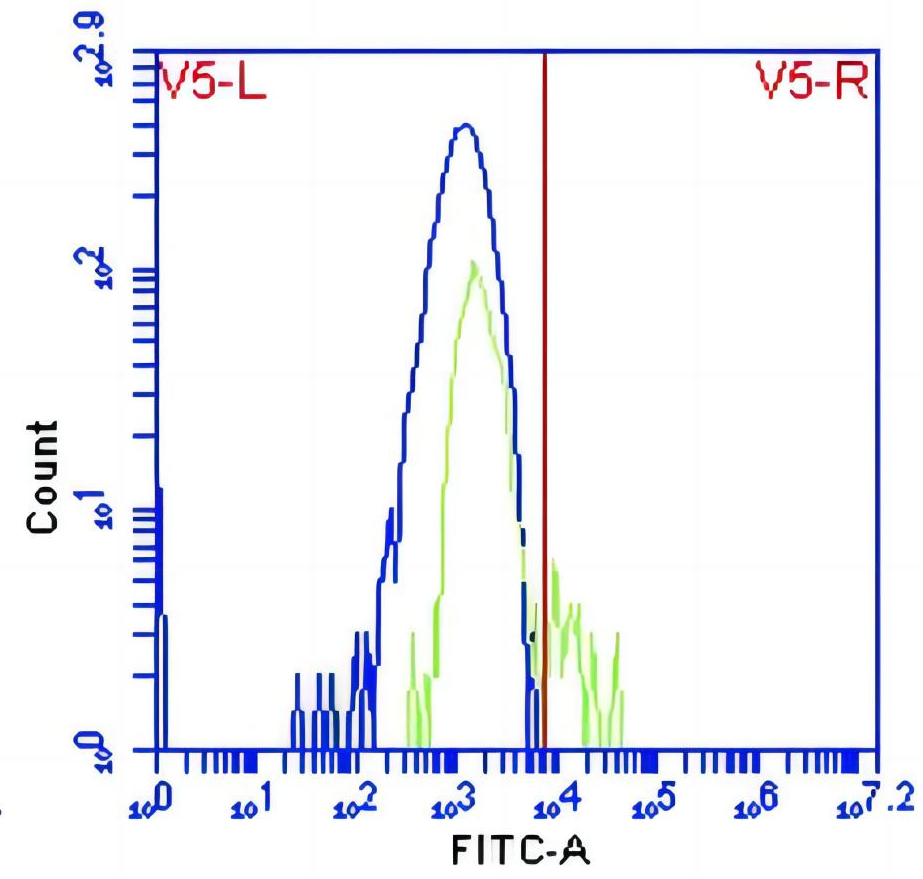

Supplement: Supplemental Information 2 [file peerj-13-18934-s002.zip › Picture supplement/Figure 13/Figure 13.pdf]

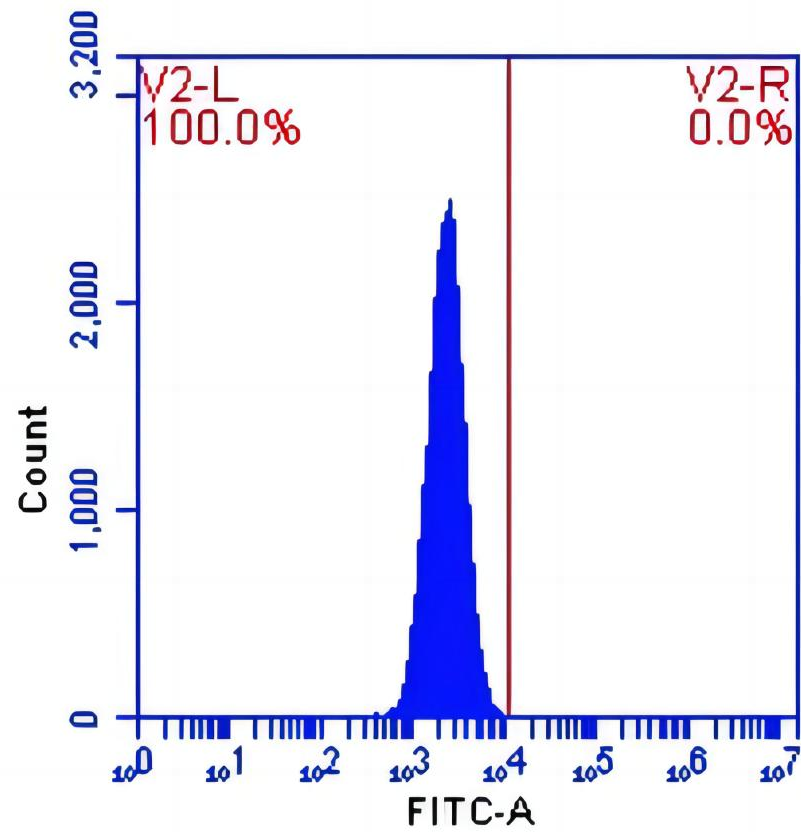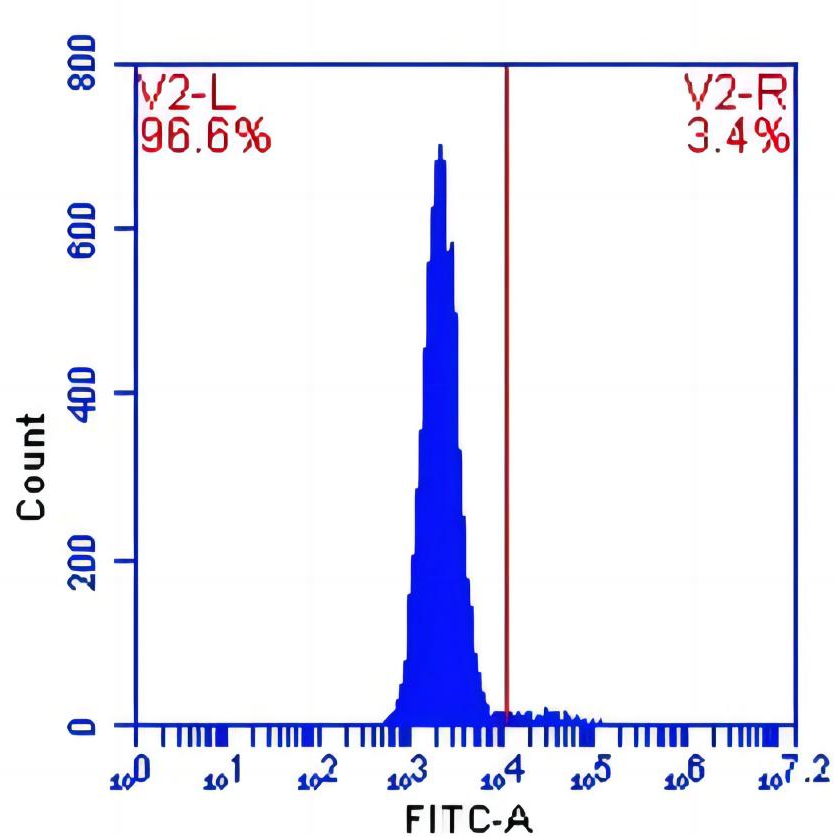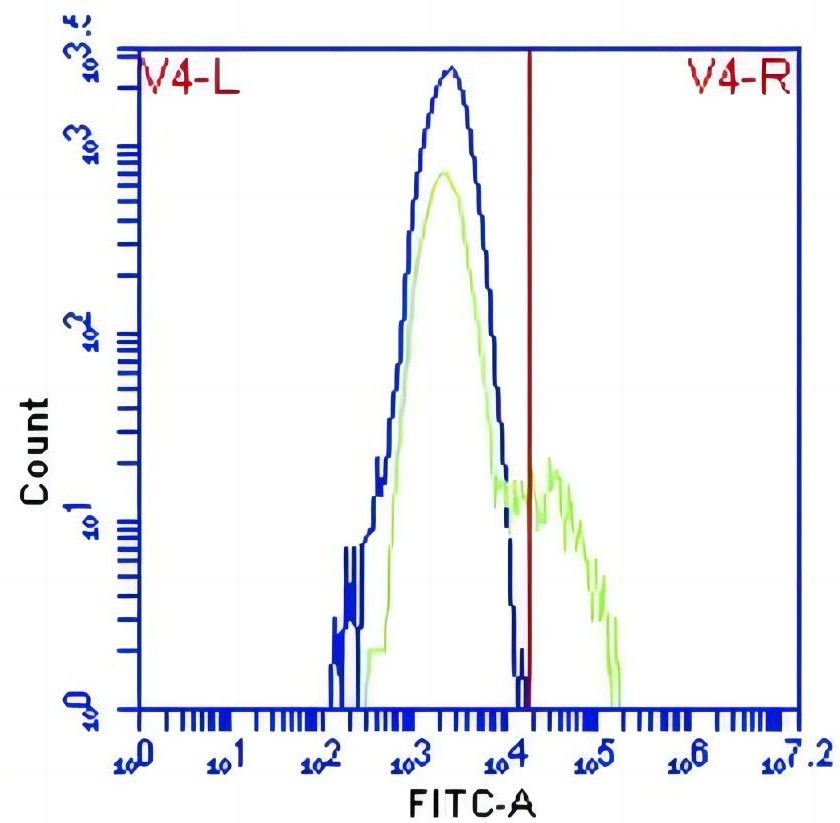

Supplement: Supplemental Information 2 [file peerj-13-18934-s002.zip › Picture supplement/Figure 13/Figure 13A .pdf]

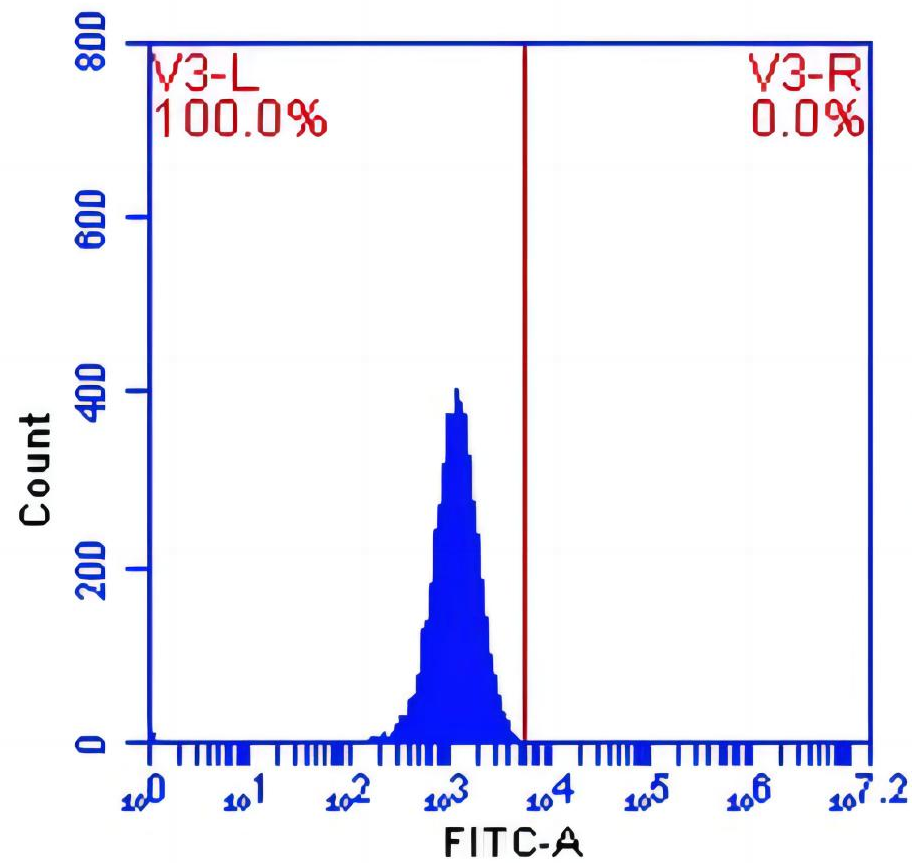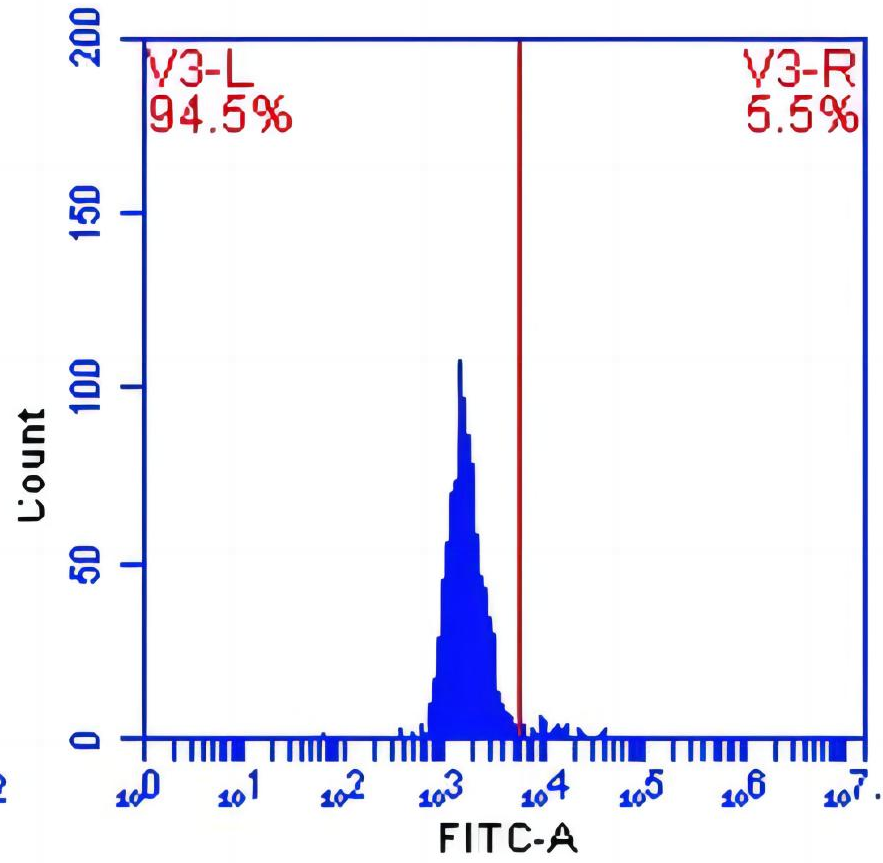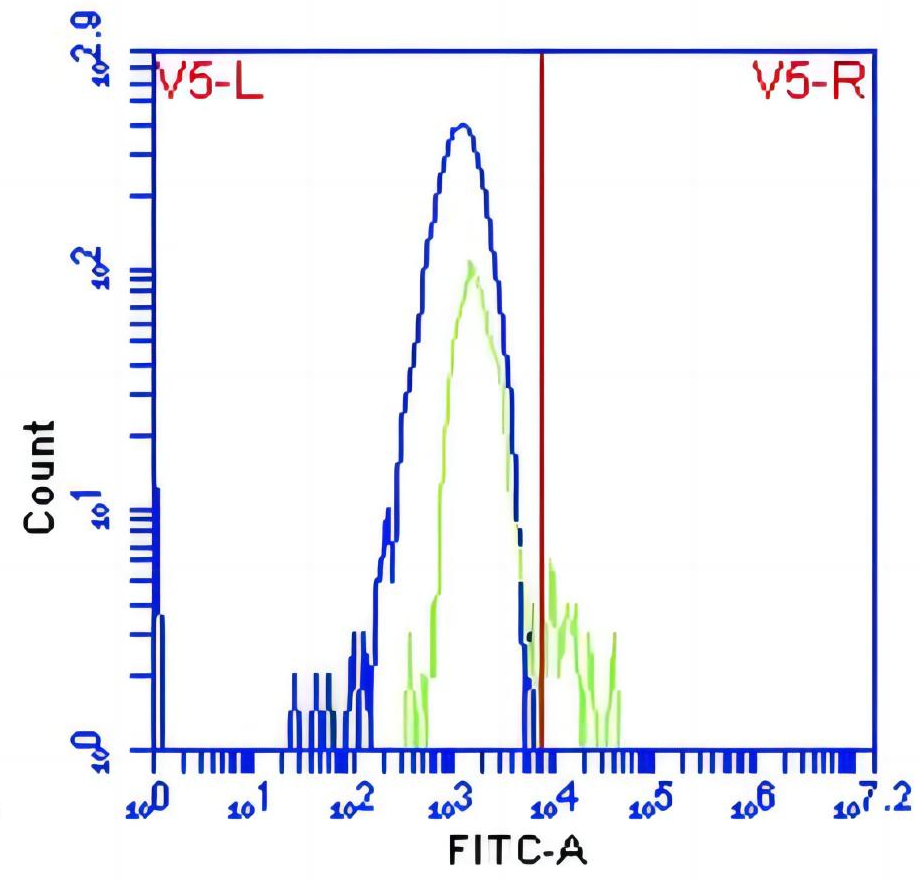

Supplement: Supplemental Information 2 [file peerj-13-18934-s002.zip › Picture supplement/Figure 13/Figure 13B.pdf]

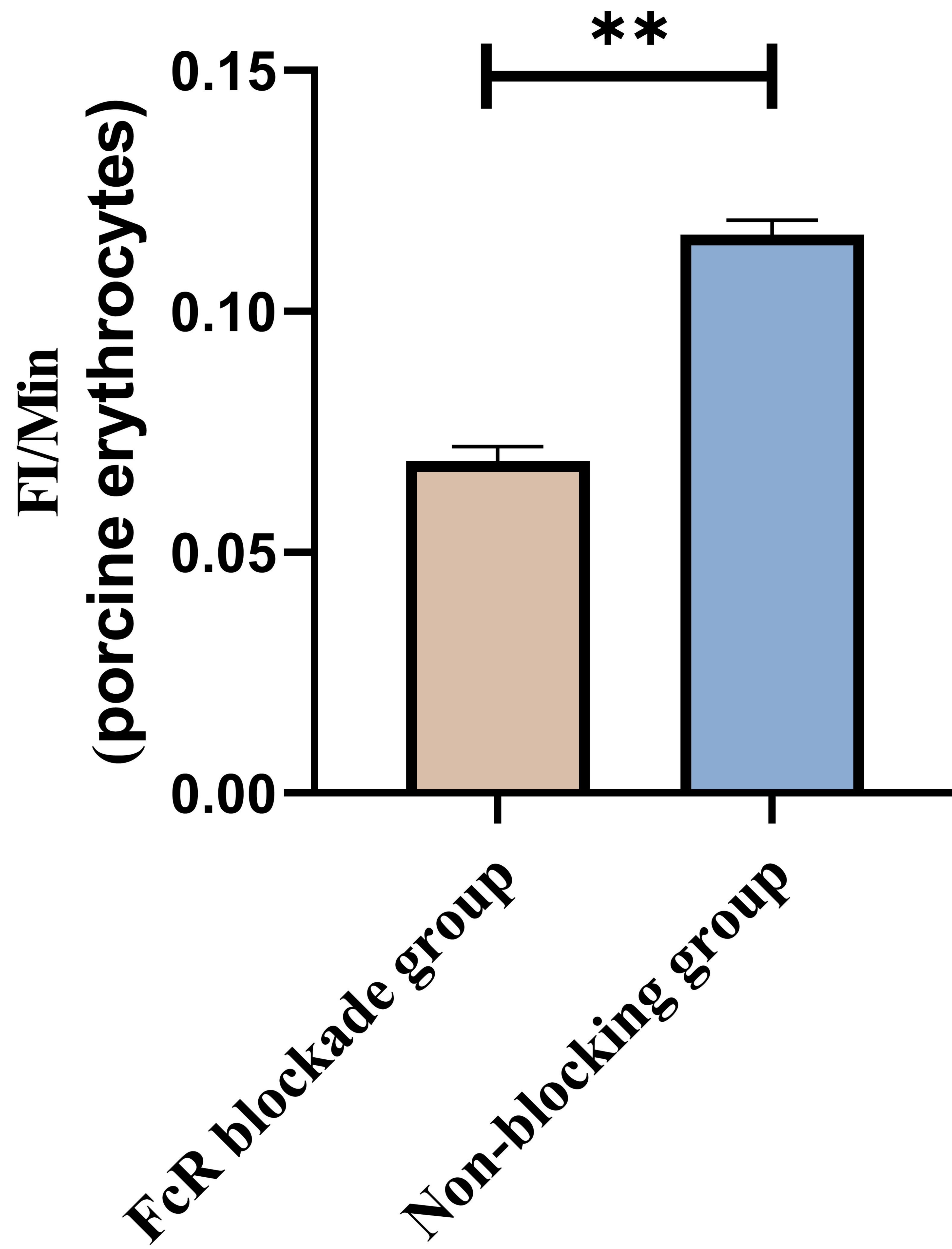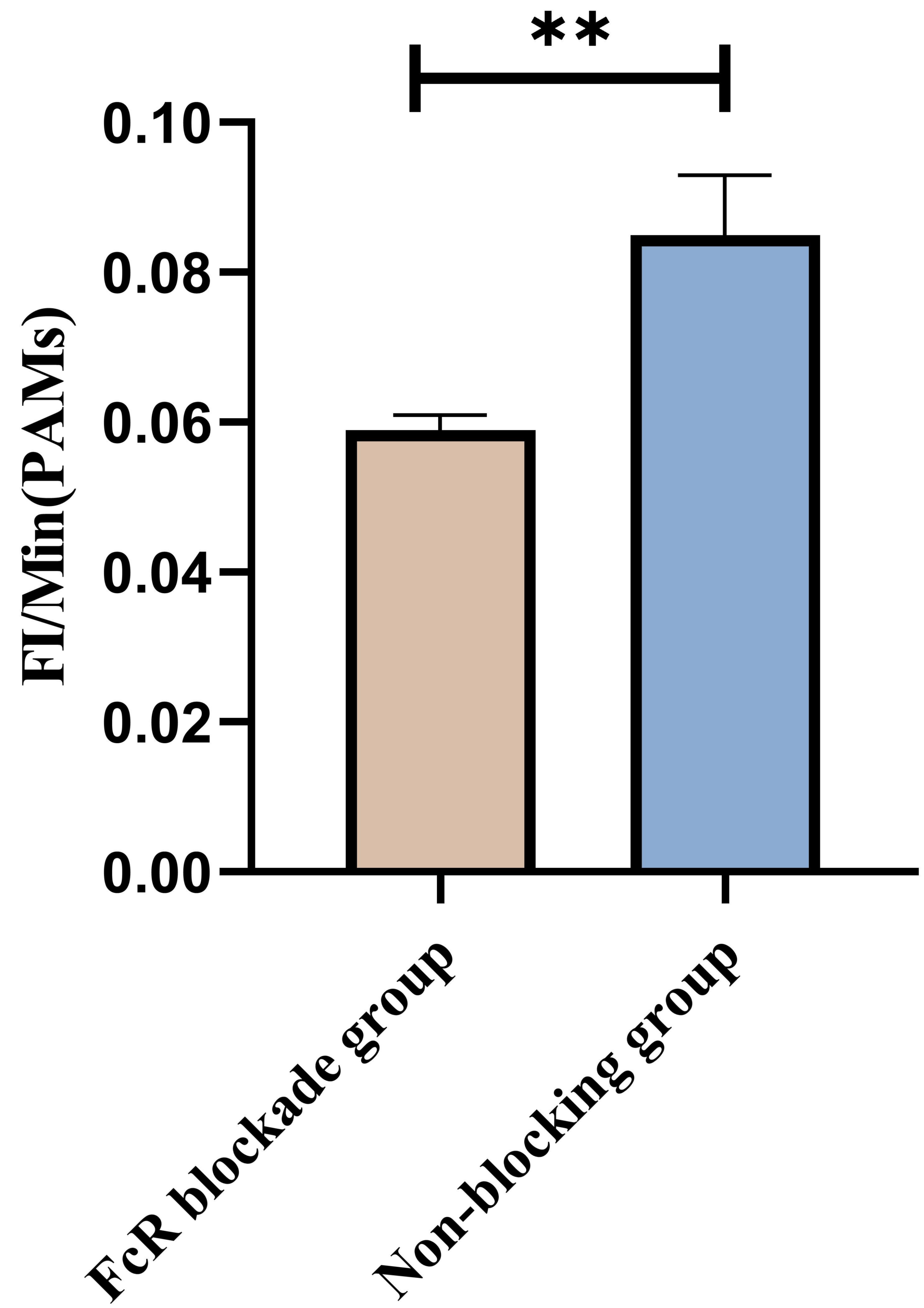

Supplement: Supplemental Information 2 [file peerj-13-18934-s002.zip › Picture supplement/Figure 14/Figure 14.pdf]

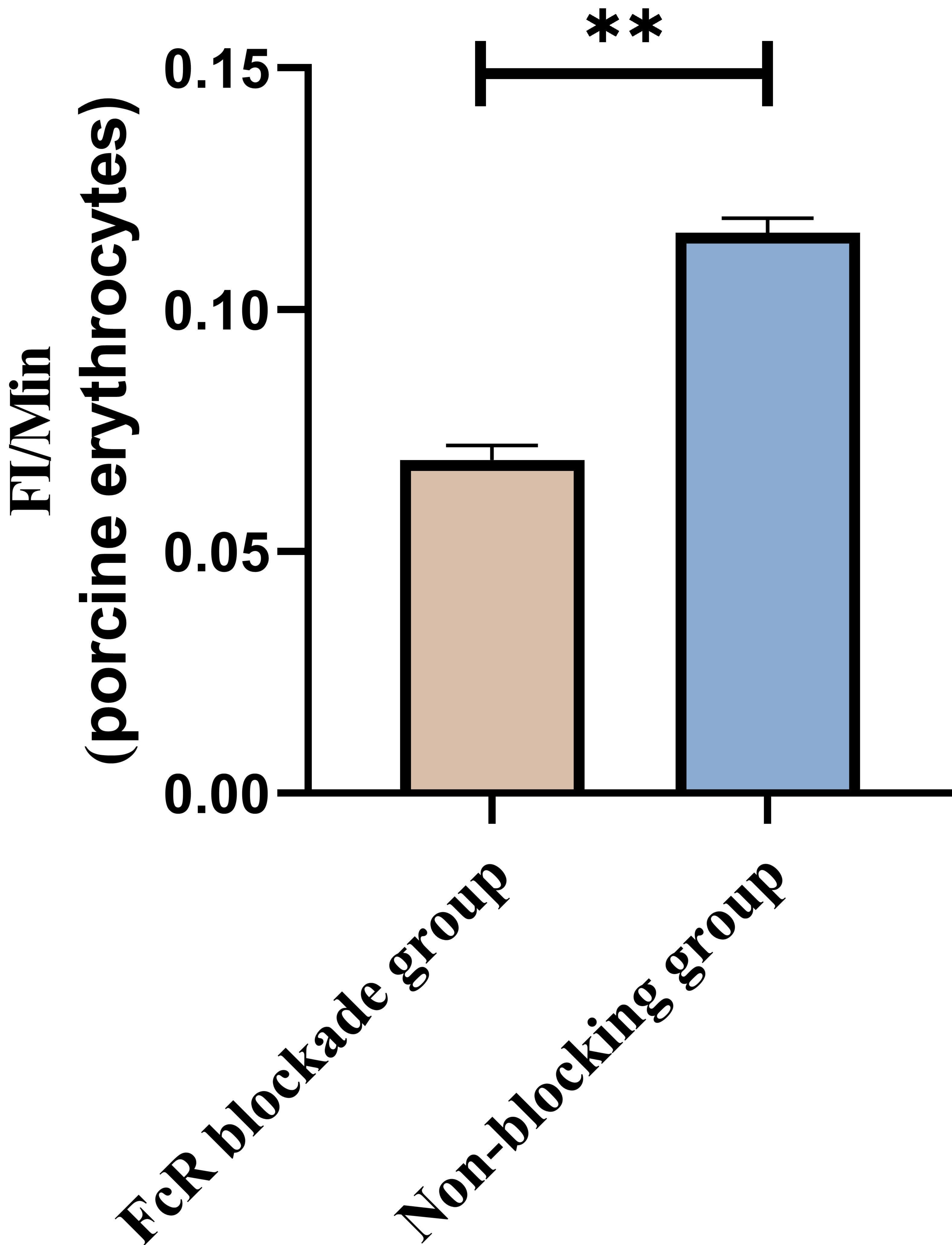

Supplement: Supplemental Information 2 [file peerj-13-18934-s002.zip › Picture supplement/Figure 14/Figure 14A.pdf]

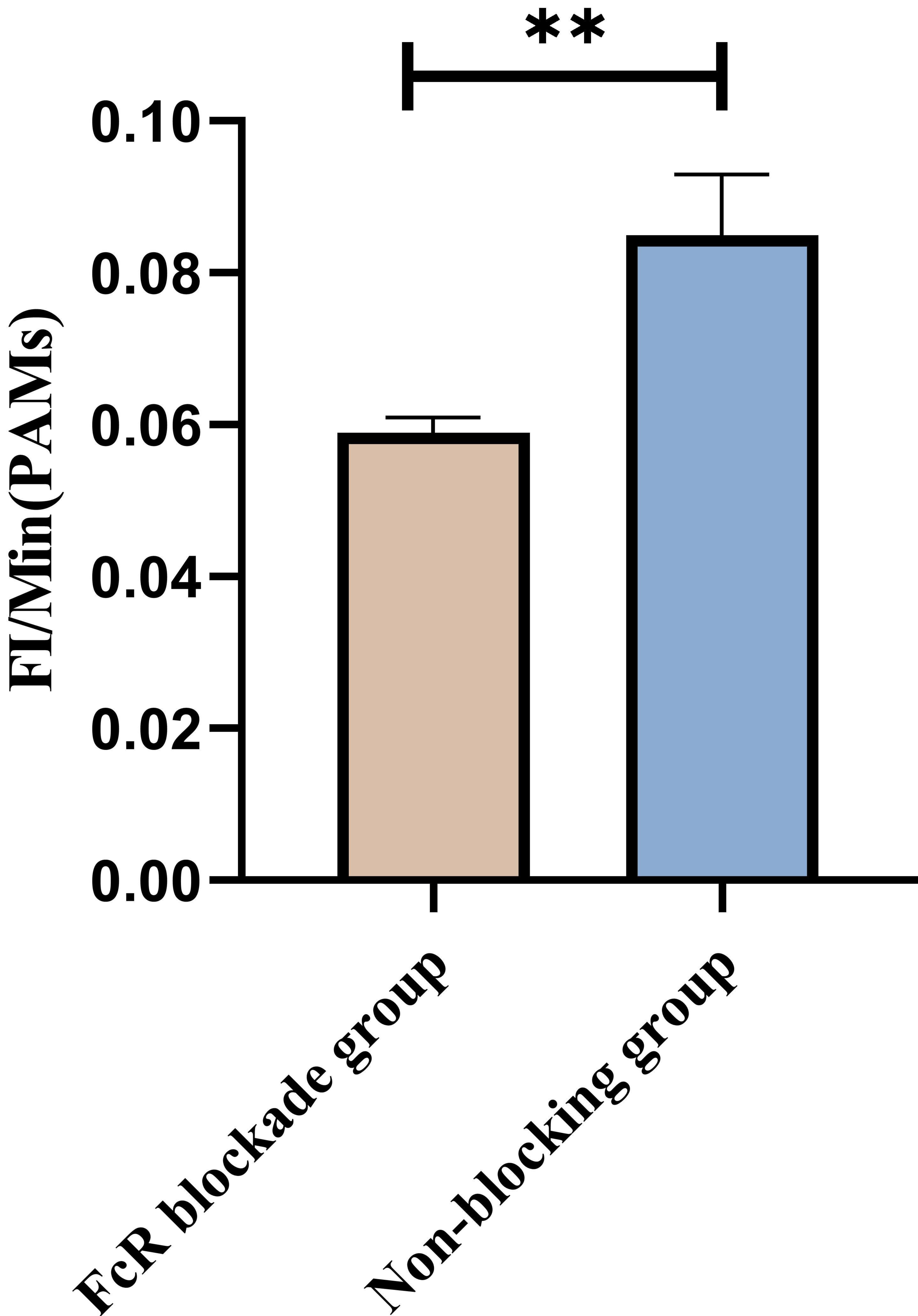

Supplement: Supplemental Information 2 [file peerj-13-18934-s002.zip › Picture supplement/Figure 14/Figure 14B.pdf]

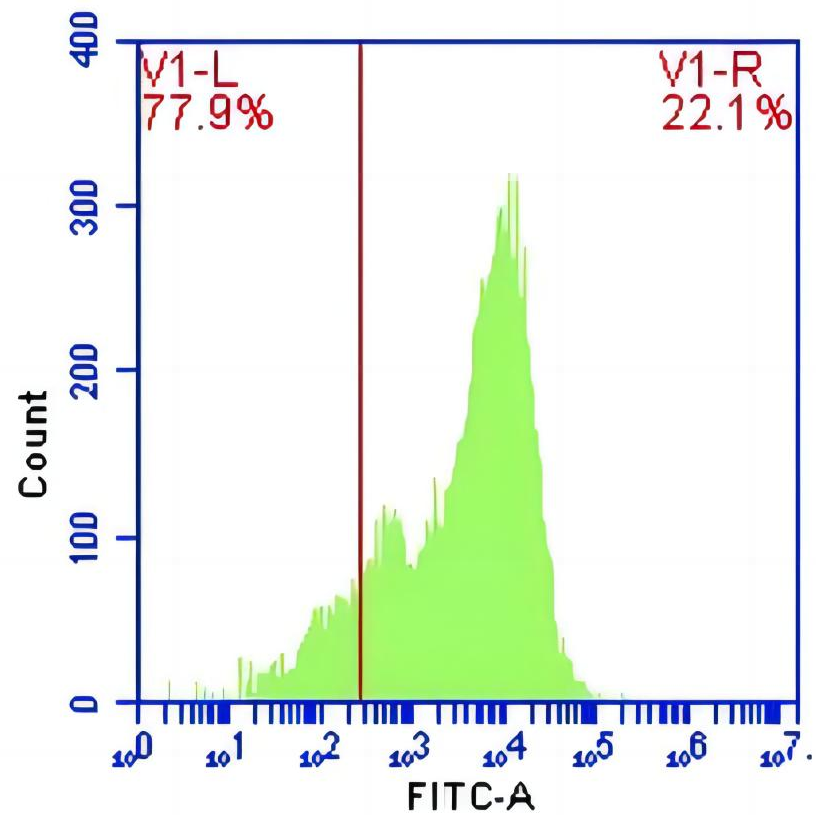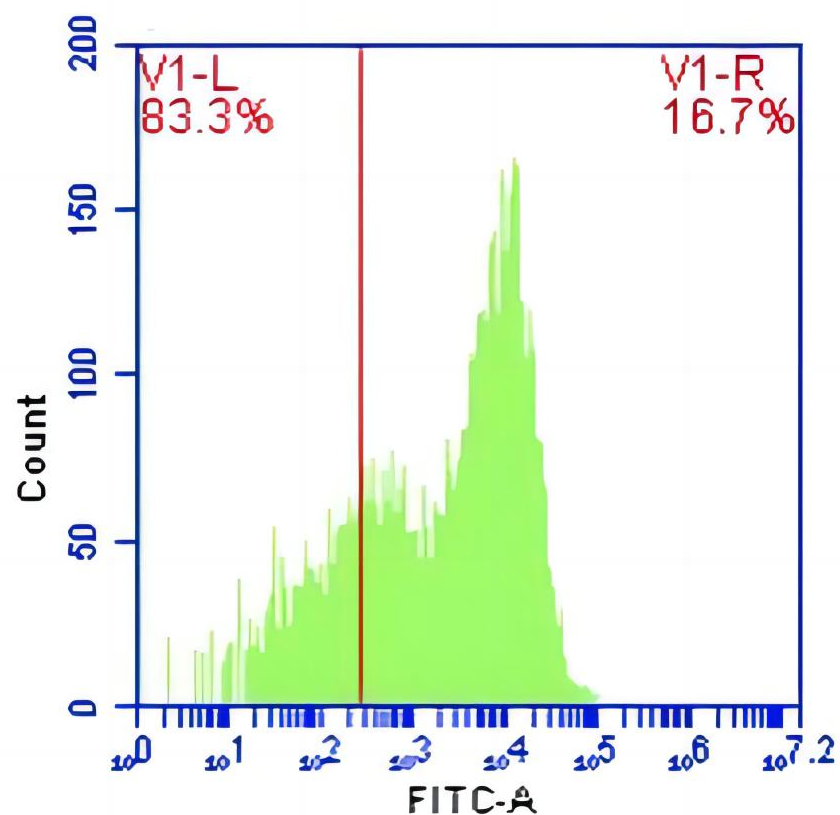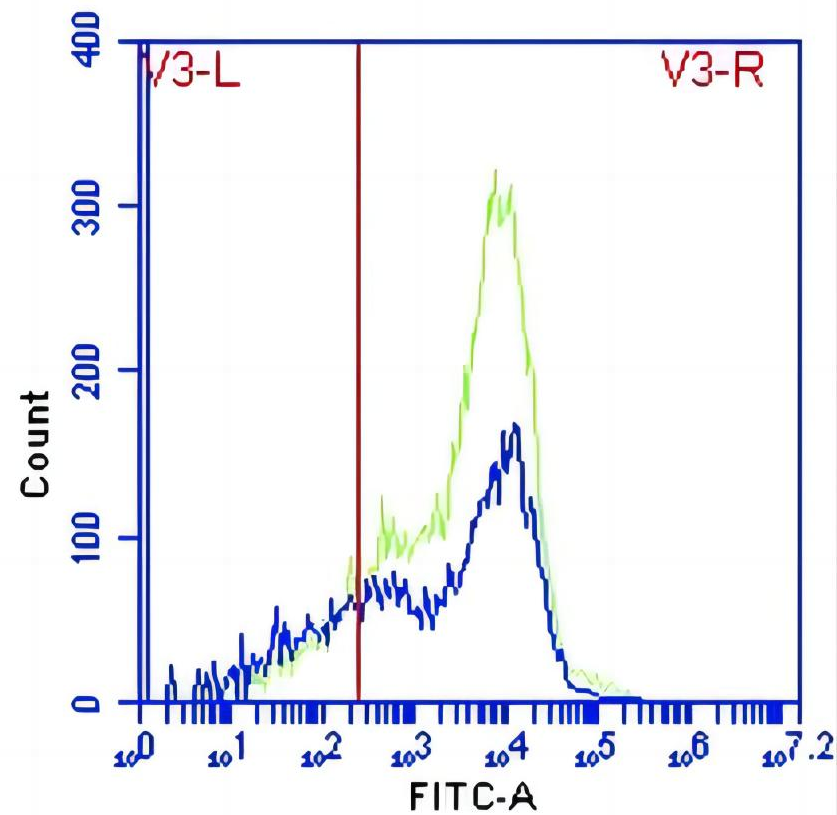

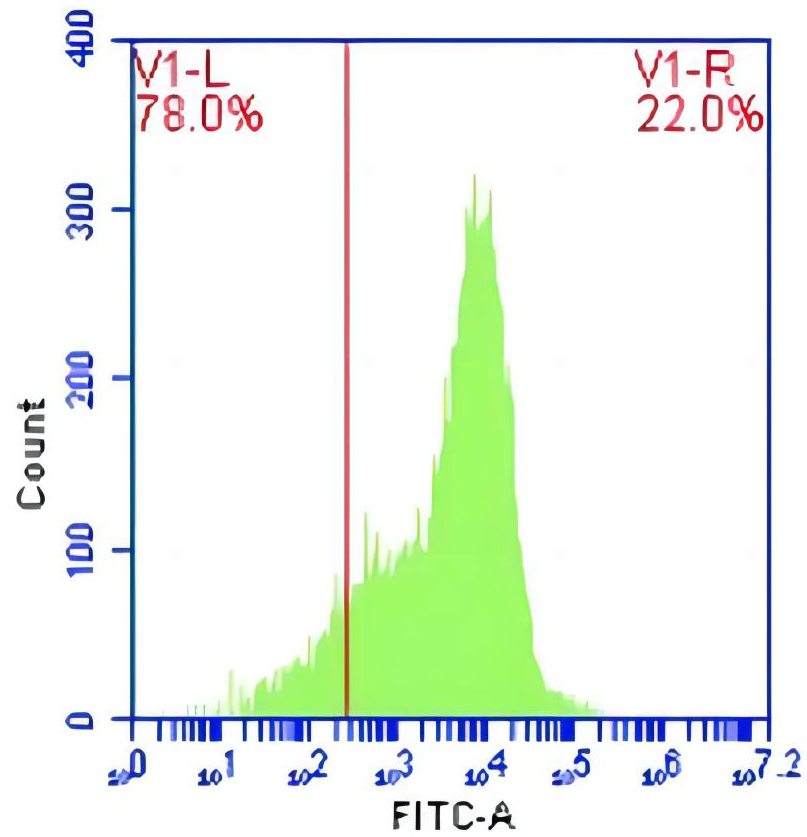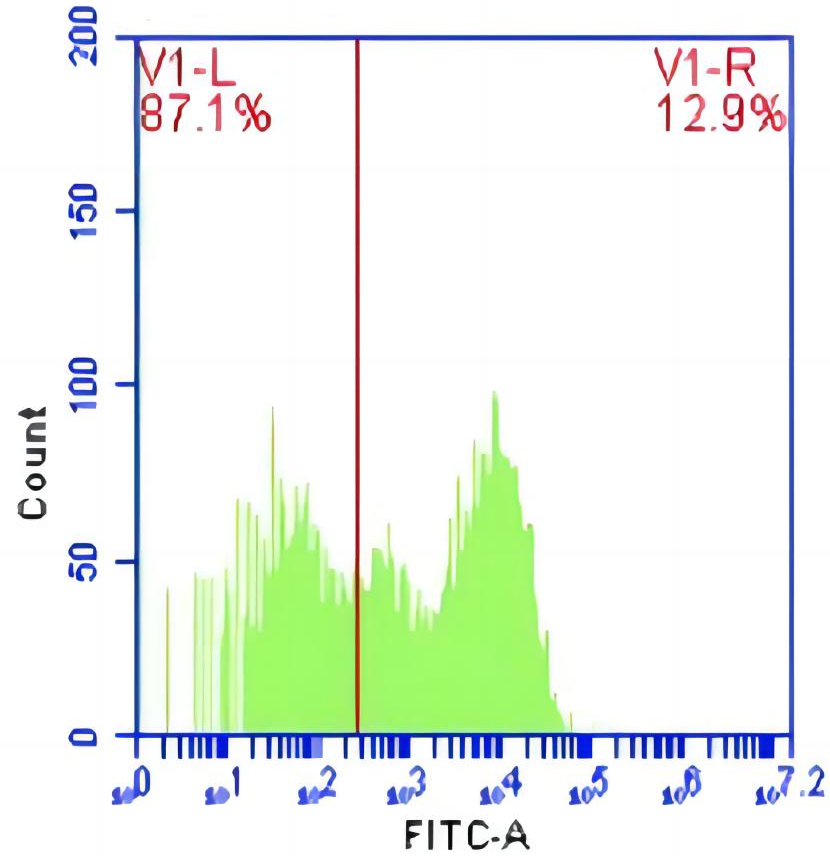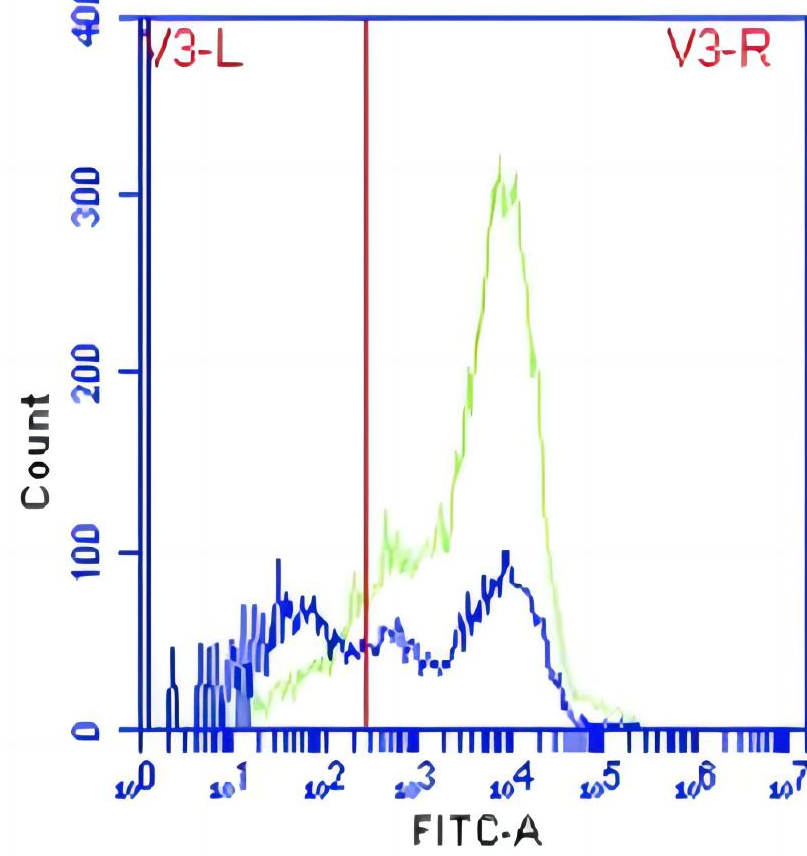

Supplement: Supplemental Information 2 [file peerj-13-18934-s002.zip › Picture supplement/Figure 15/Figure 15.pdf]

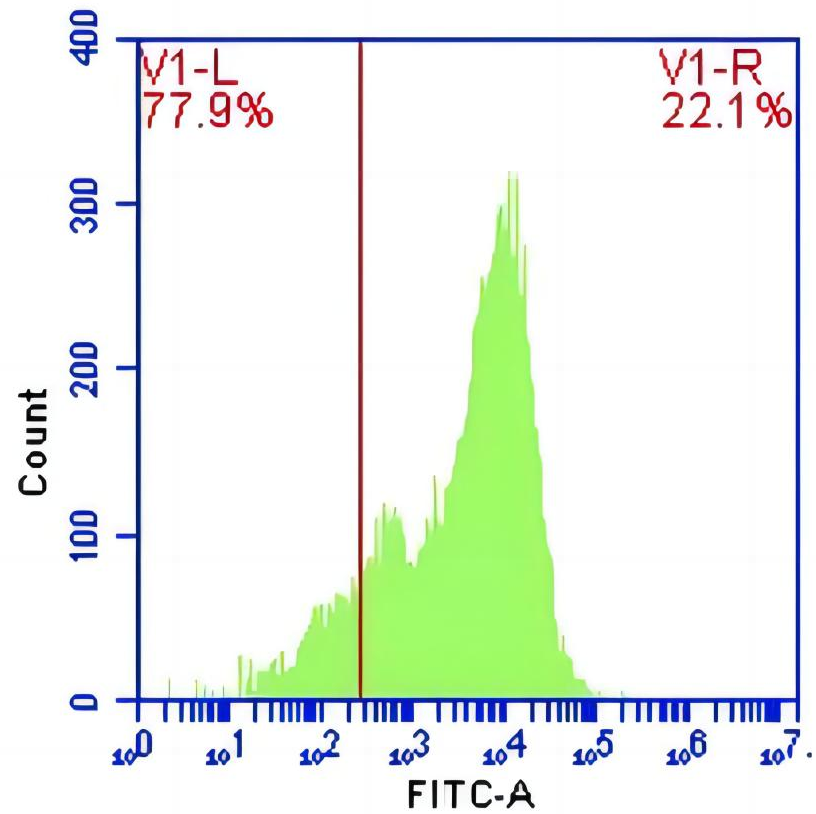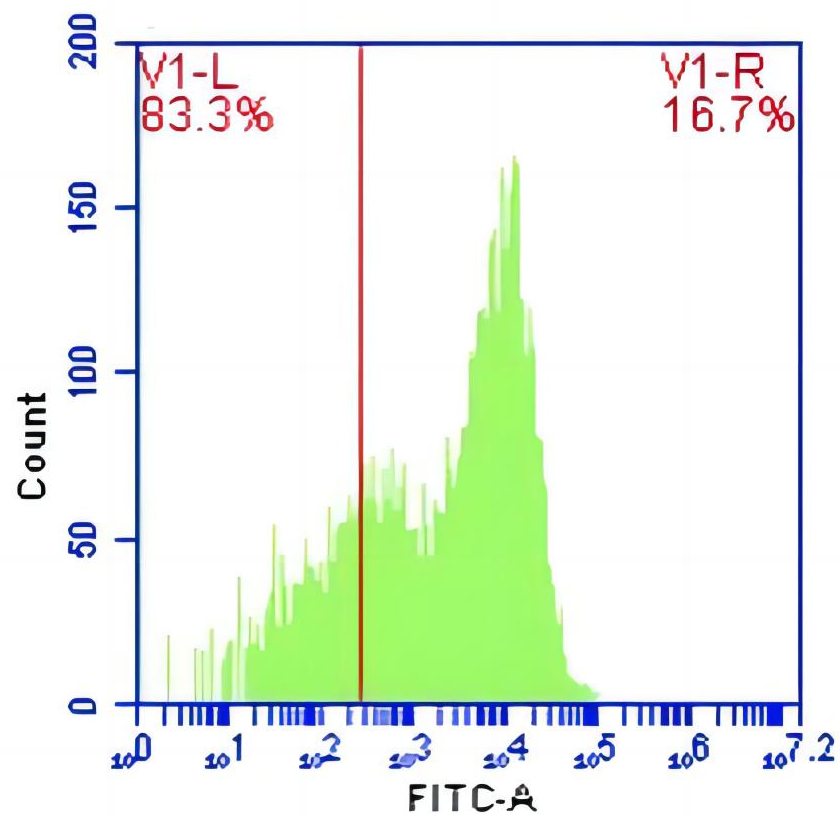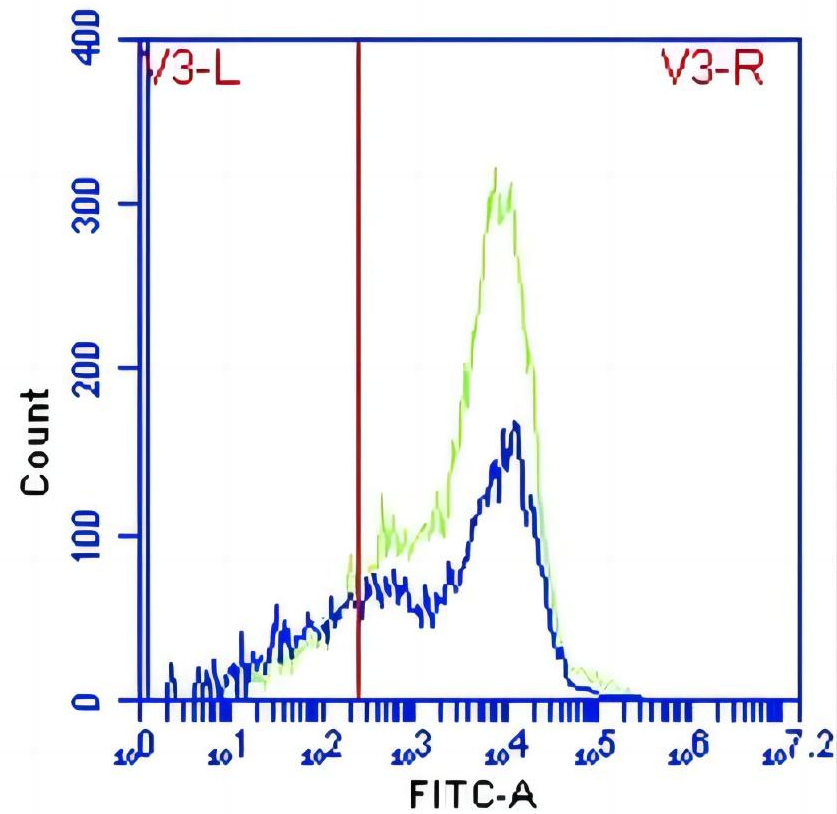

Supplement: Supplemental Information 2 [file peerj-13-18934-s002.zip › Picture supplement/Figure 15/Figure 15A.pdf]

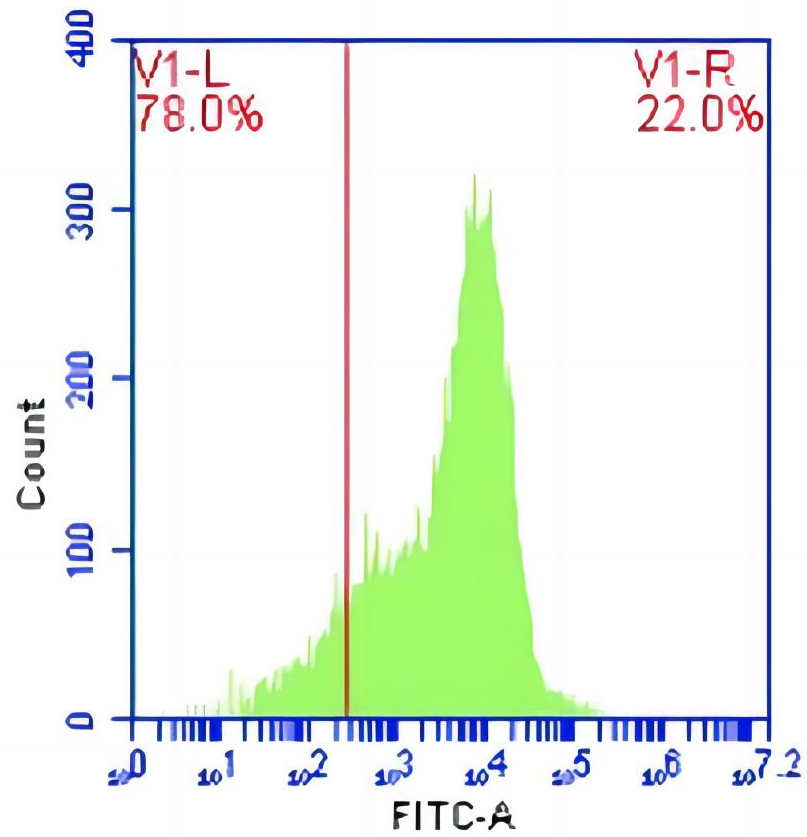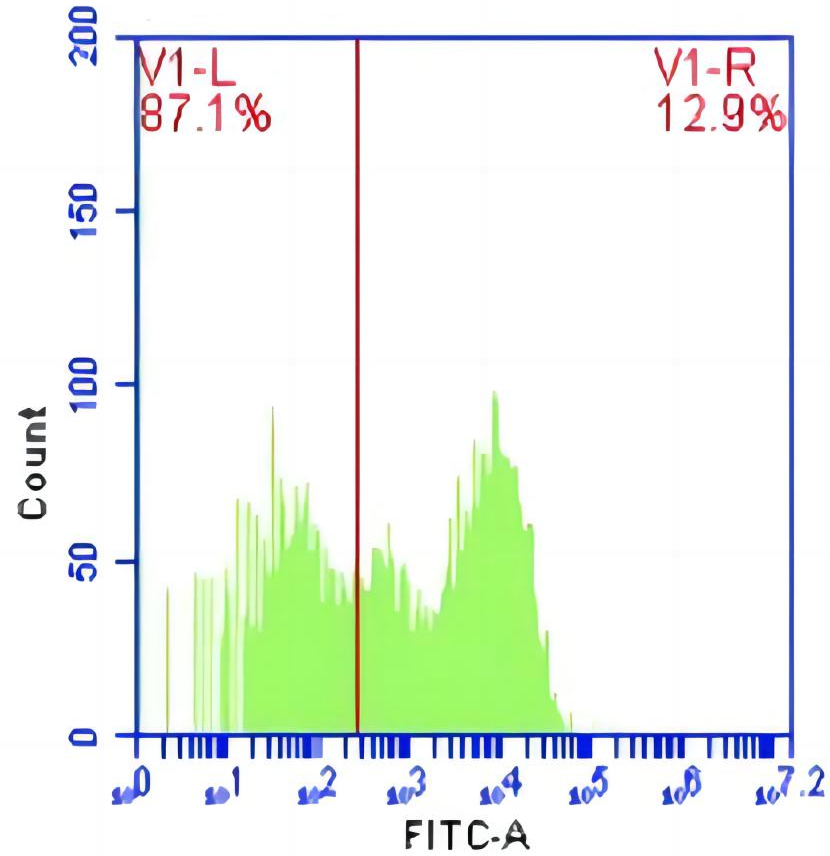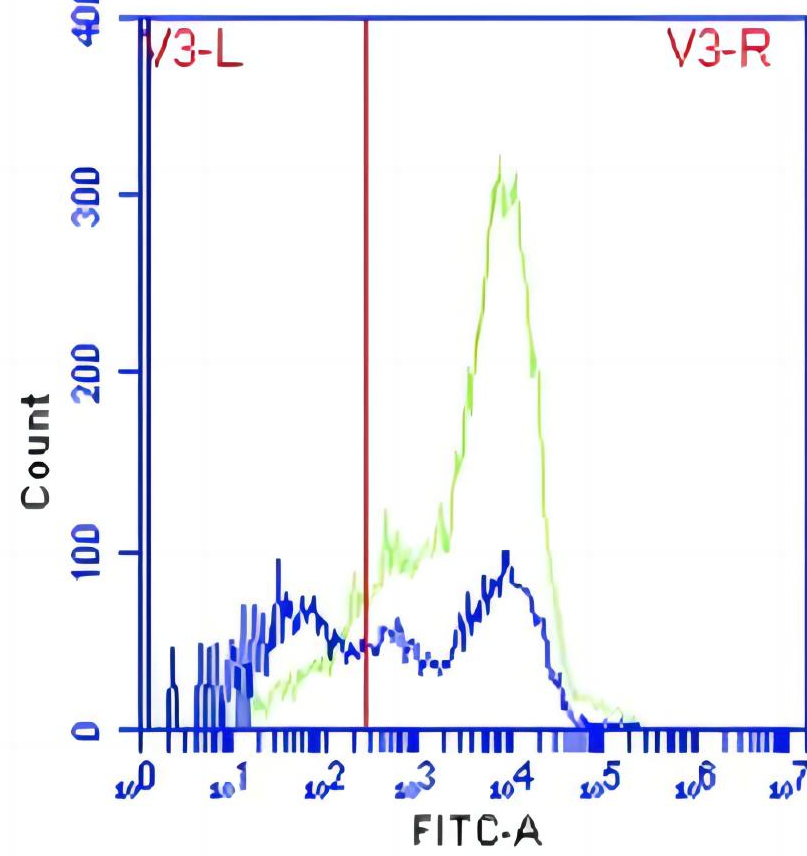

Supplement: Supplemental Information 2 [file peerj-13-18934-s002.zip › Picture supplement/Figure 15/Figure 15B.pdf]

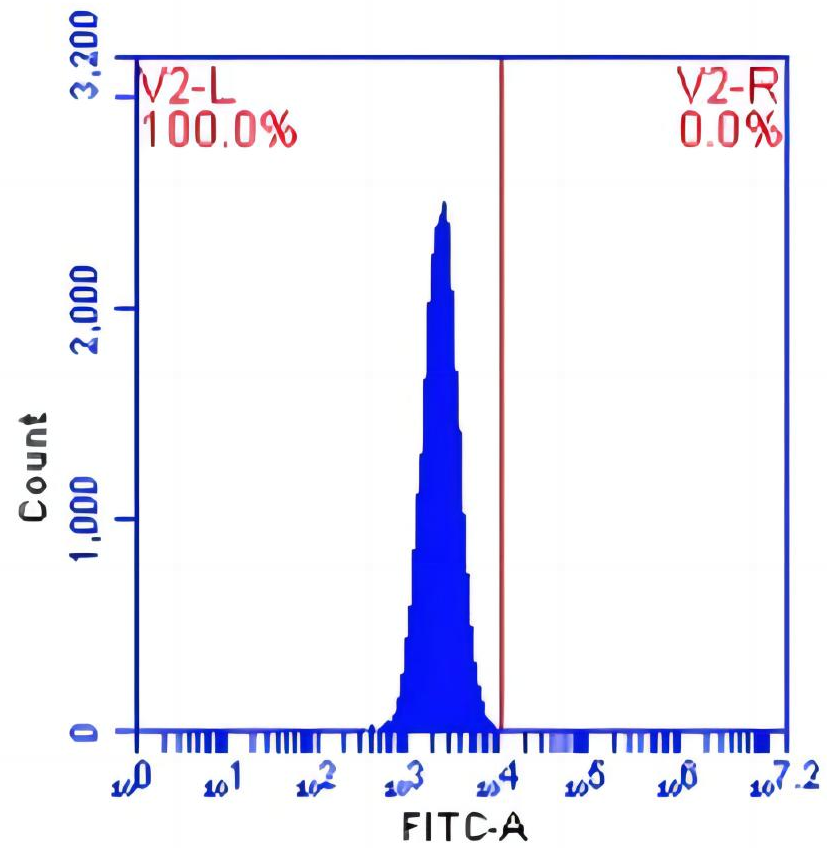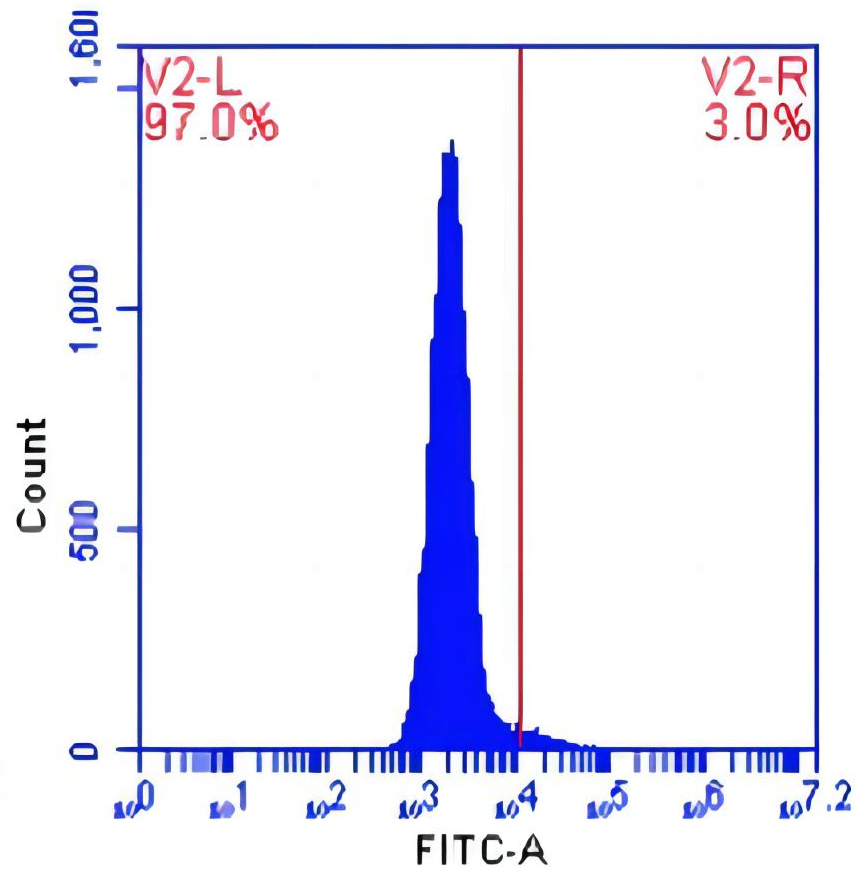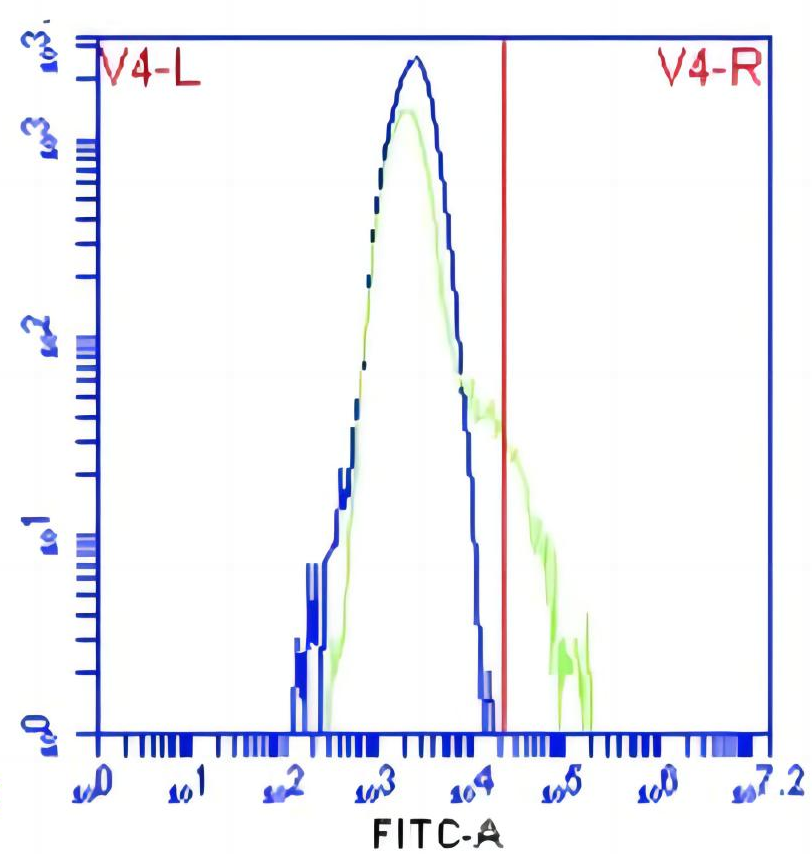

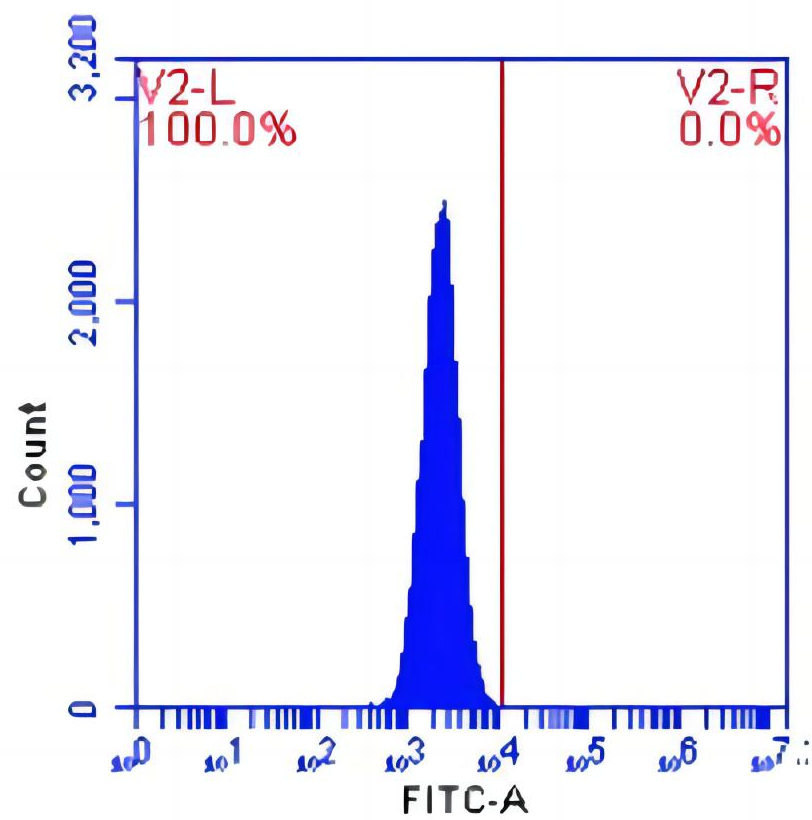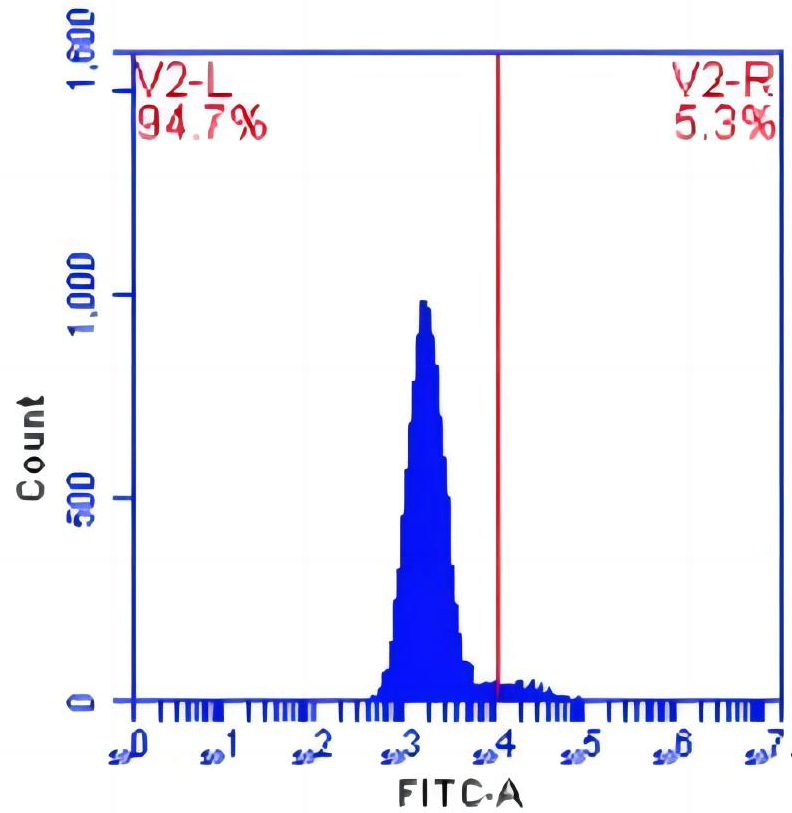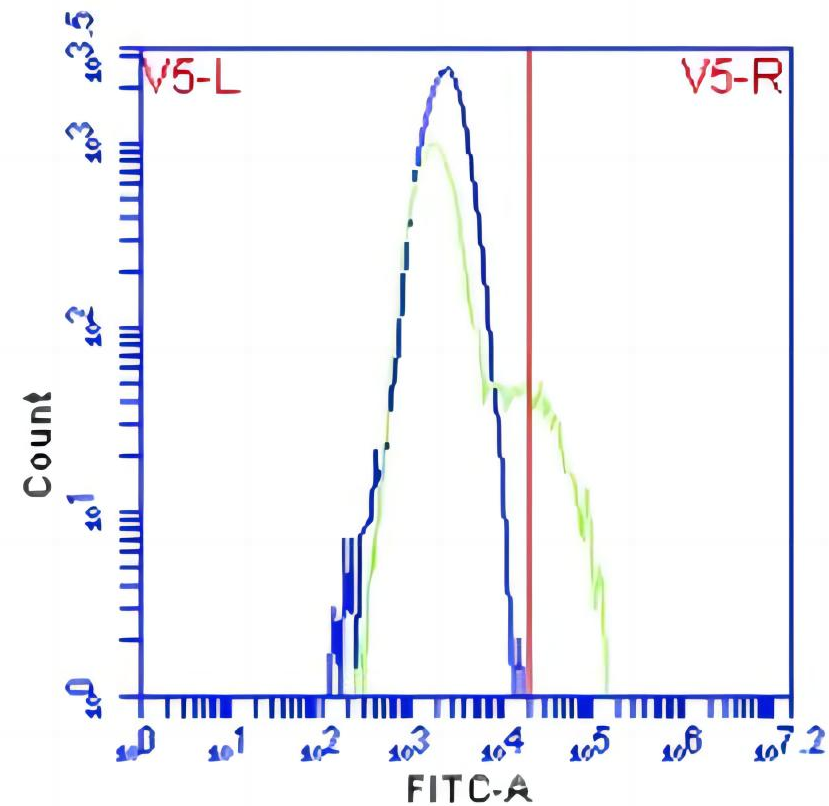

Supplement: Supplemental Information 2 [file peerj-13-18934-s002.zip › Picture supplement/Figure 16/Figure 16.pdf]

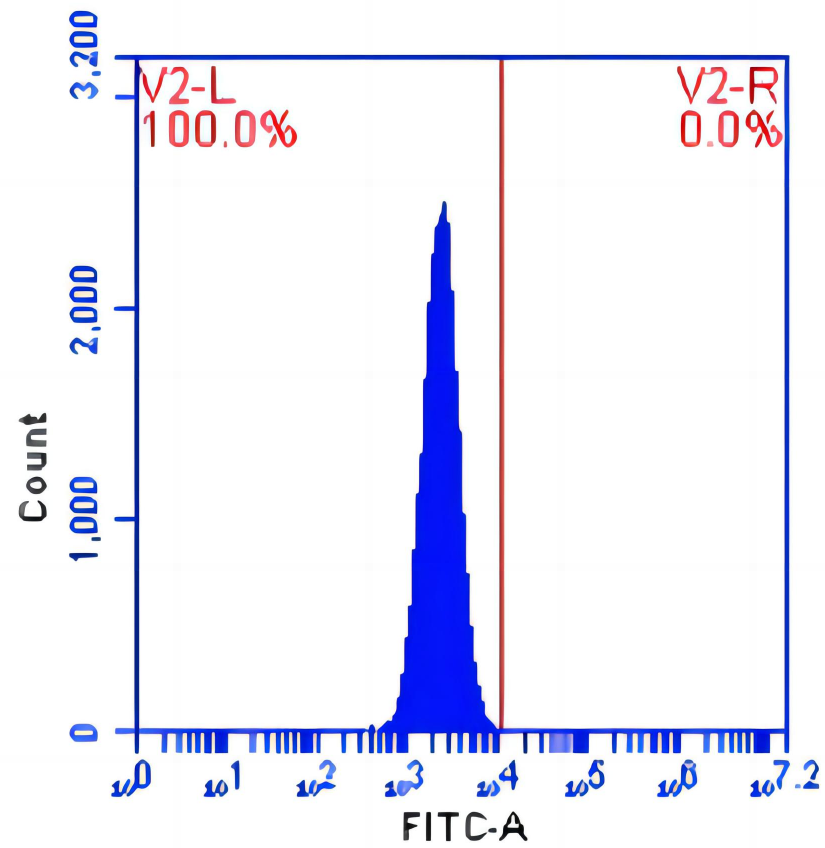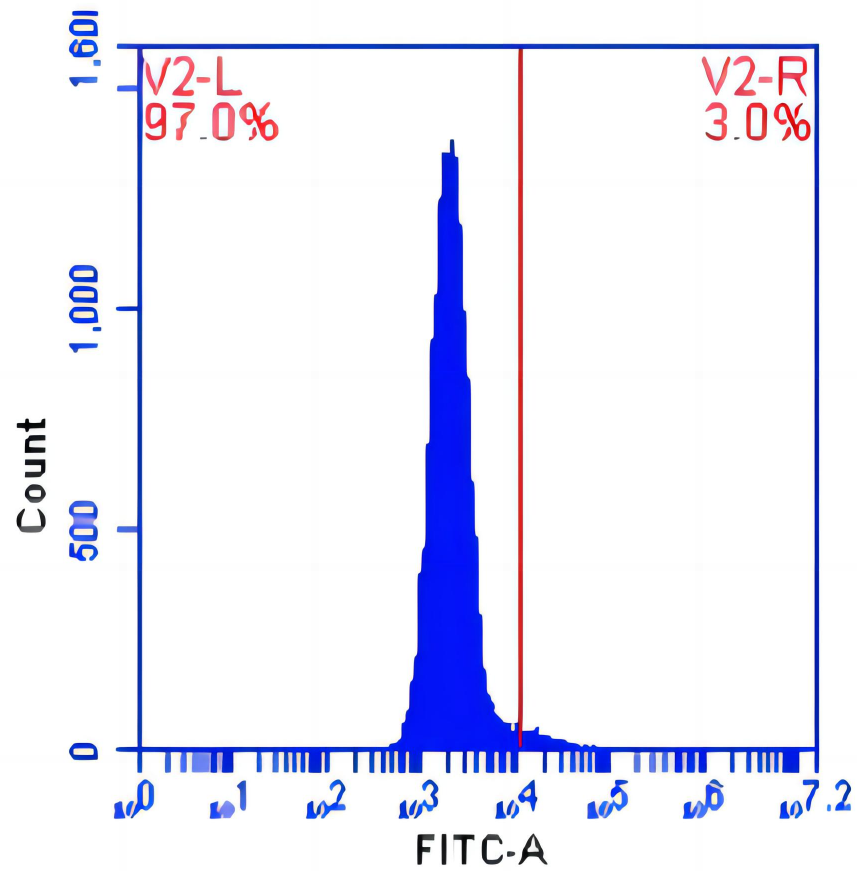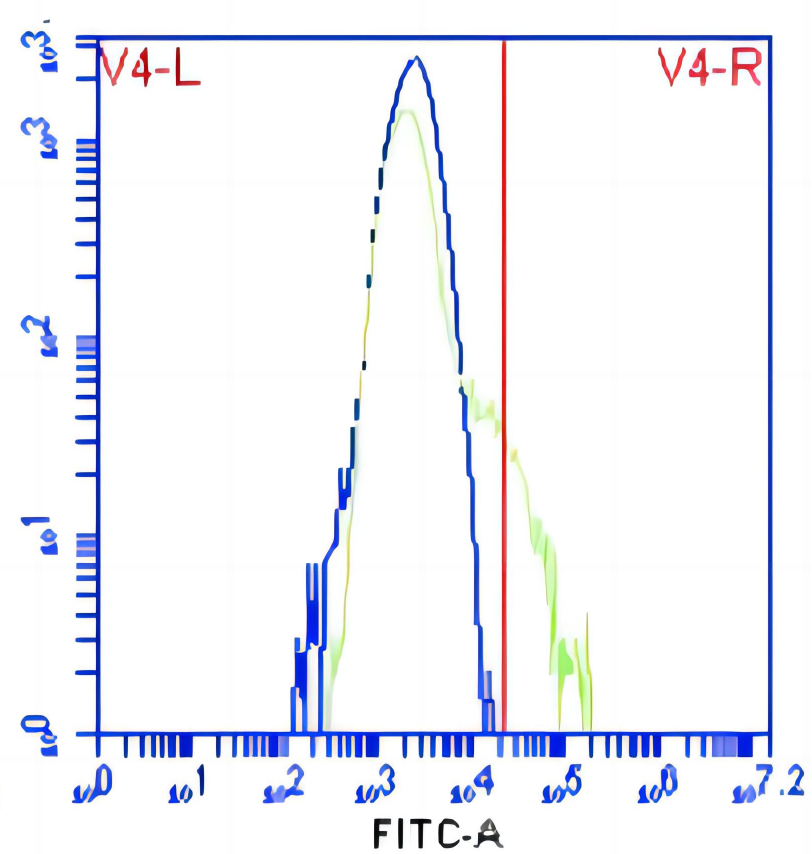

Supplement: Supplemental Information 2 [file peerj-13-18934-s002.zip › Picture supplement/Figure 16/Figure 16A.pdf]

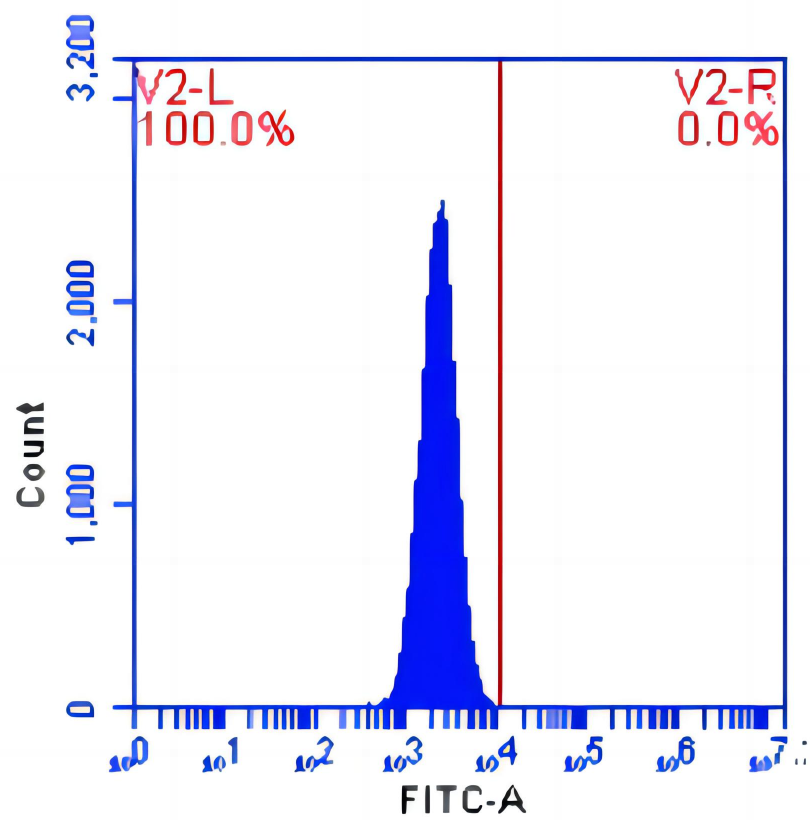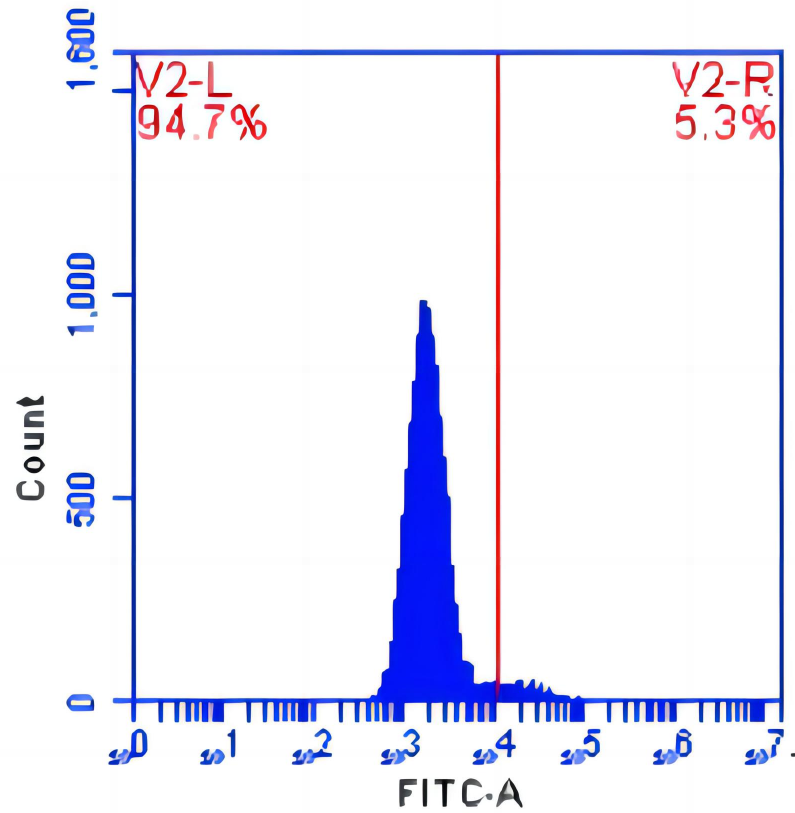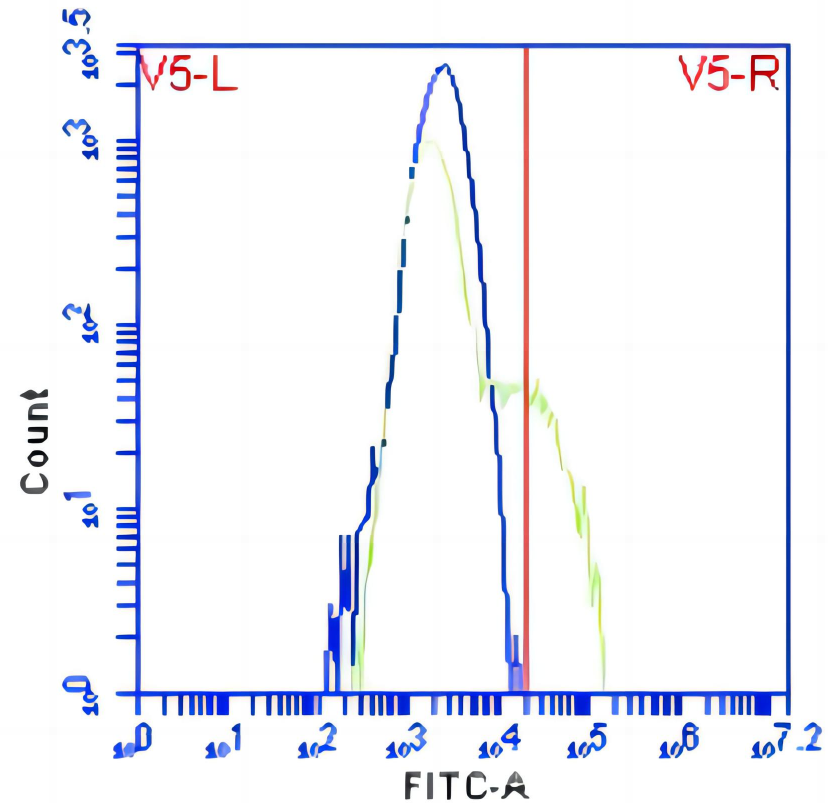

Supplement: Supplemental Information 2 [file peerj-13-18934-s002.zip › Picture supplement/Figure 16/Figure 16B.pdf]

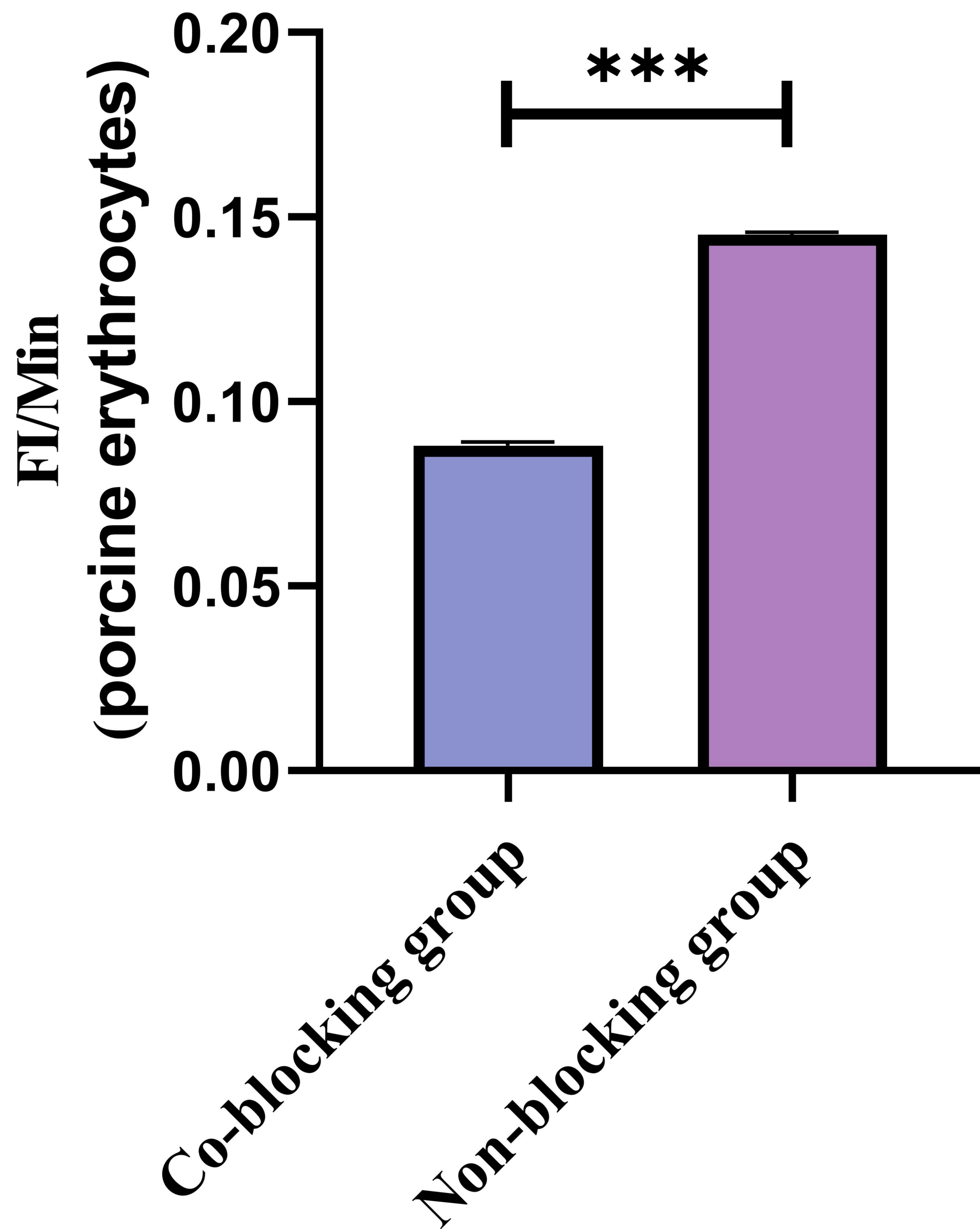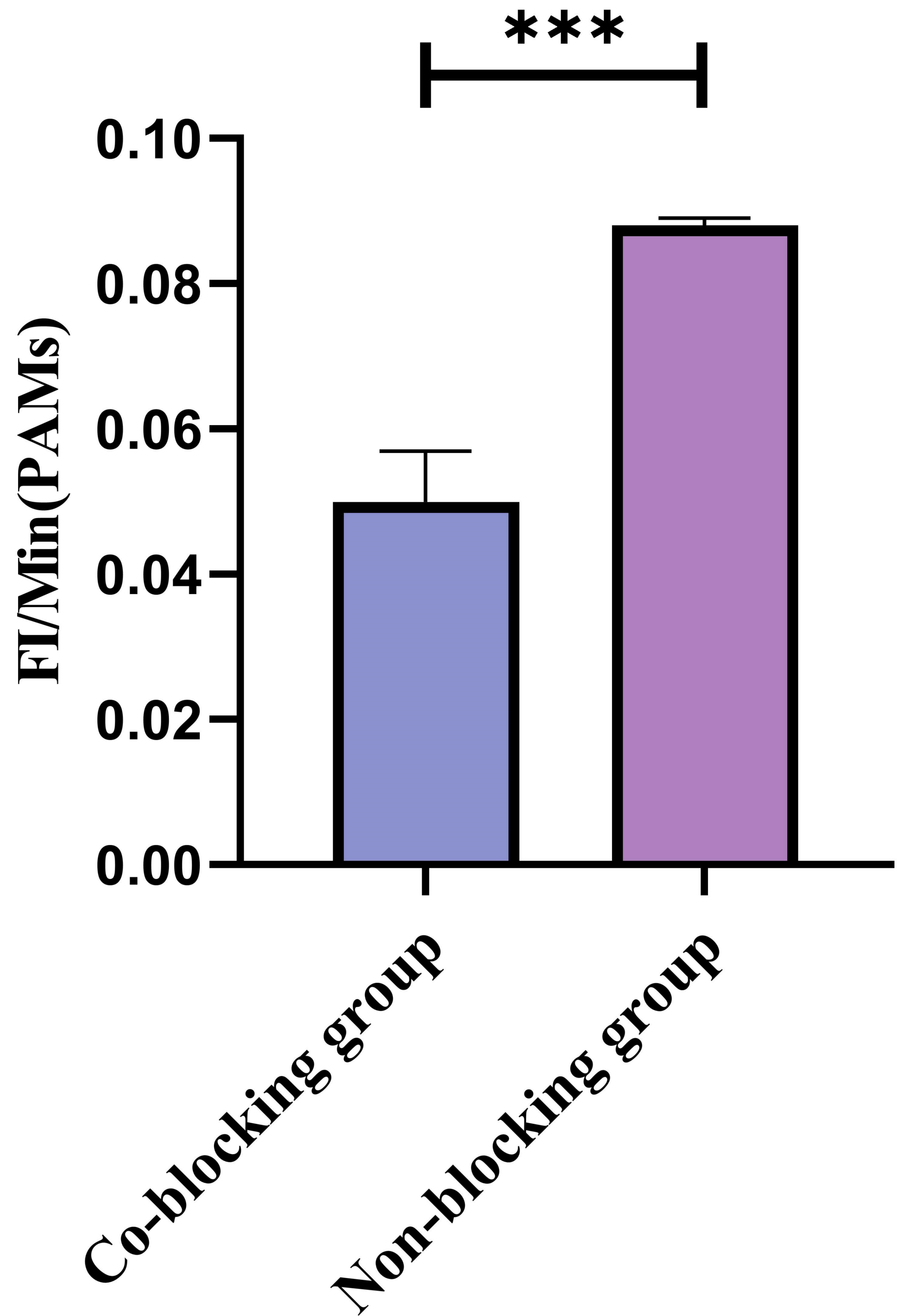

Supplement: Supplemental Information 2 [file peerj-13-18934-s002.zip › Picture supplement/Figure 17/Figure 17.pdf]

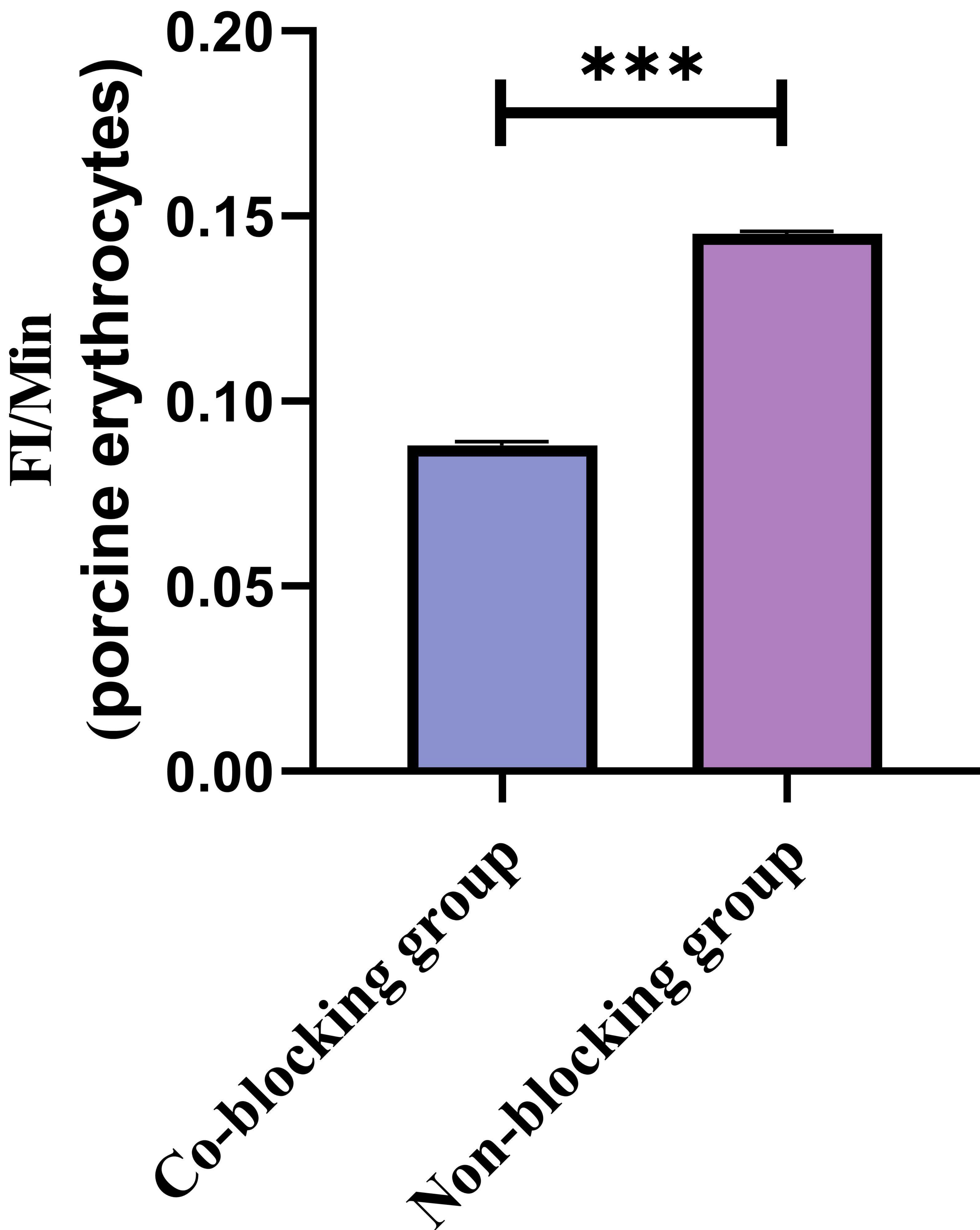

Supplement: Supplemental Information 2 [file peerj-13-18934-s002.zip › Picture supplement/Figure 17/Figure 17A.pdf]

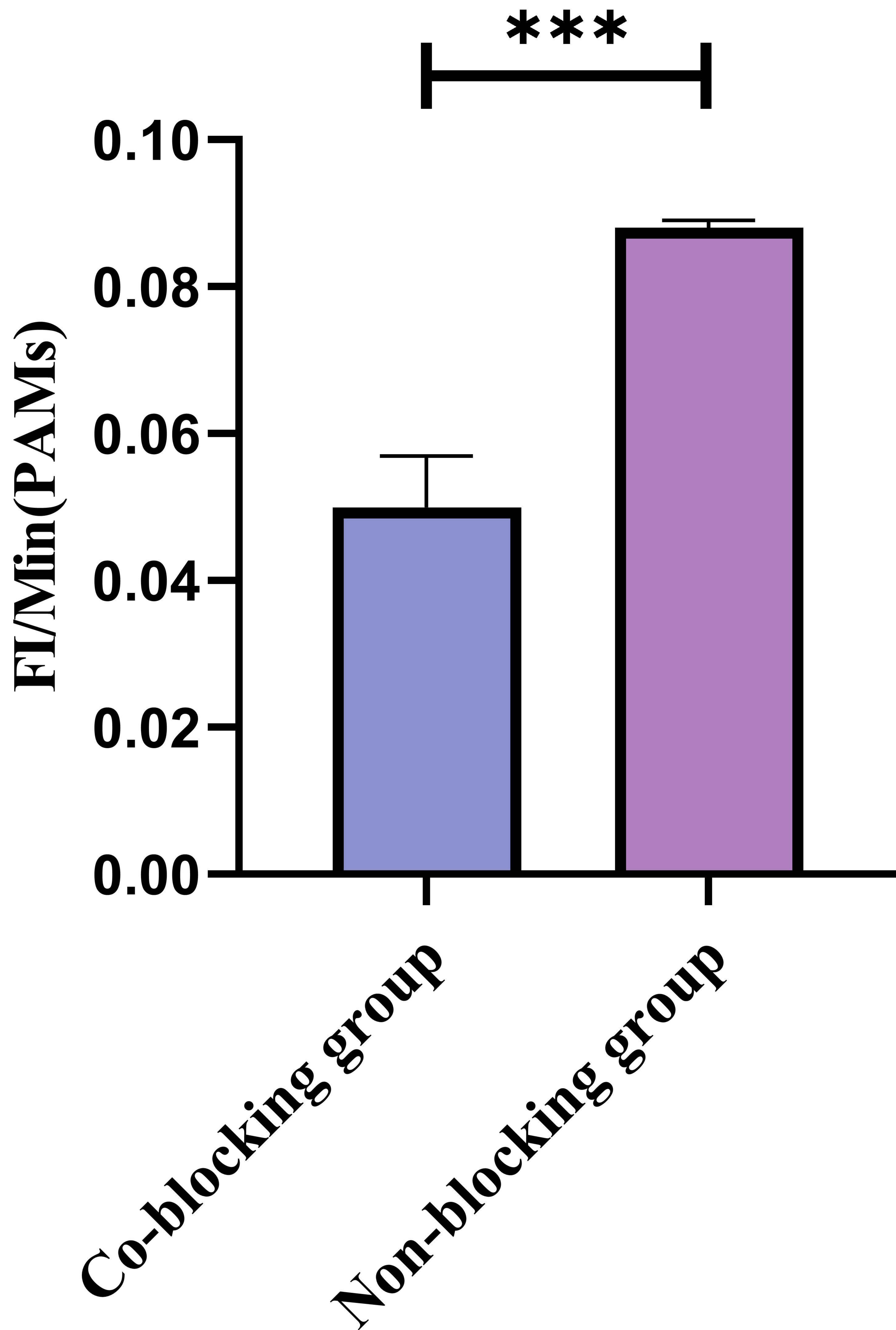

Supplement: Supplemental Information 2 [file peerj-13-18934-s002.zip › Picture supplement/Figure 17/Figure 17B.pdf]

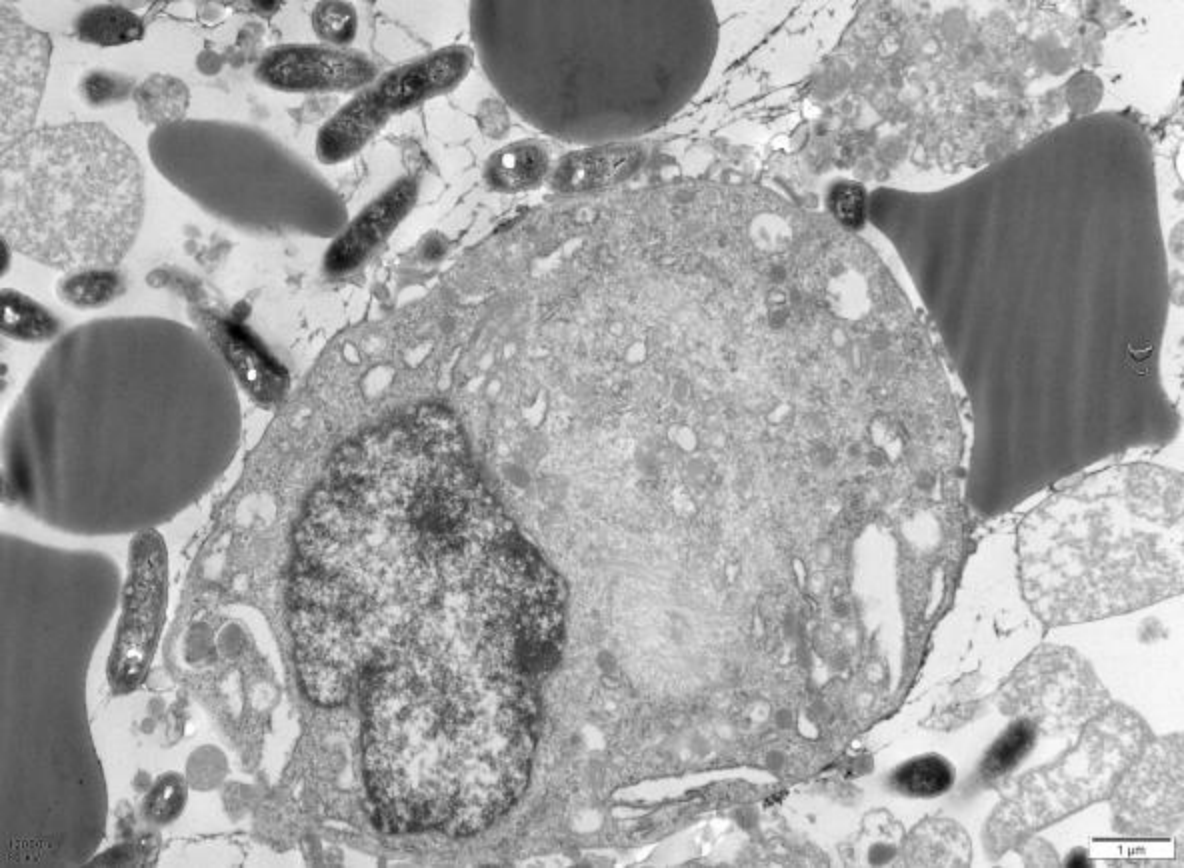

Supplement: Supplemental Information 2 [file peerj-13-18934-s002.zip › Picture supplement/Figure 18/Figure 18-2.pdf]

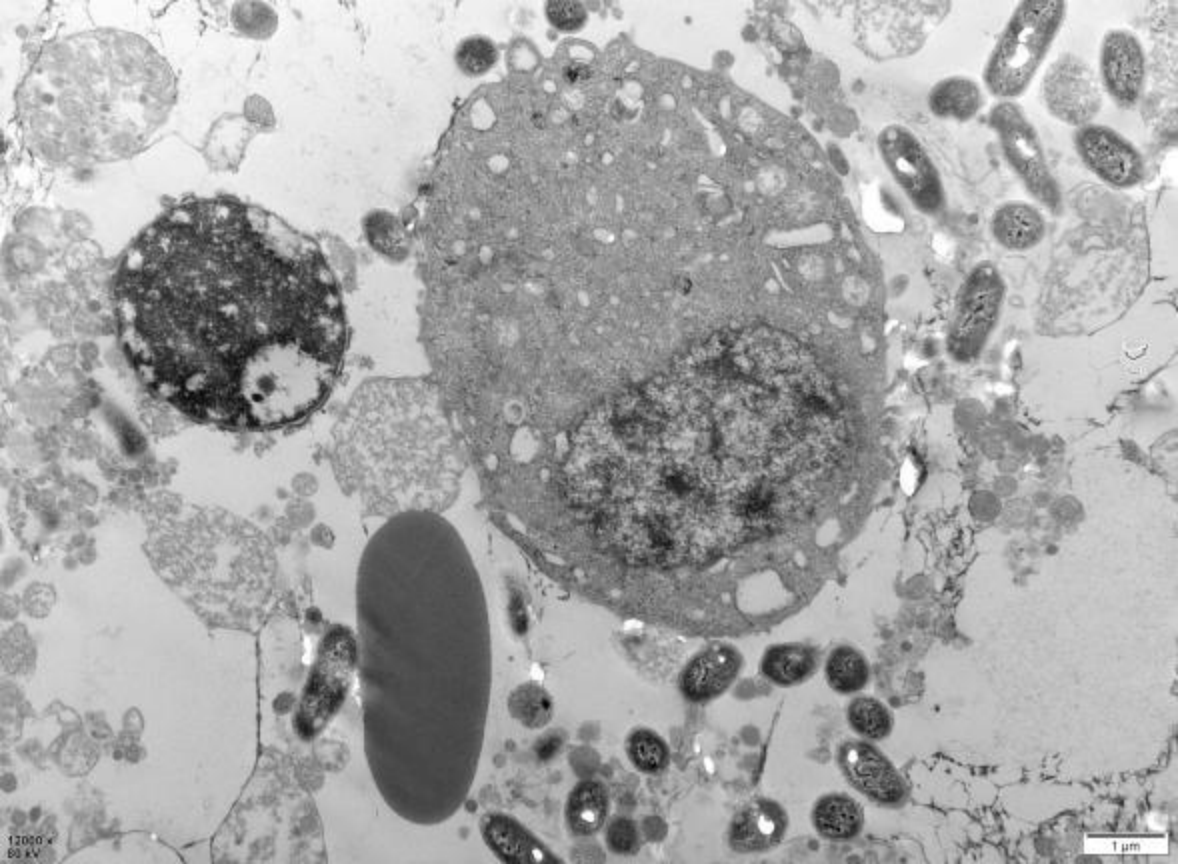

Supplement: Supplemental Information 2 [file peerj-13-18934-s002.zip › Picture supplement/Figure 18/Figure 18-3.pdf]

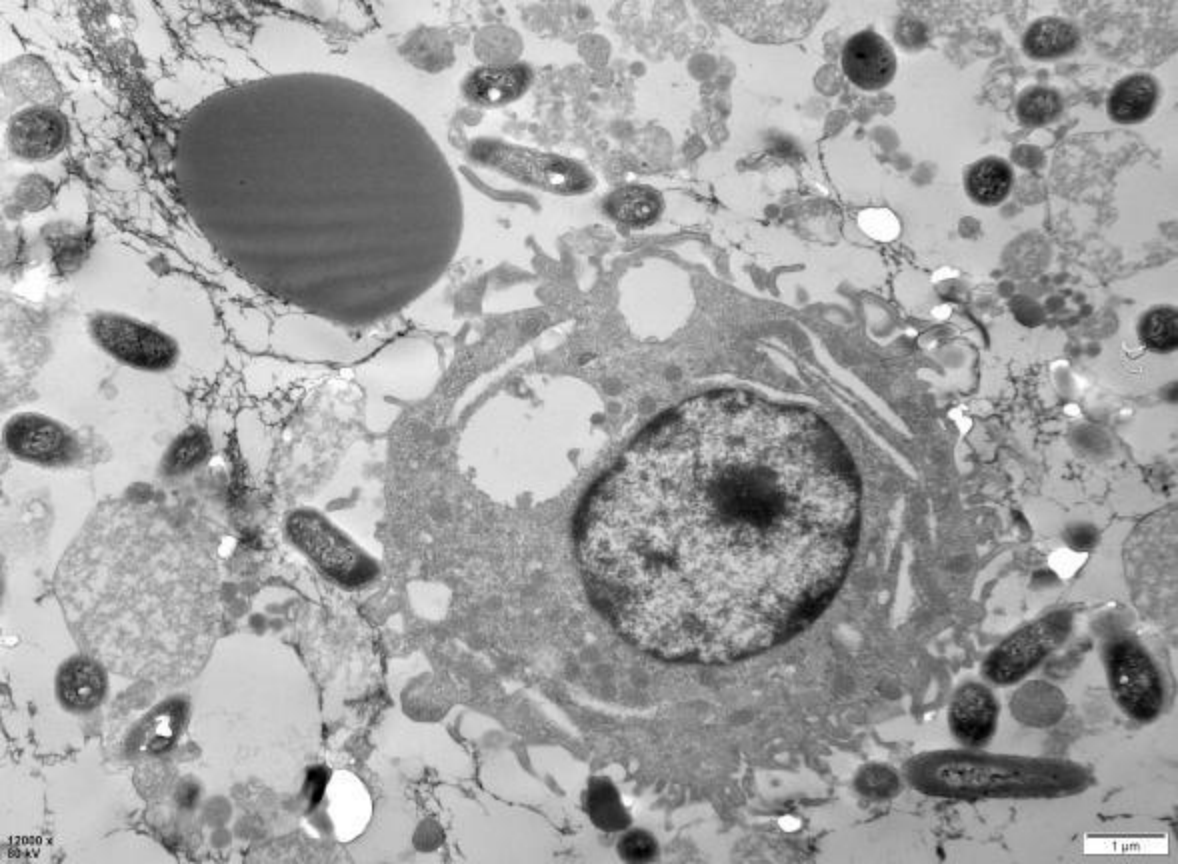

Supplement: Supplemental Information 2 [file peerj-13-18934-s002.zip › Picture supplement/Figure 18/Figure 18.pdf]

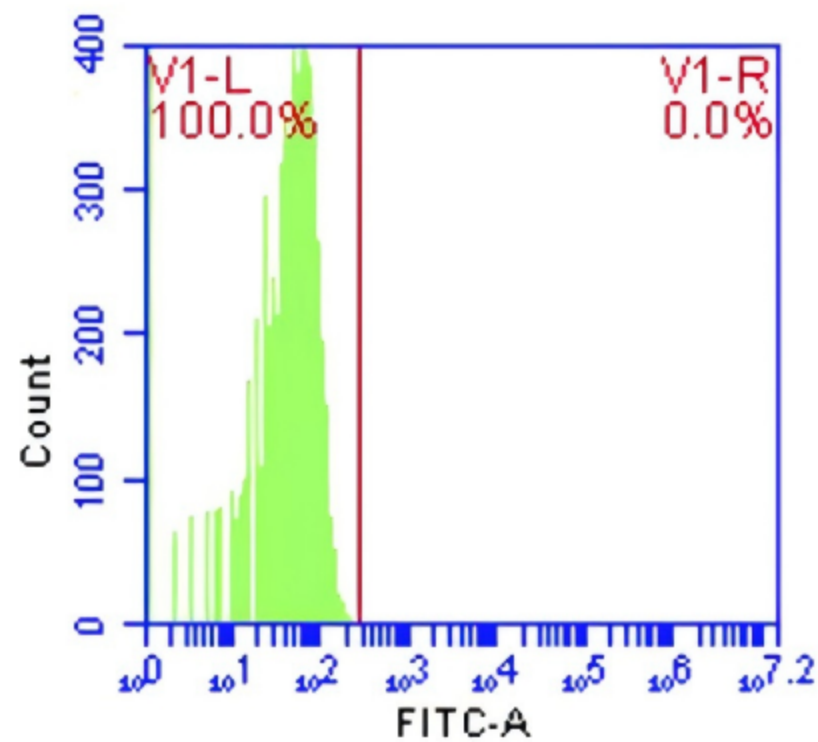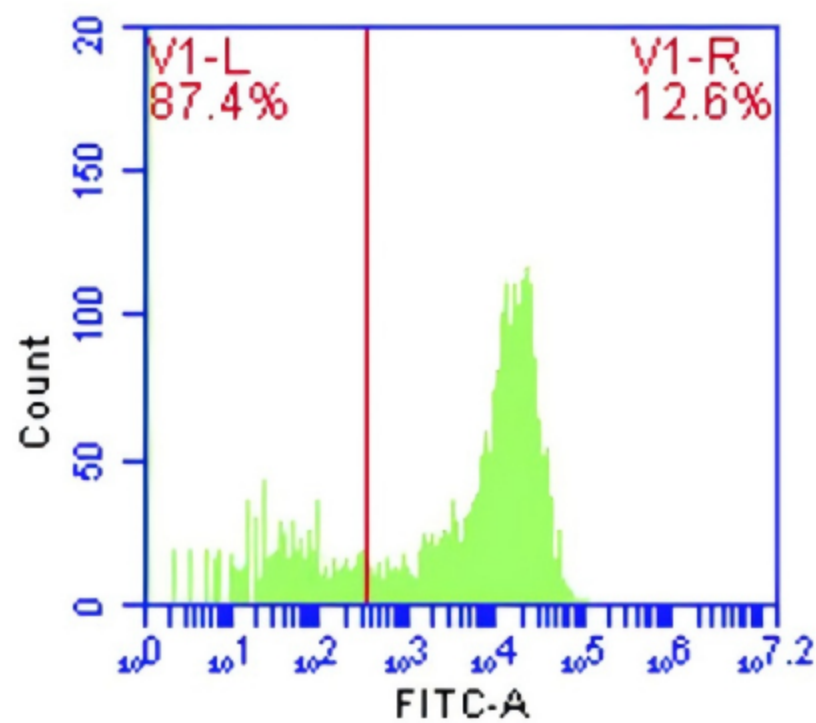

Supplement: Supplemental Information 2 [file peerj-13-18934-s002.zip › Picture supplement/Figure 2/Figure 2-1.pdf]

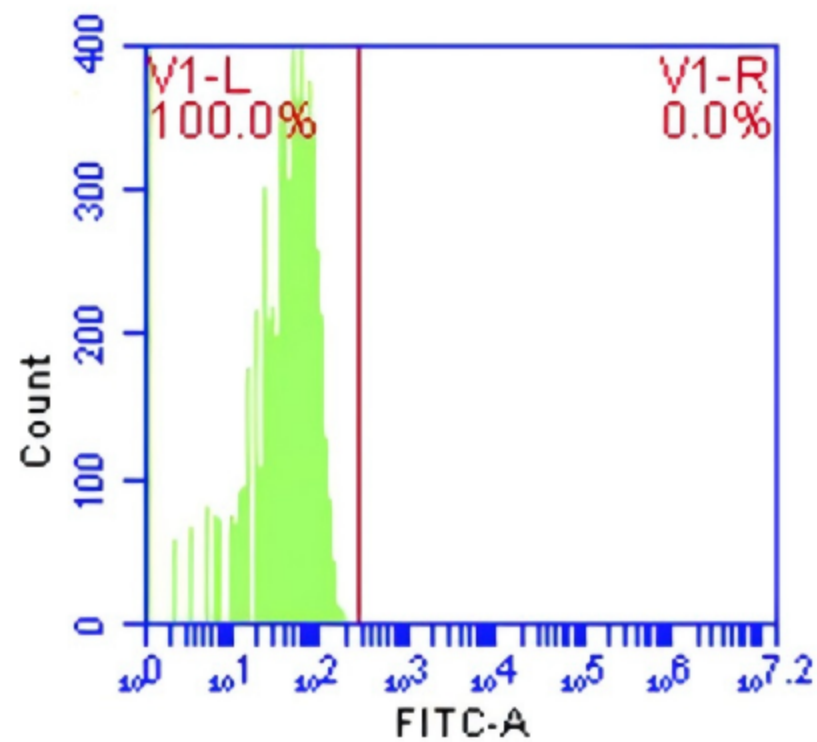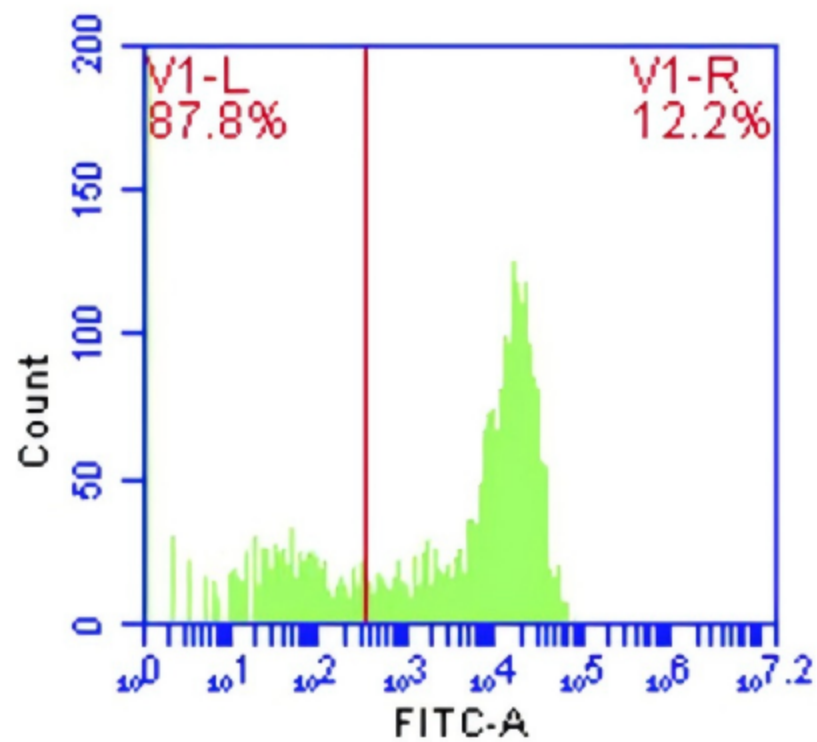

Supplement: Supplemental Information 2 [file peerj-13-18934-s002.zip › Picture supplement/Figure 2/Figure 2-2.pdf]

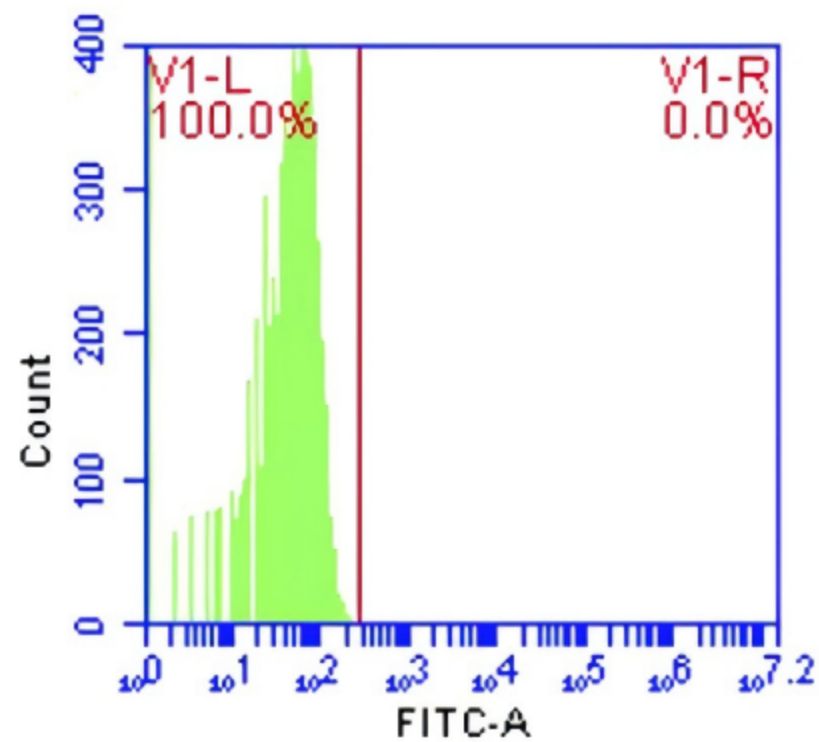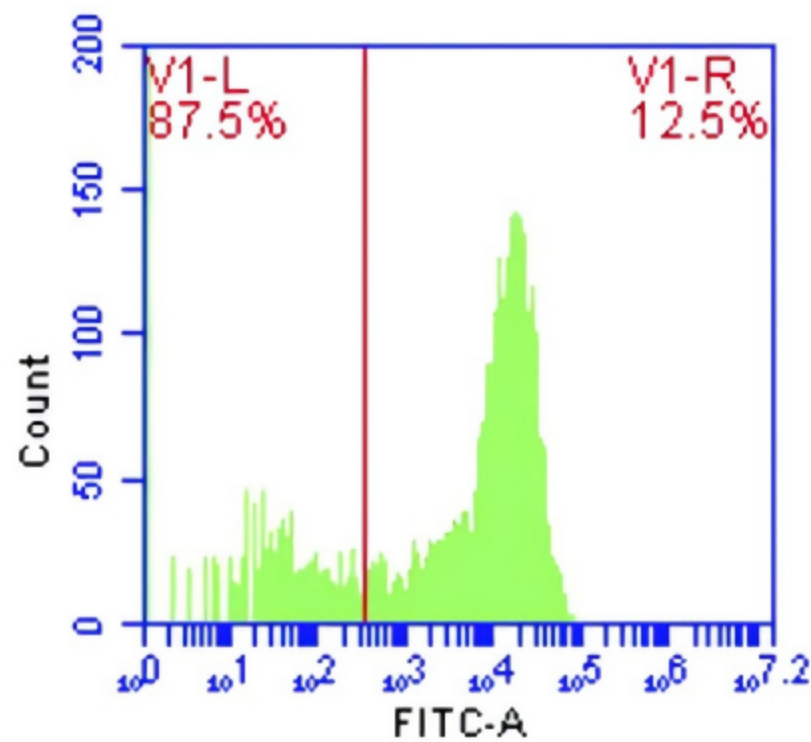

Supplement: Supplemental Information 2 [file peerj-13-18934-s002.zip › Picture supplement/Figure 2/Figure 2-3.pdf]

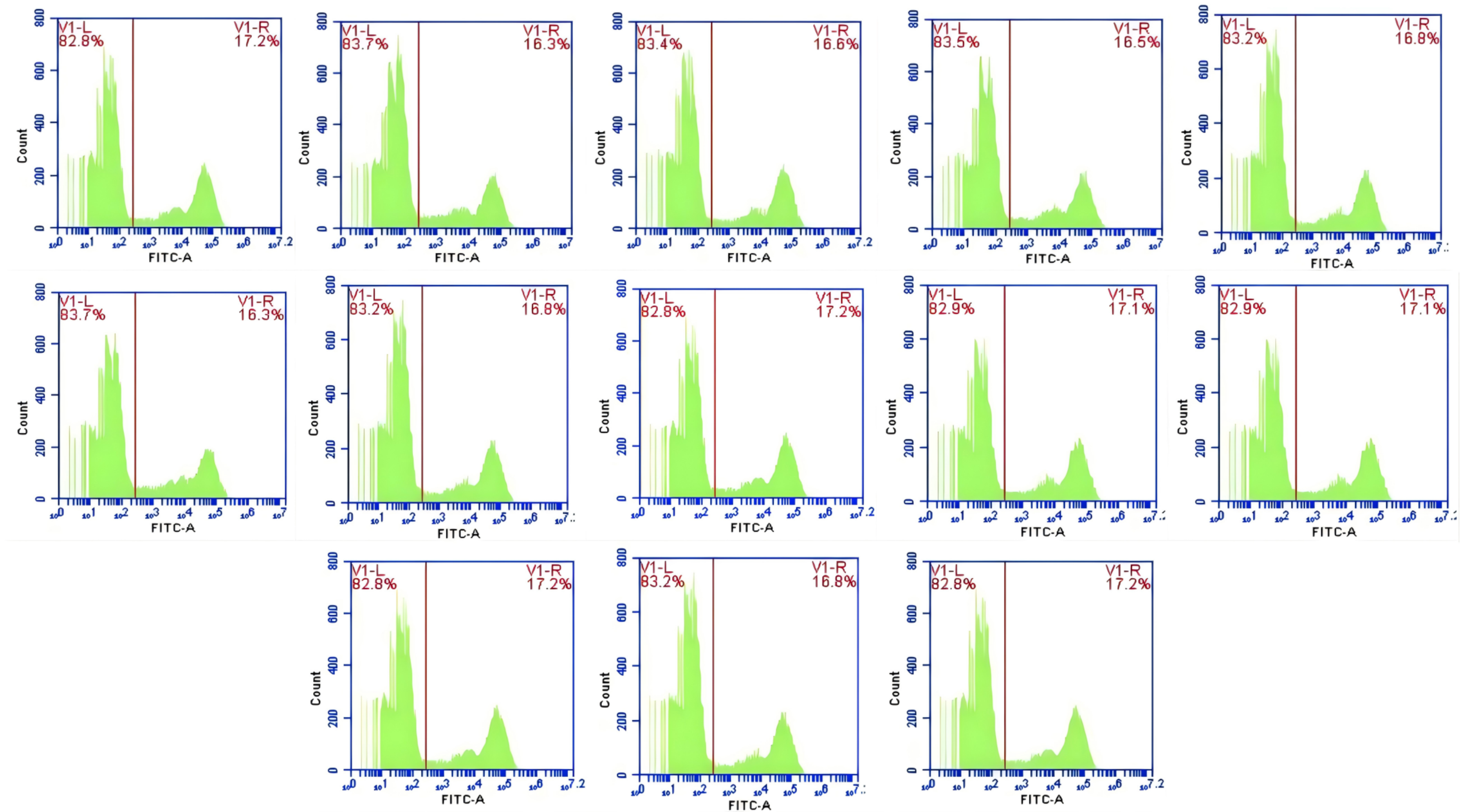

Supplement: Supplemental Information 2 [file peerj-13-18934-s002.zip › Picture supplement/Figure 3/Figure 3.pdf]

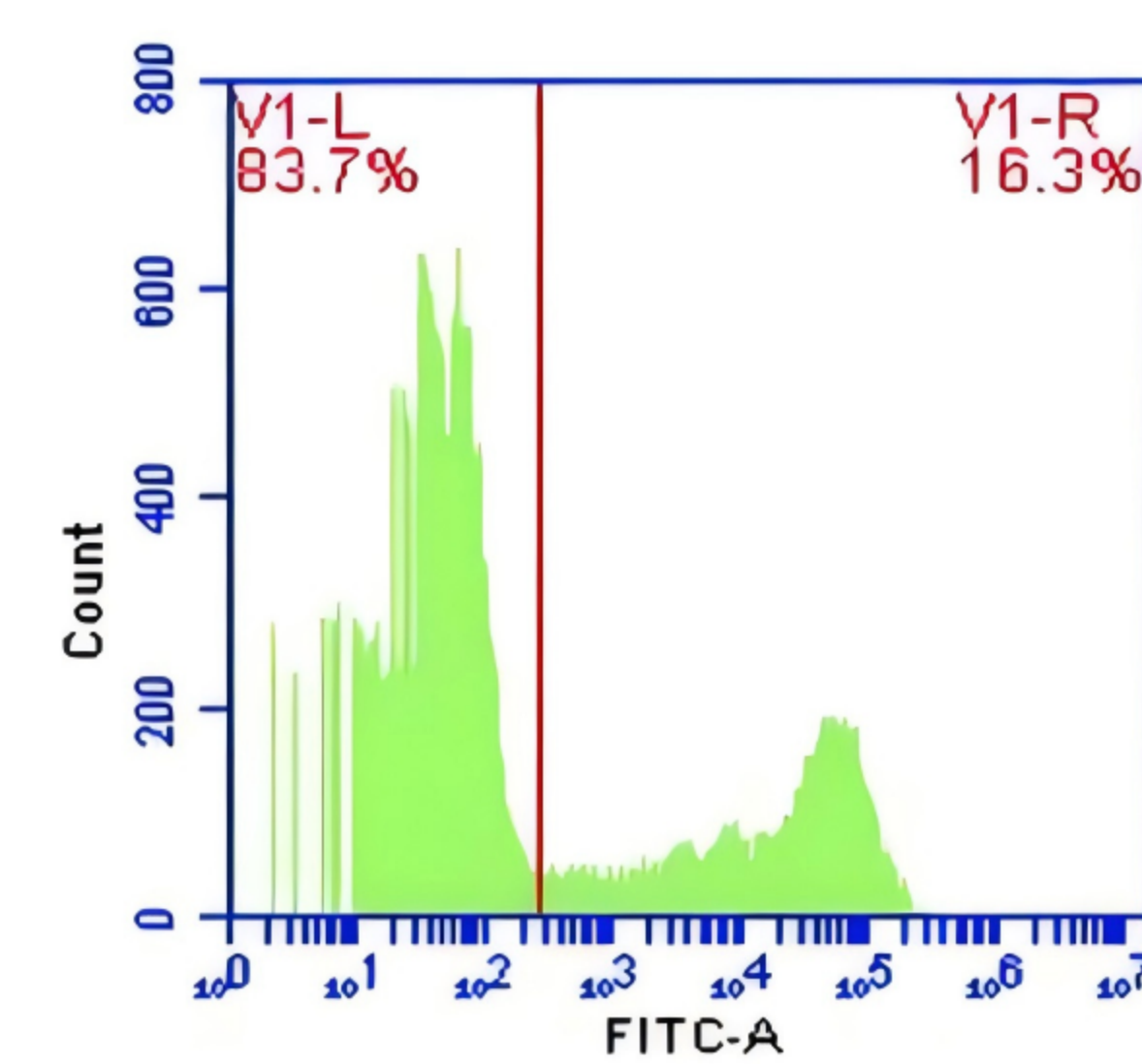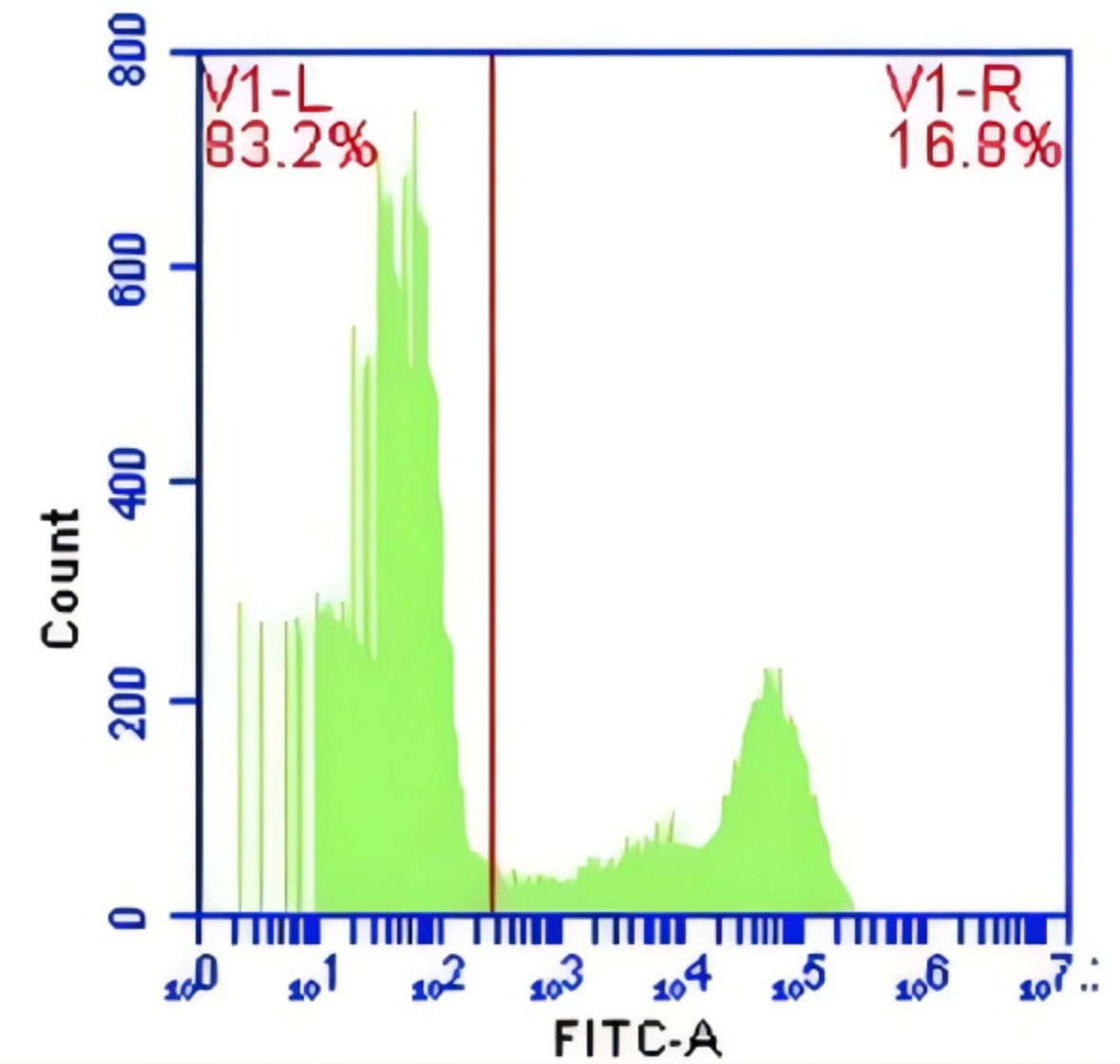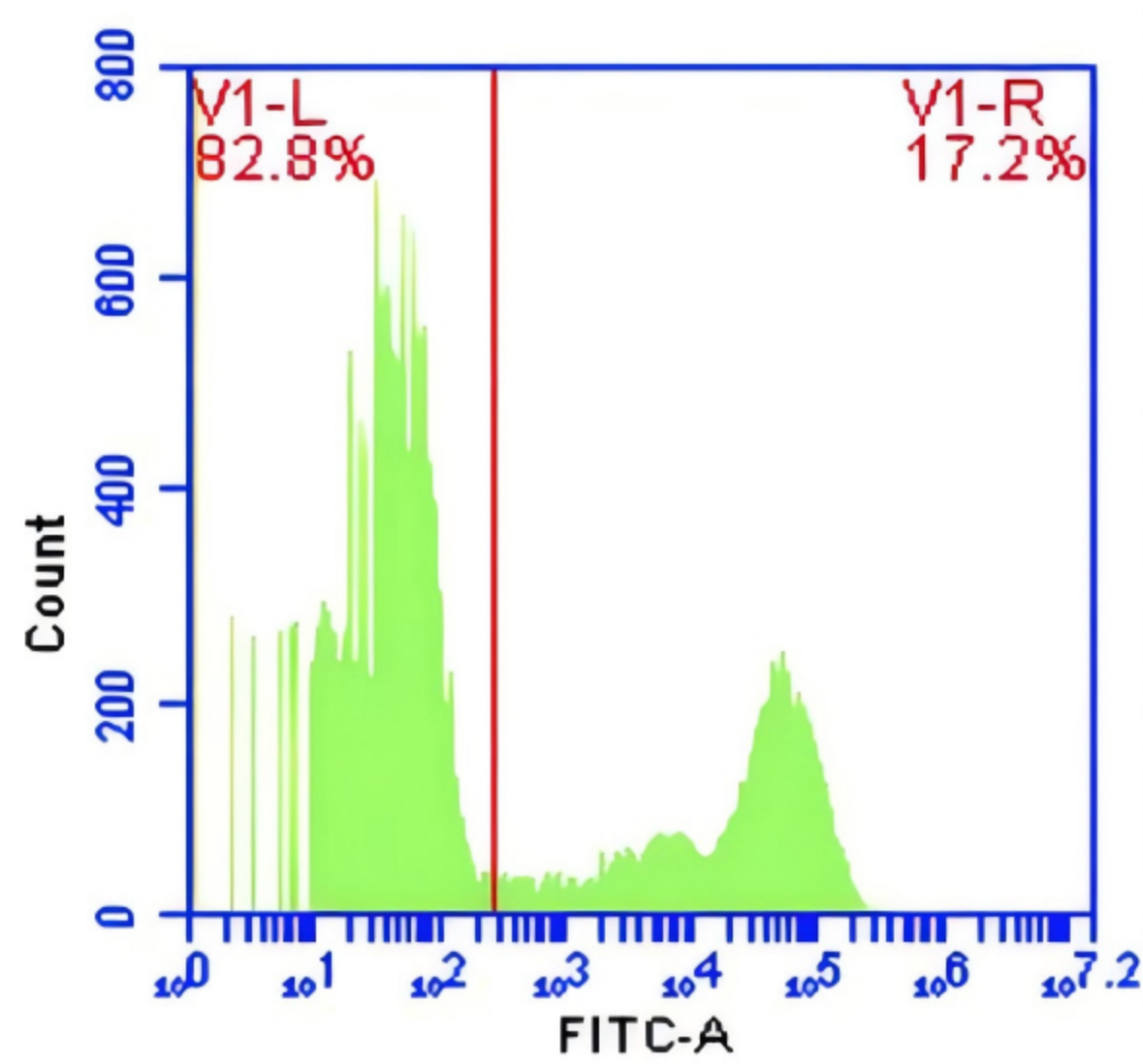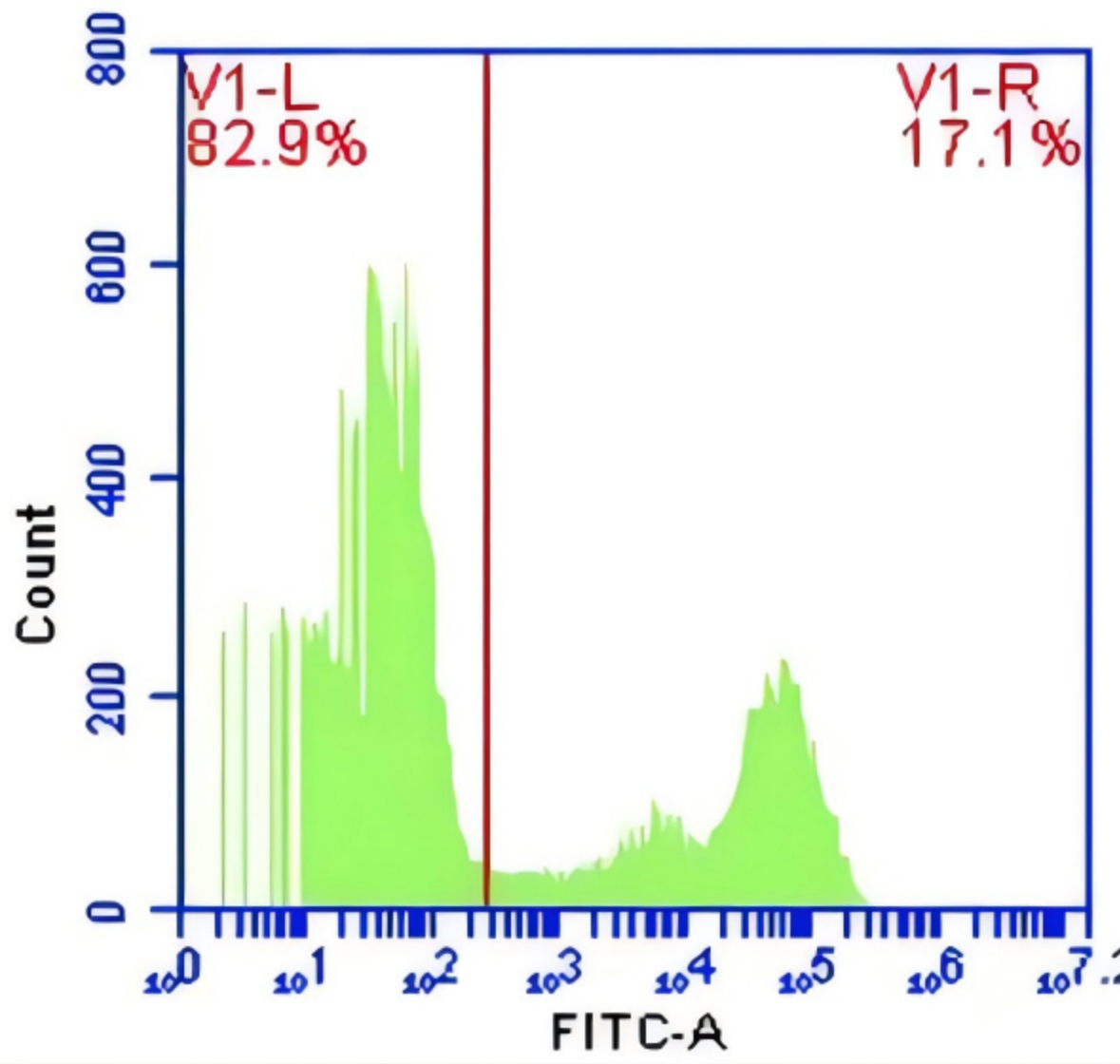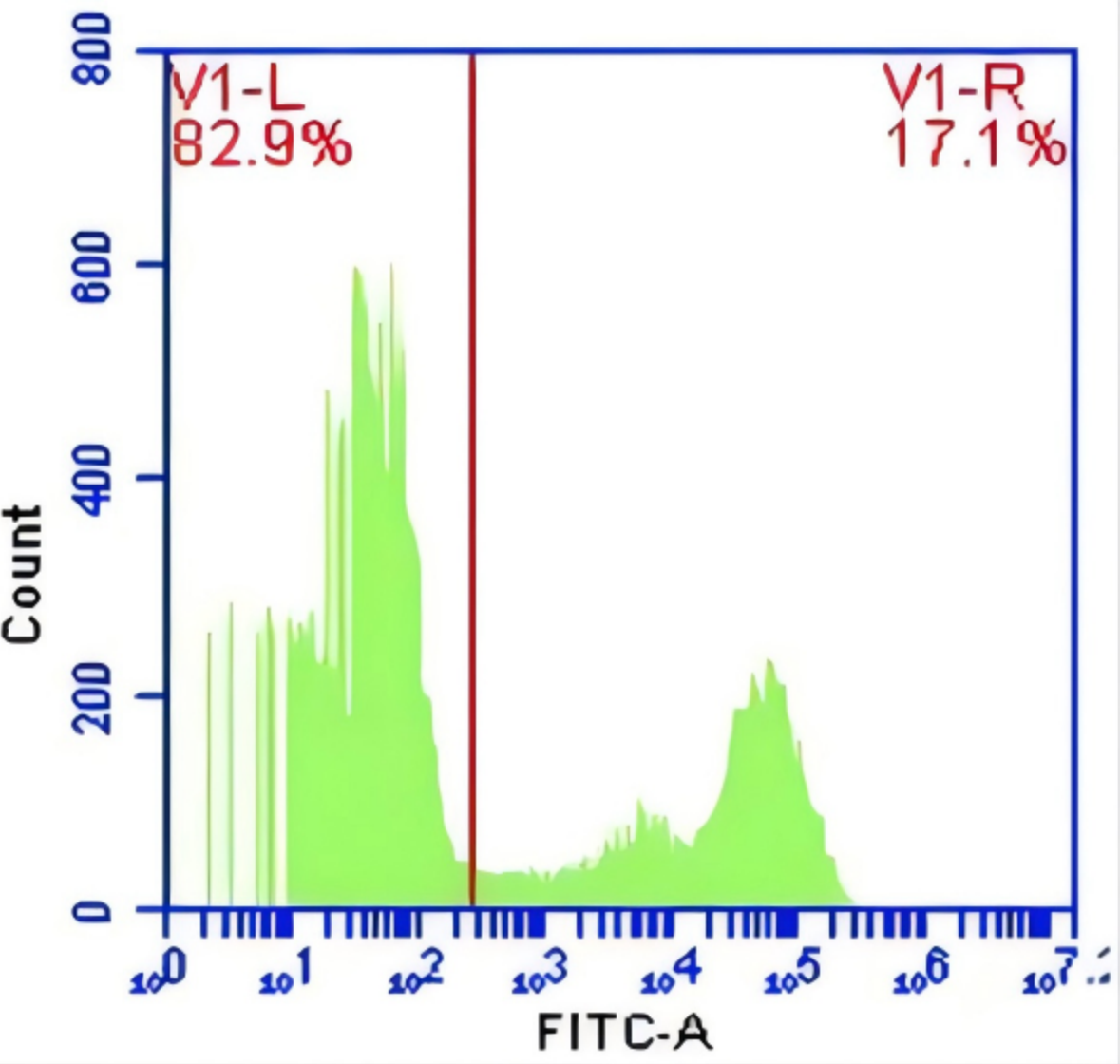

Supplement: Supplemental Information 2 [file peerj-13-18934-s002.zip › Picture supplement/Figure 3/Figure 3A.pdf]

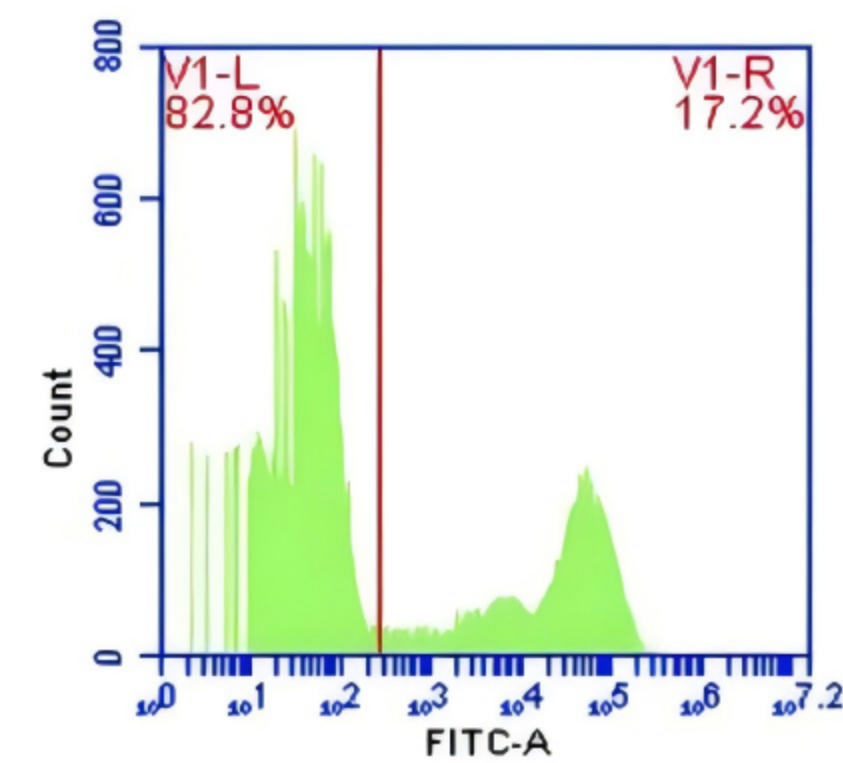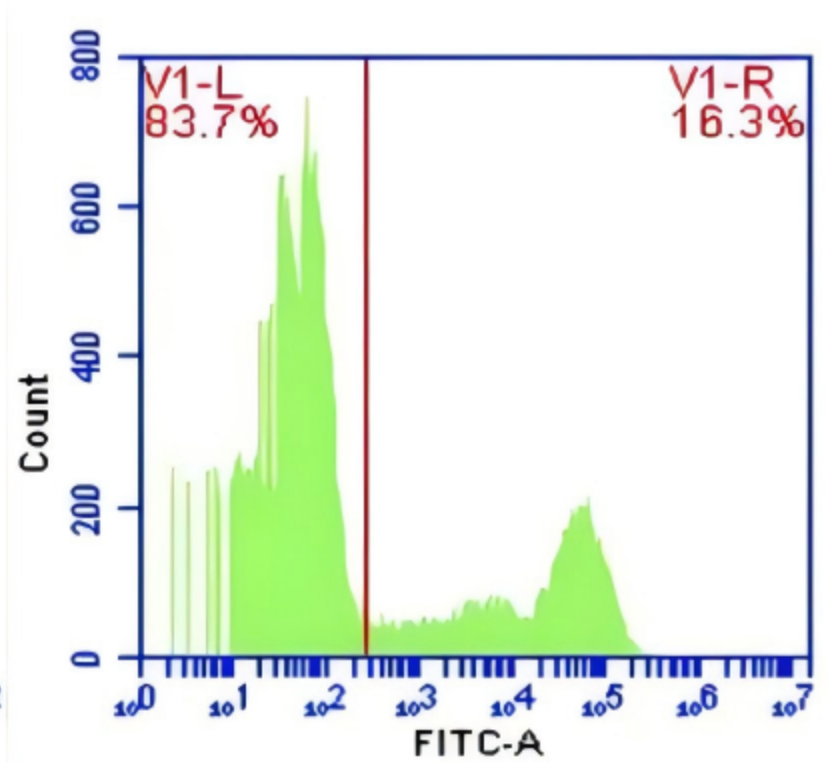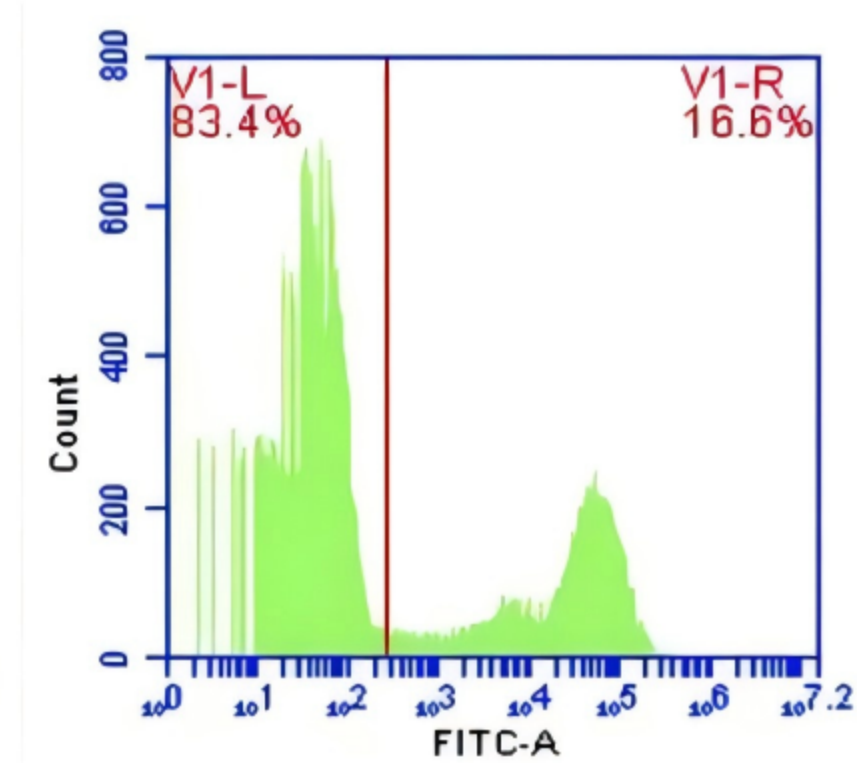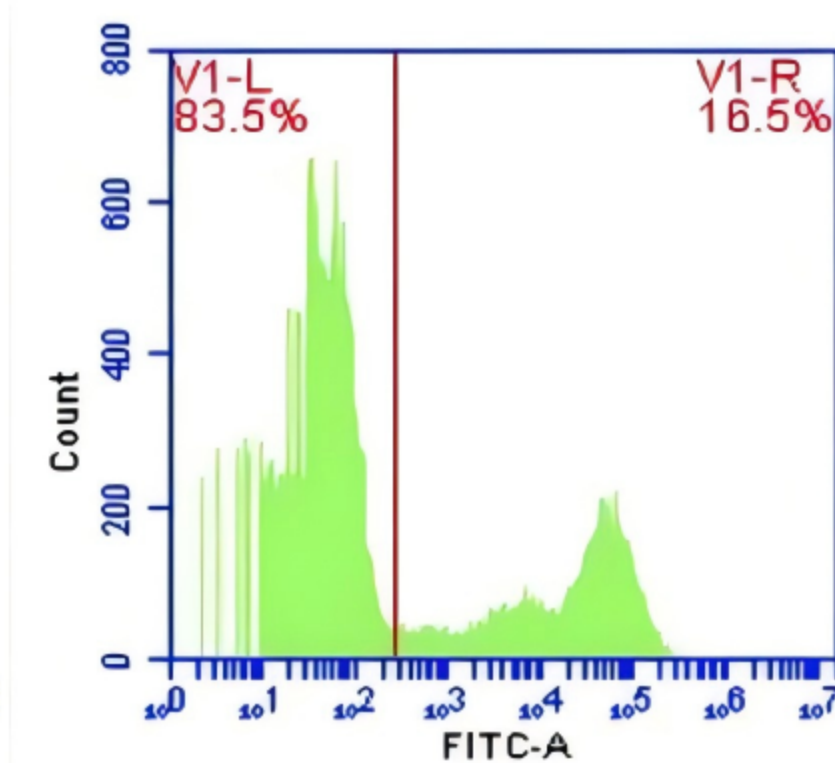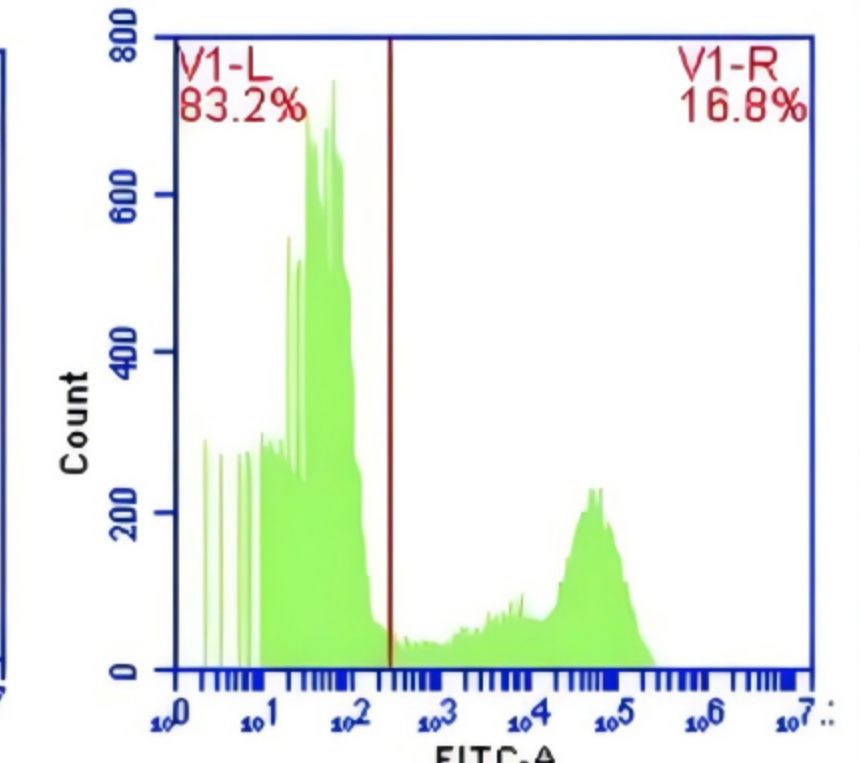

Supplement: Supplemental Information 2 [file peerj-13-18934-s002.zip › Picture supplement/Figure 3/Figure 3B.pdf]

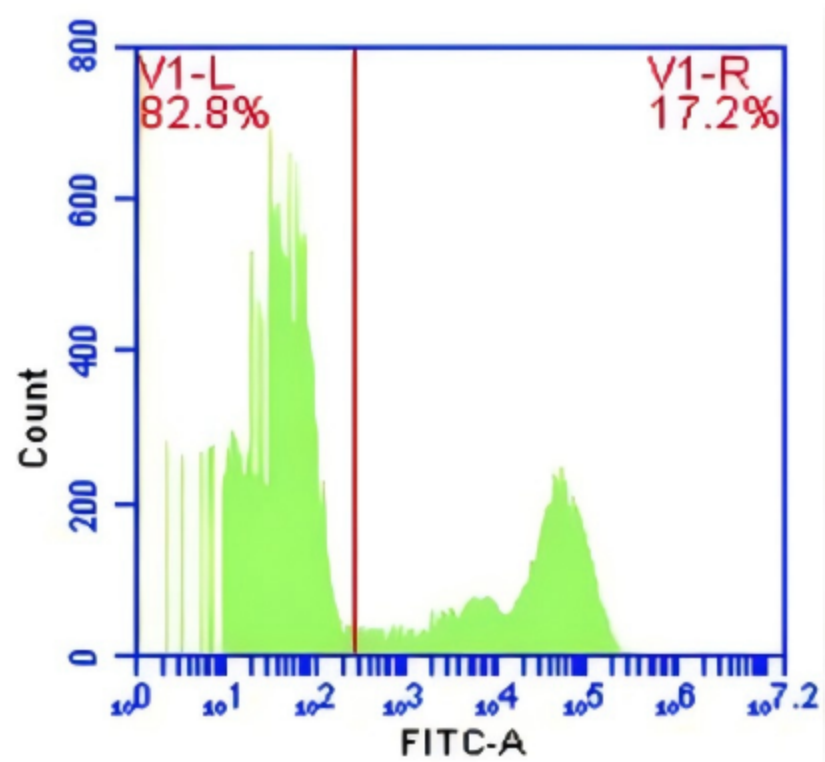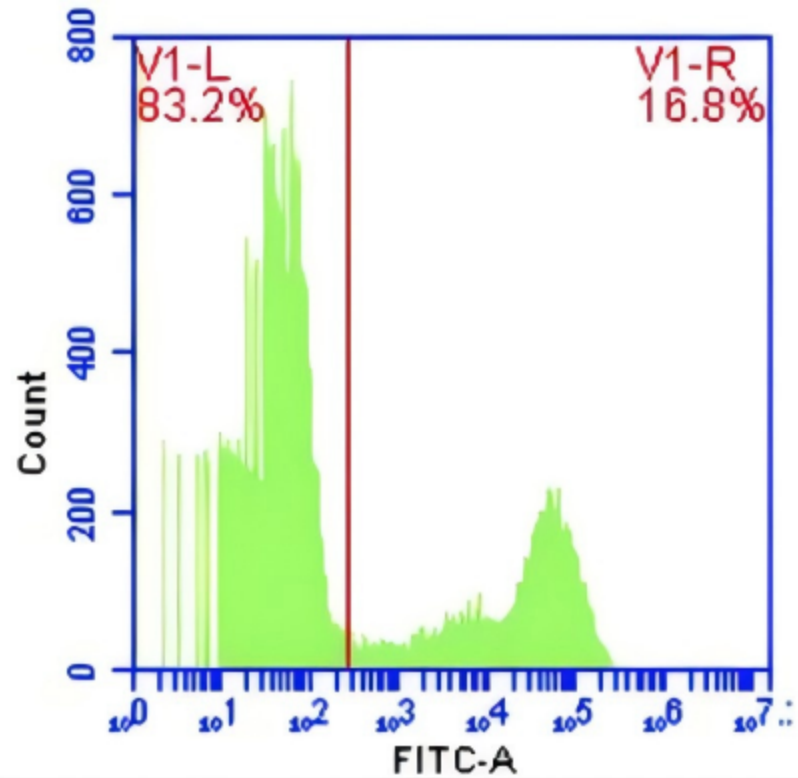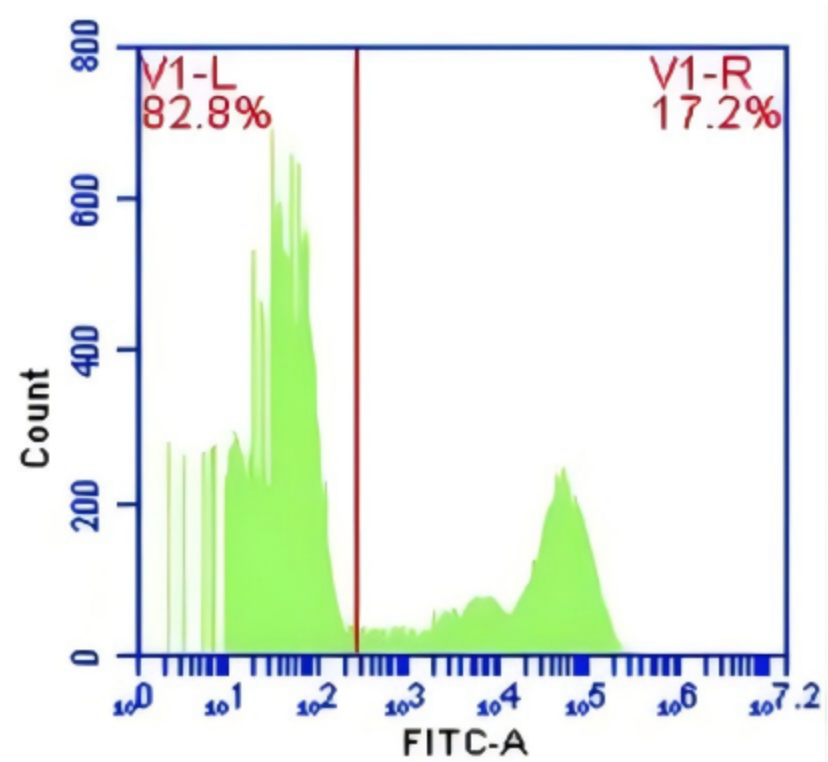

Supplement: Supplemental Information 2 [file peerj-13-18934-s002.zip › Picture supplement/Figure 3/Figure 3C.pdf]

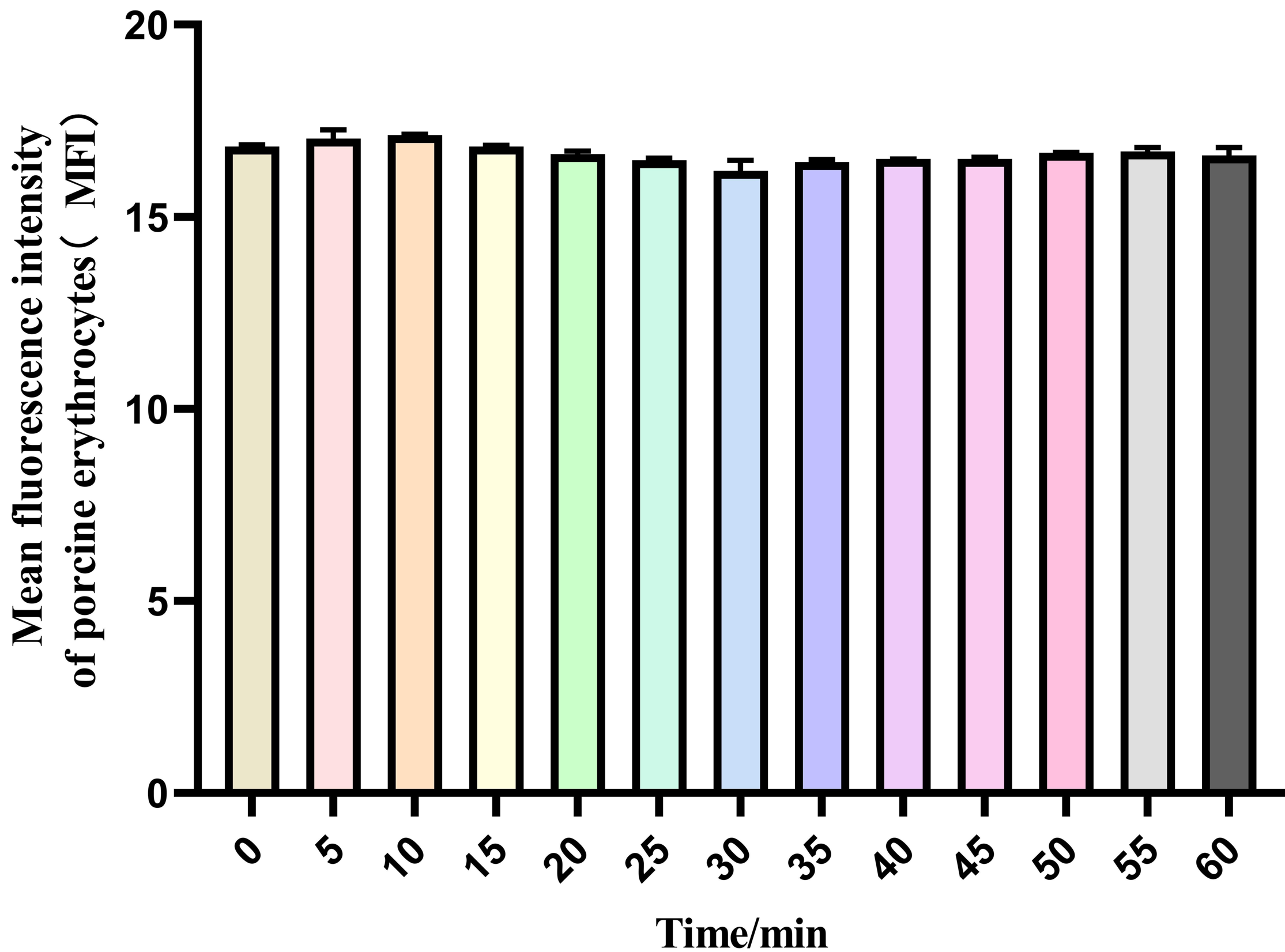

Supplement: Supplemental Information 2 [file peerj-13-18934-s002.zip › Picture supplement/Figure 4/Figure 4.pdf]

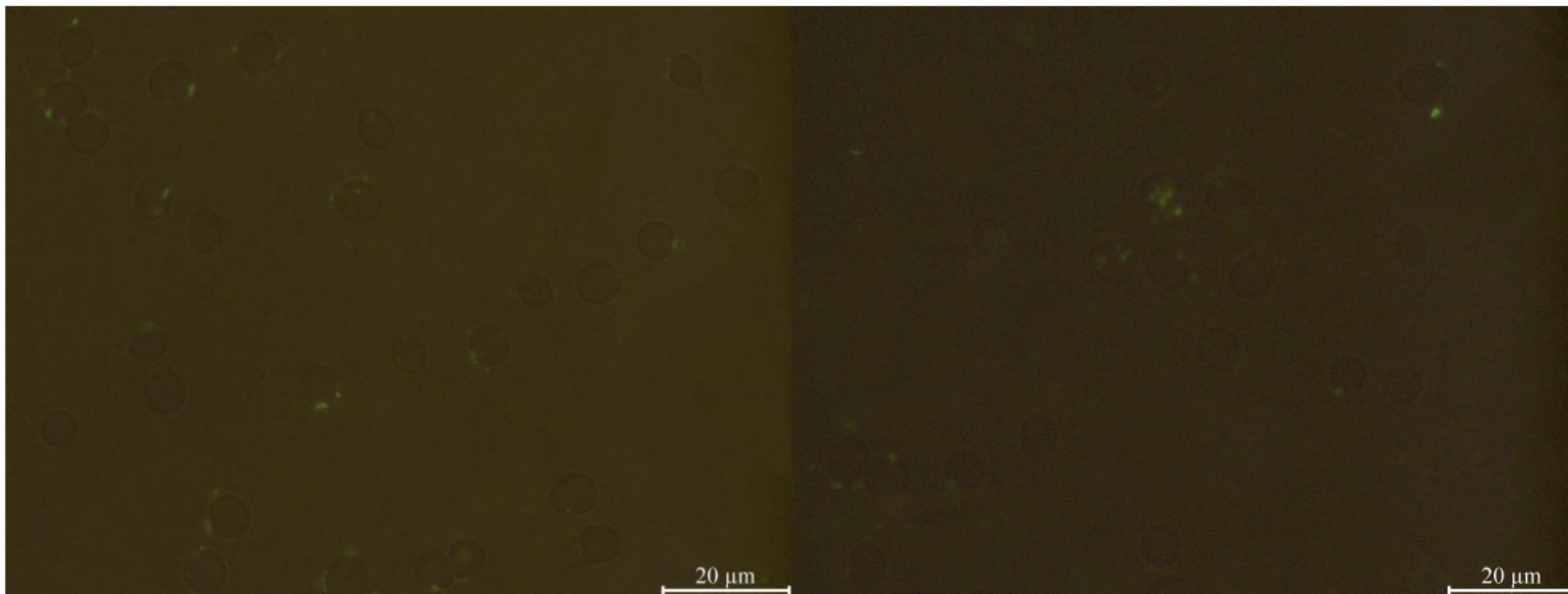

Supplement: Supplemental Information 2 [file peerj-13-18934-s002.zip › Picture supplement/Figure 5/Figure 5.pdf]

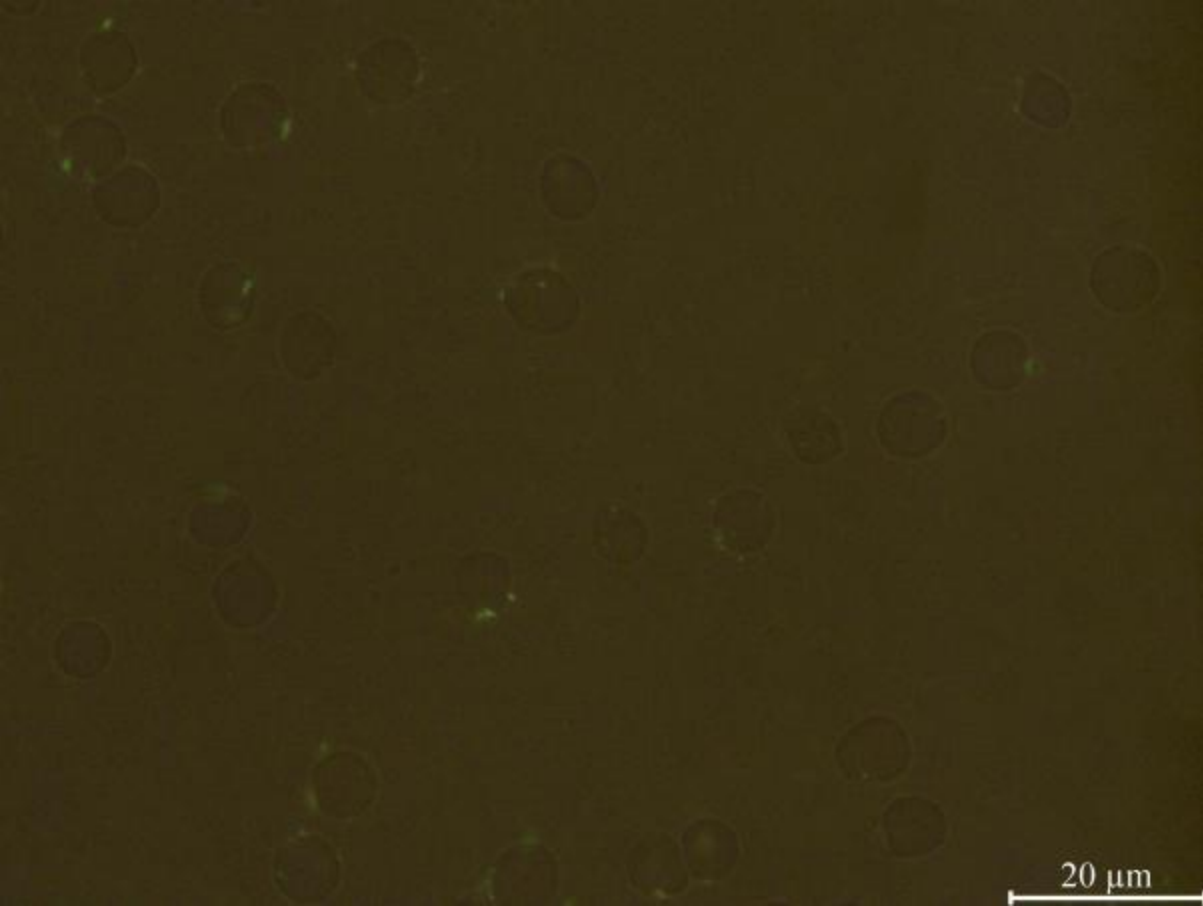

Supplement: Supplemental Information 2 [file peerj-13-18934-s002.zip › Picture supplement/Figure 5/Figure 5A-1.pdf]

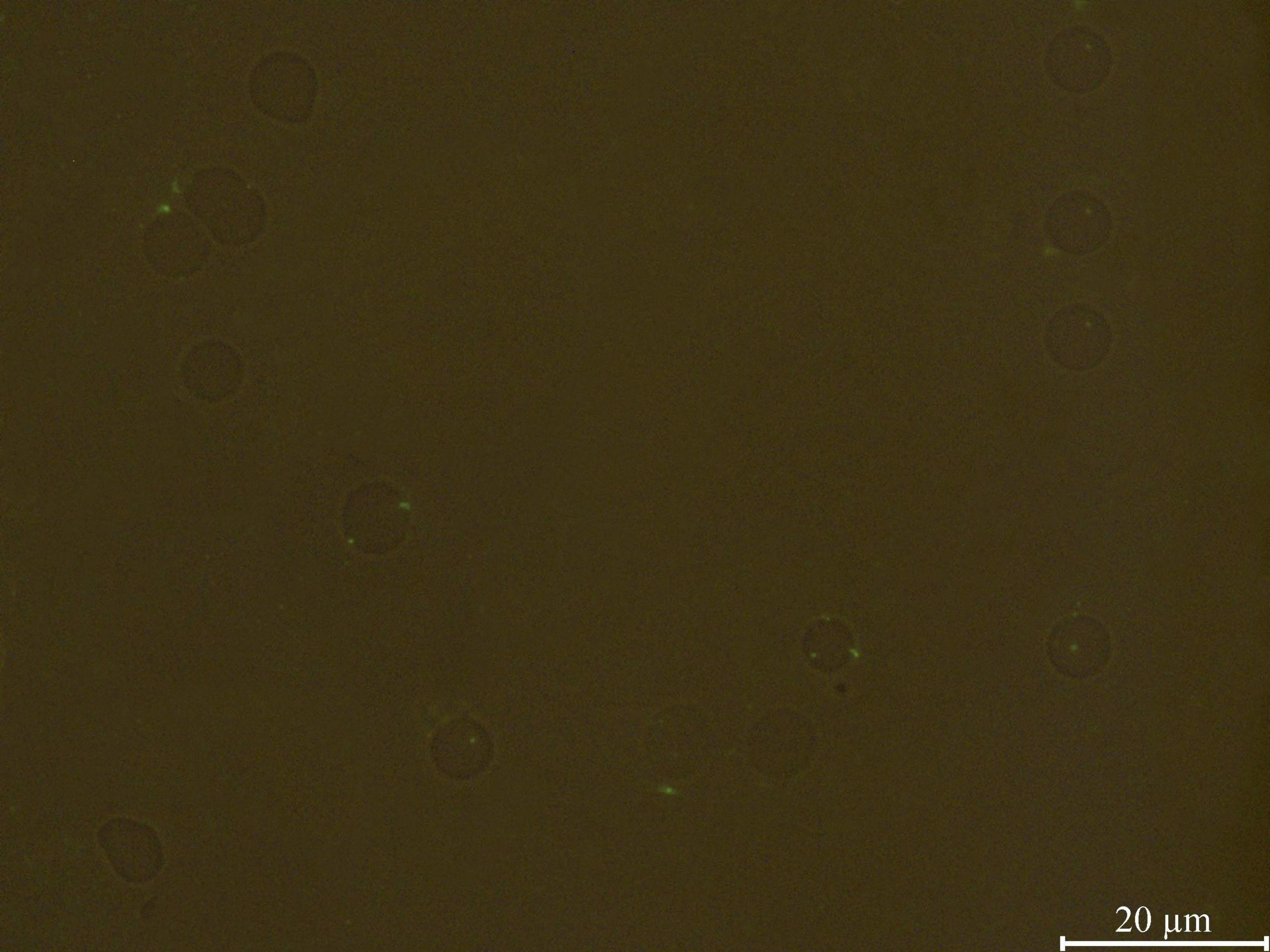

20 μm

Supplement: Supplemental Information 2 [file peerj-13-18934-s002.zip › Picture supplement/Figure 5/Figure 5A-2.pdf]

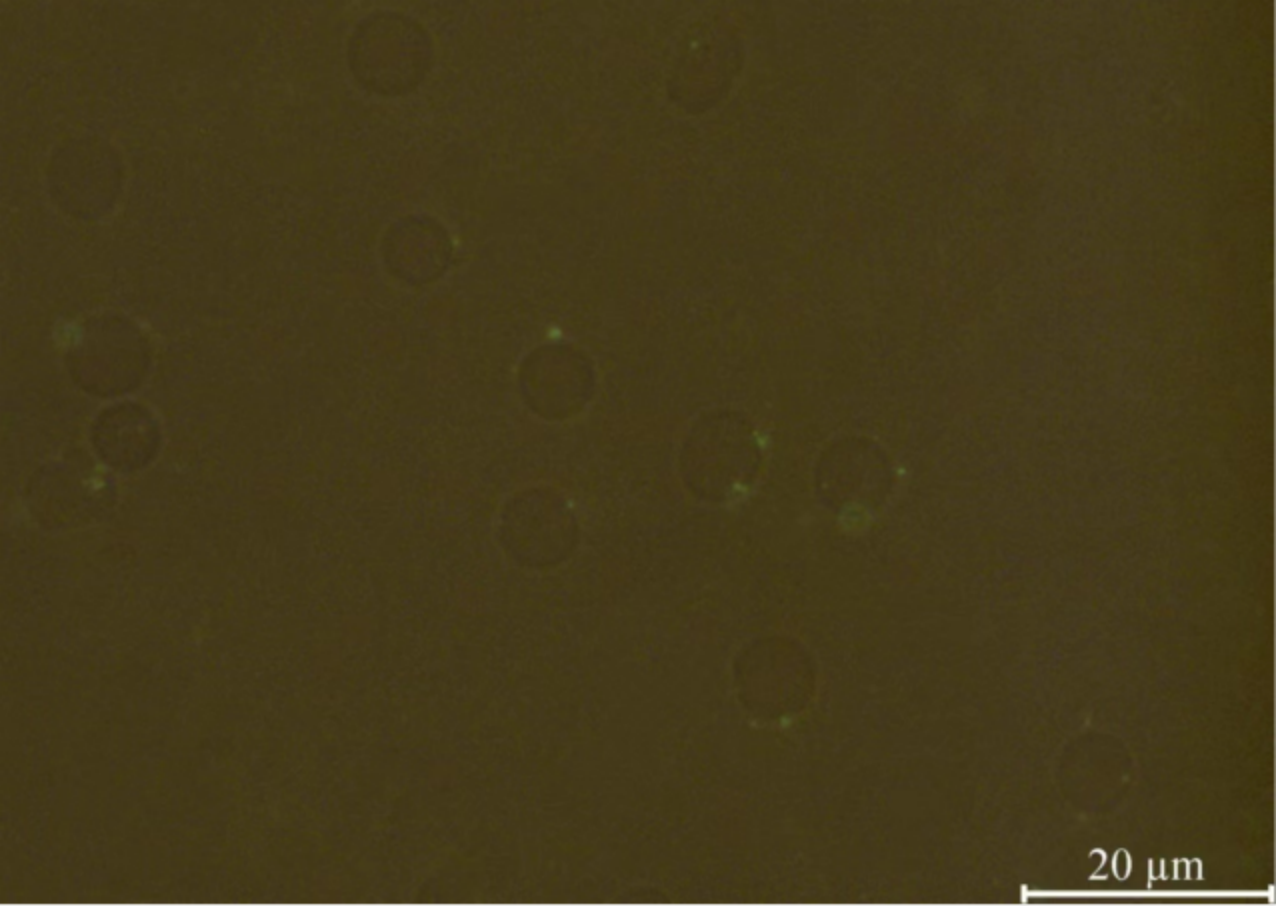

20  $\mu\text{m}$

Supplement: Supplemental Information 2 [file peerj-13-18934-s002.zip › Picture supplement/Figure 5/Figure 5B-1.pdf]

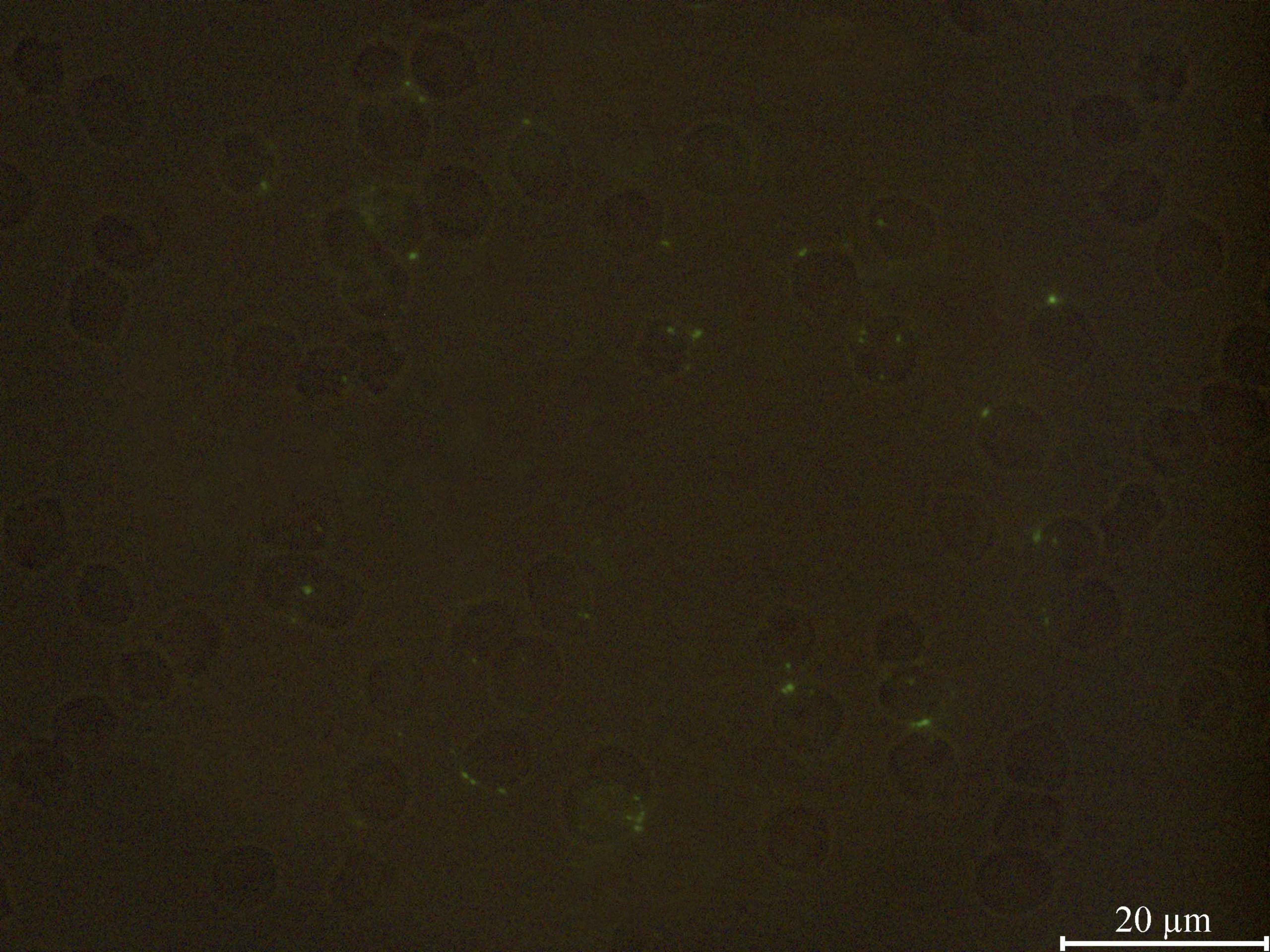

20  $\mu\text{m}$

Supplement: Supplemental Information 2 [file peerj-13-18934-s002.zip › Picture supplement/Figure 5/Figure 5B-2.pdf]

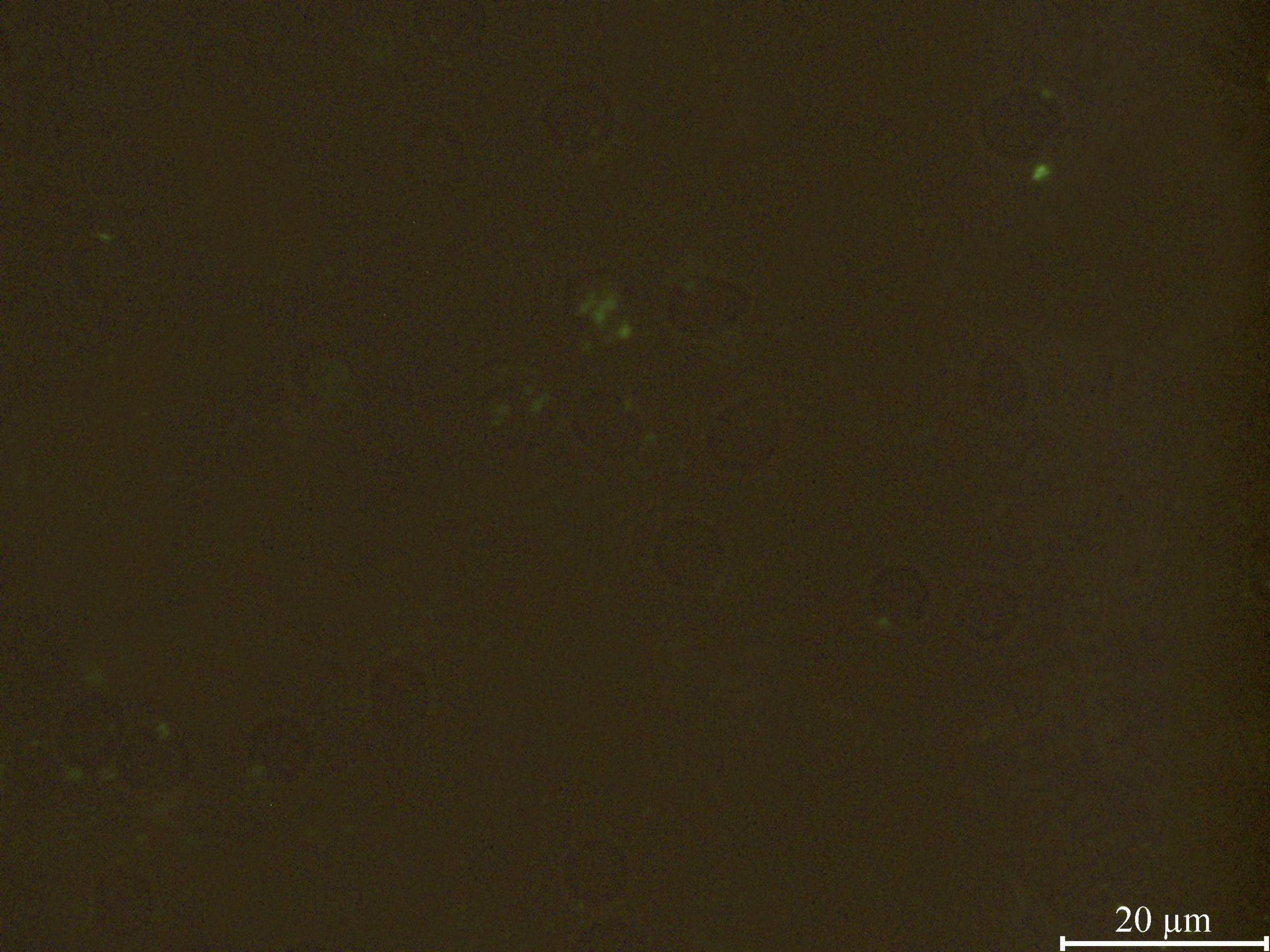

20 μm

Supplement: Supplemental Information 2 [file peerj-13-18934-s002.zip › Picture supplement/Figure 5/Figure 5B-3.pdf]

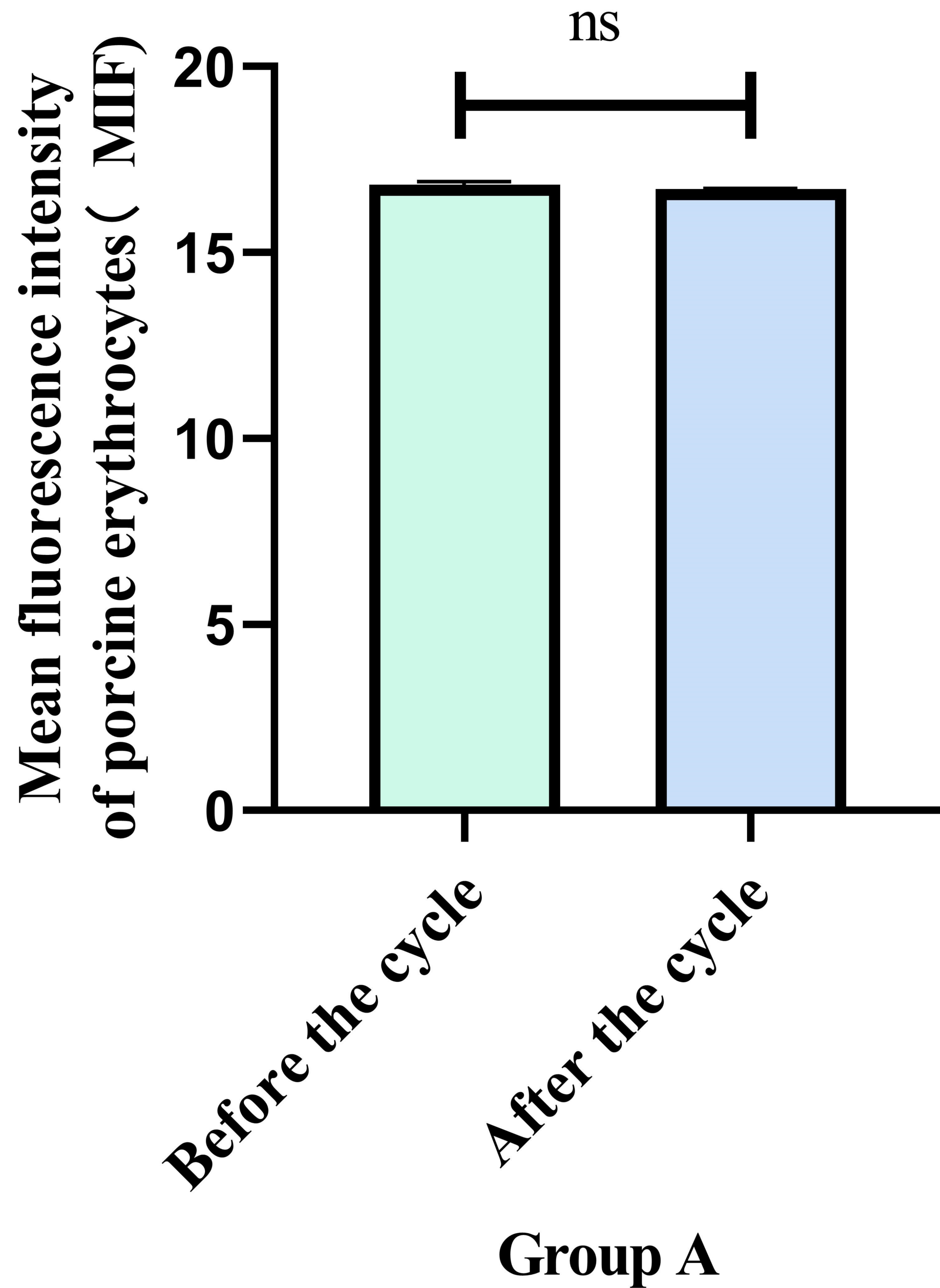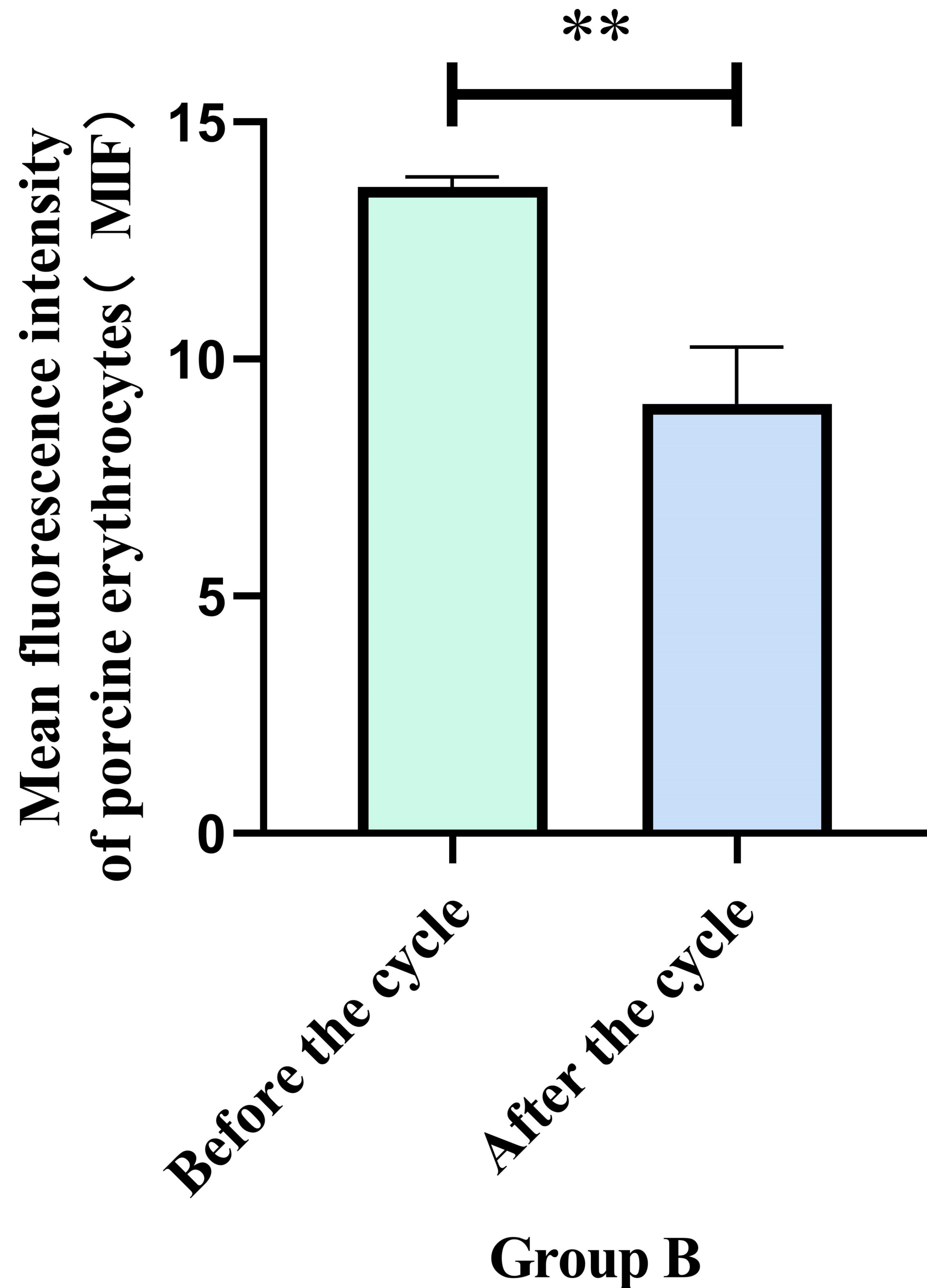

Supplement: Supplemental Information 2 [file peerj-13-18934-s002.zip › Picture supplement/Figure 6/Figure 6.pdf]

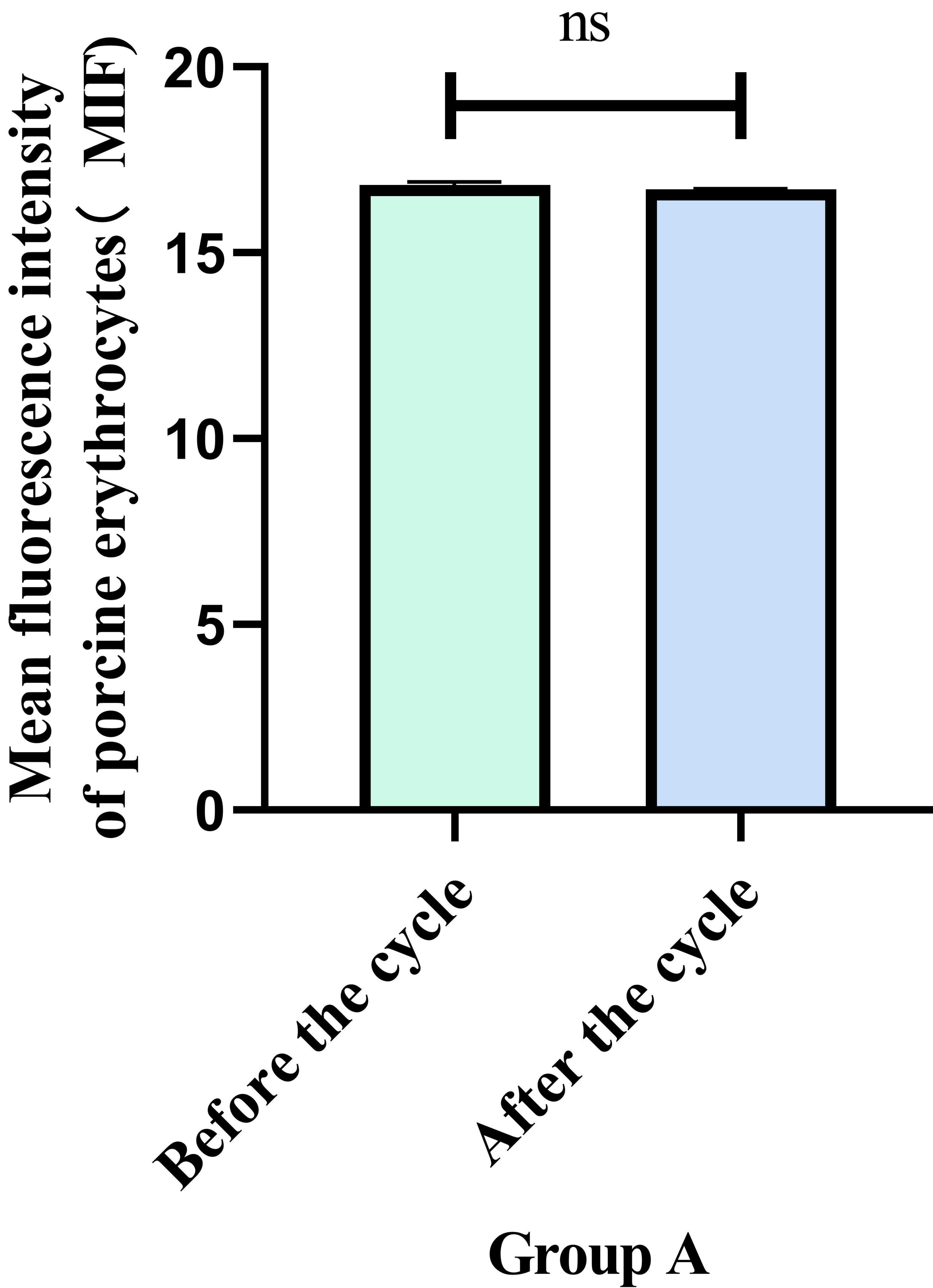

Supplement: Supplemental Information 2 [file peerj-13-18934-s002.zip › Picture supplement/Figure 6/Figure 6A.pdf]

**Mean fluorescence intensity  
of porcine erythrocytes ( MIF)**

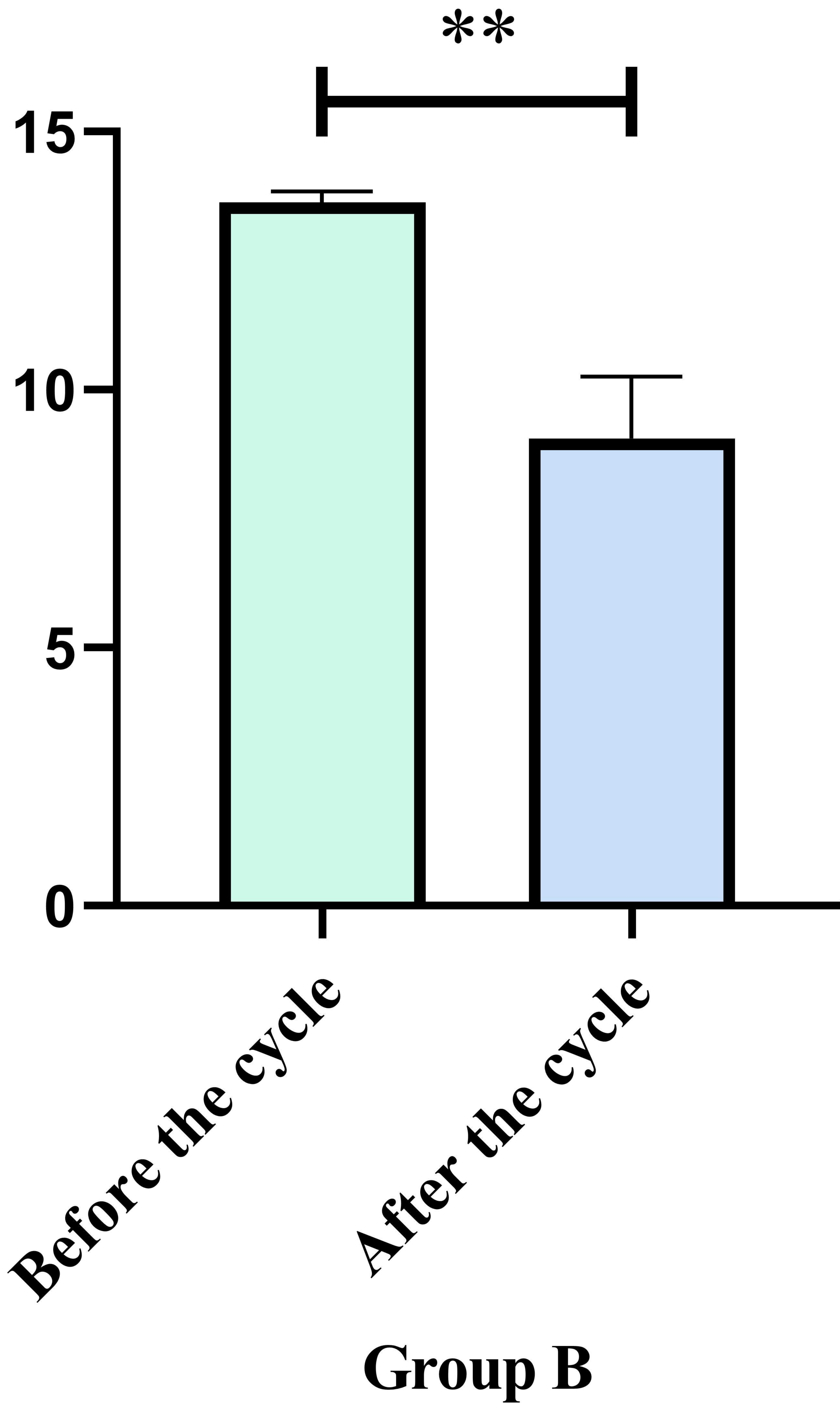

Supplement: Supplemental Information 2 [file peerj-13-18934-s002.zip › Picture supplement/Figure 6/Figure 6B.pdf]

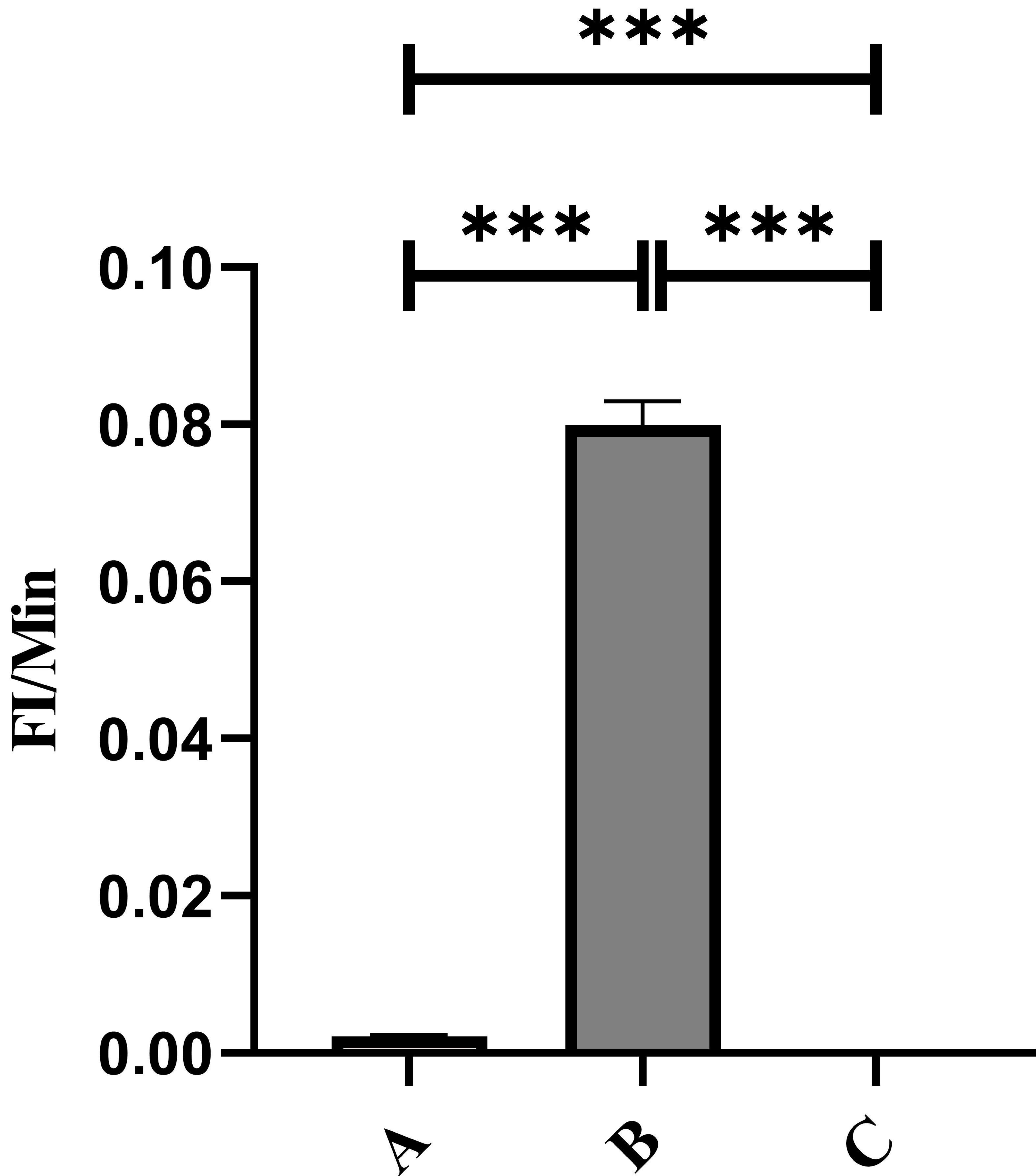

Supplement: Supplemental Information 2 [file peerj-13-18934-s002.zip › Picture supplement/Figure 7/Figure 7.pdf]

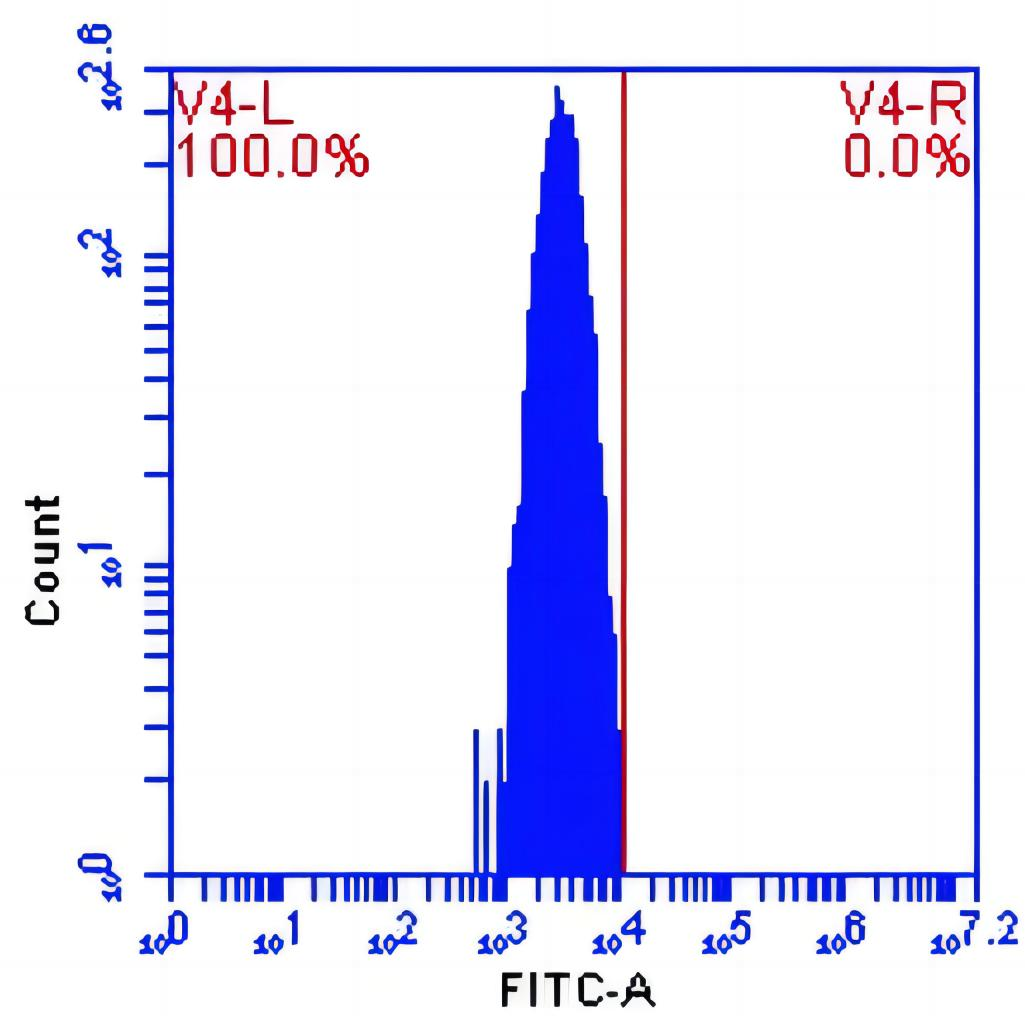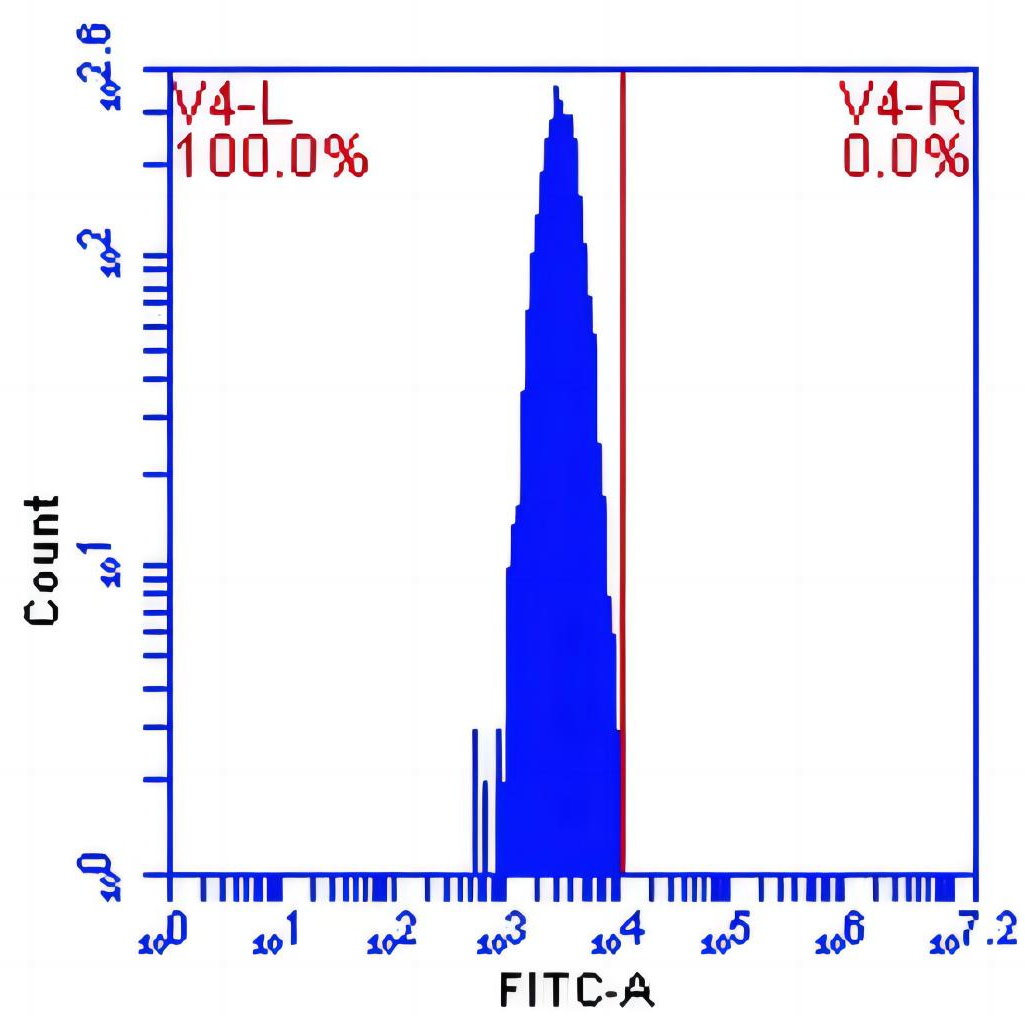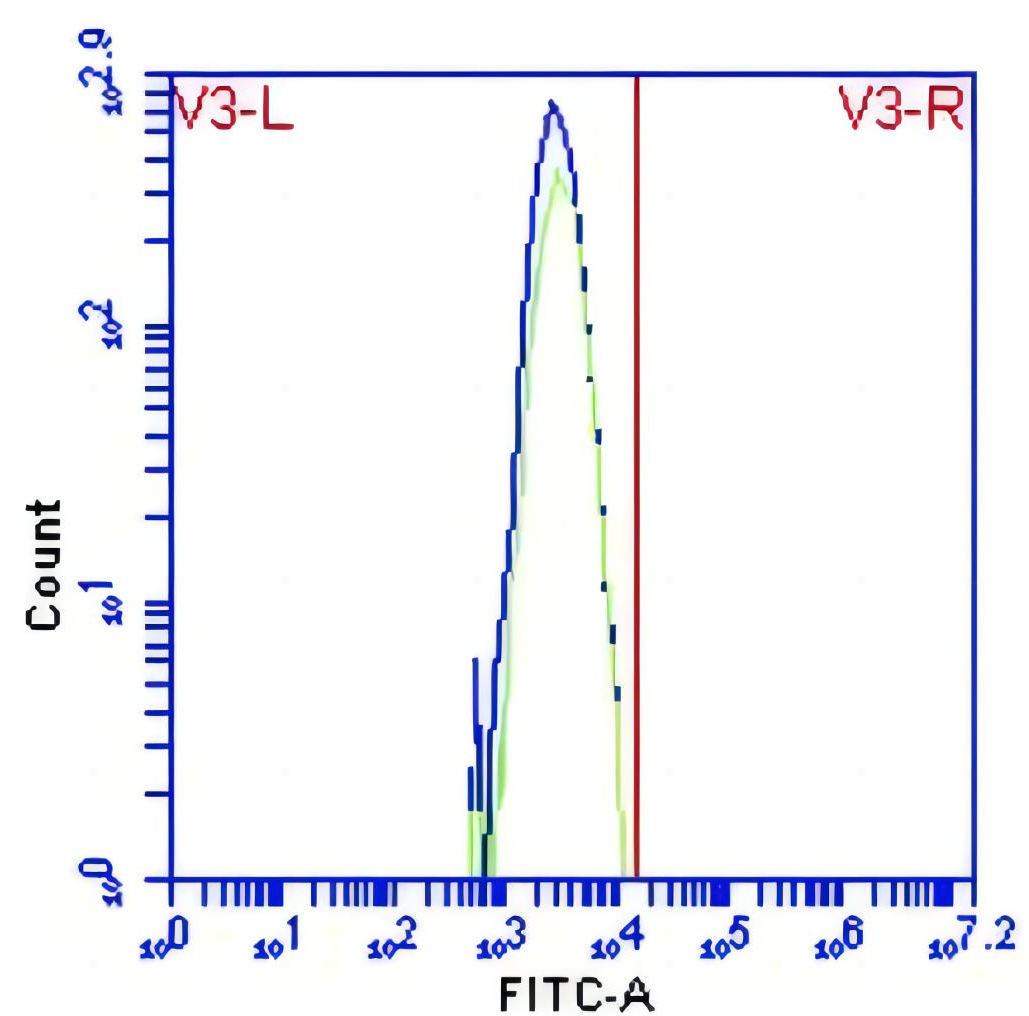

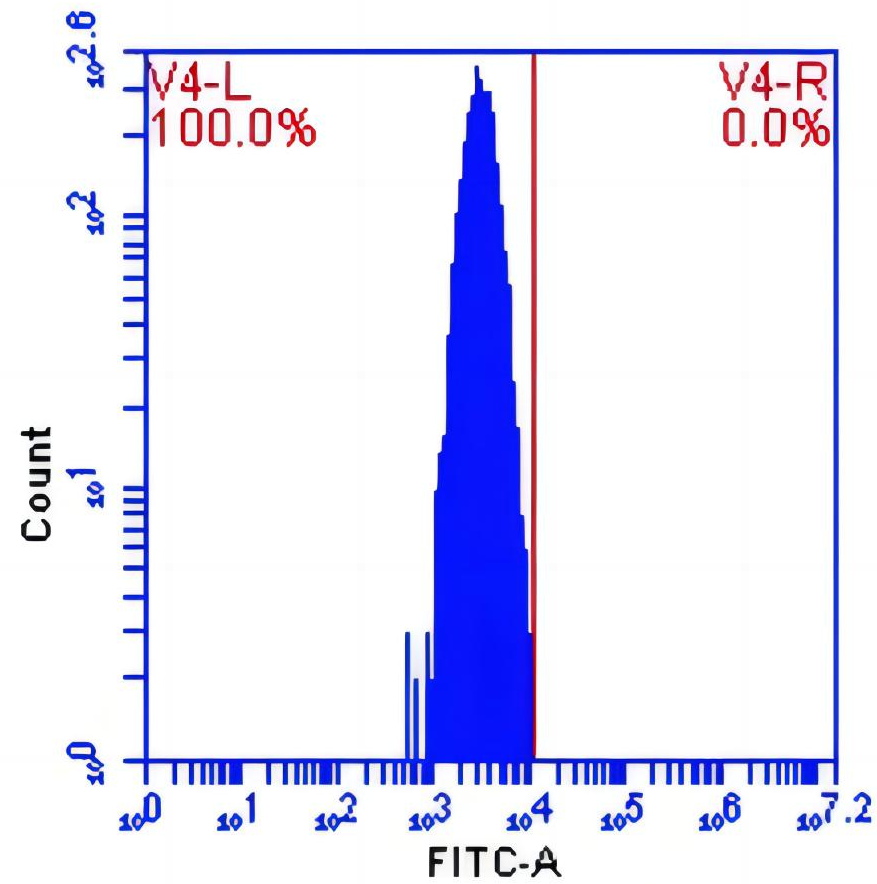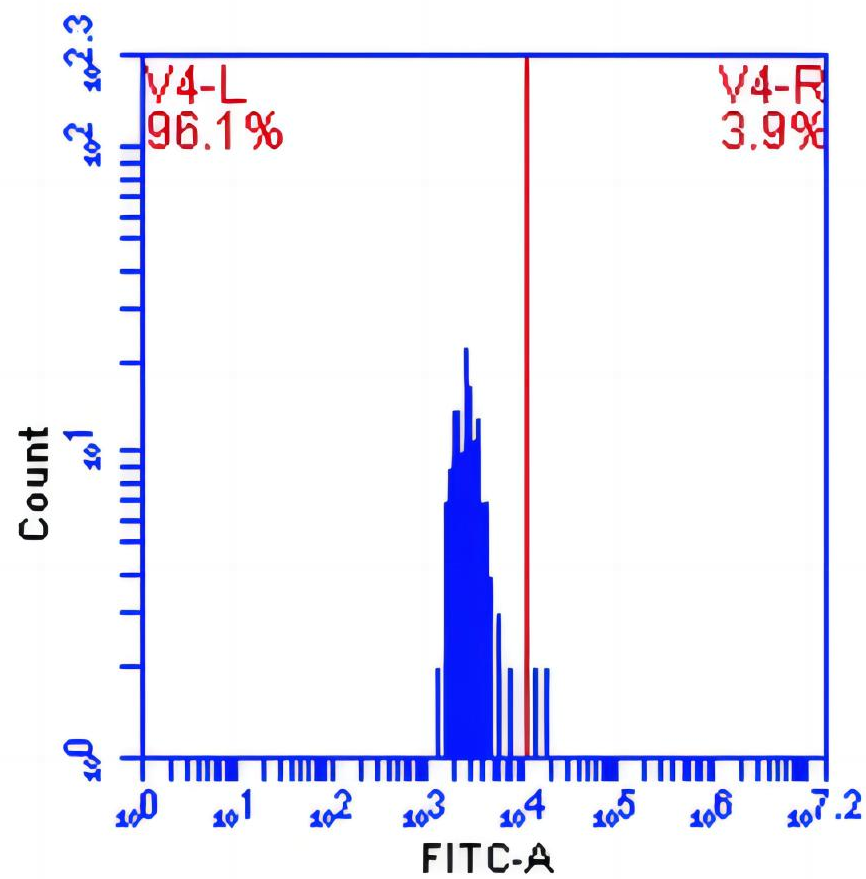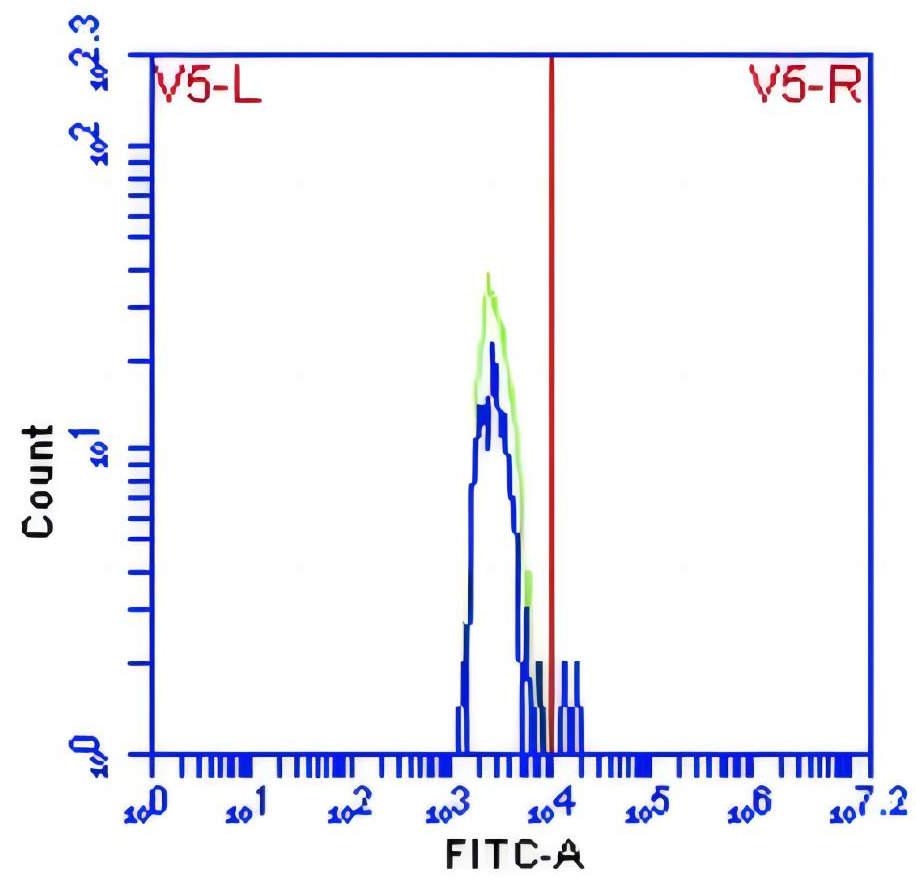

Supplement: Supplemental Information 2 [file peerj-13-18934-s002.zip › Picture supplement/Figure 8/Figure 8.pdf]

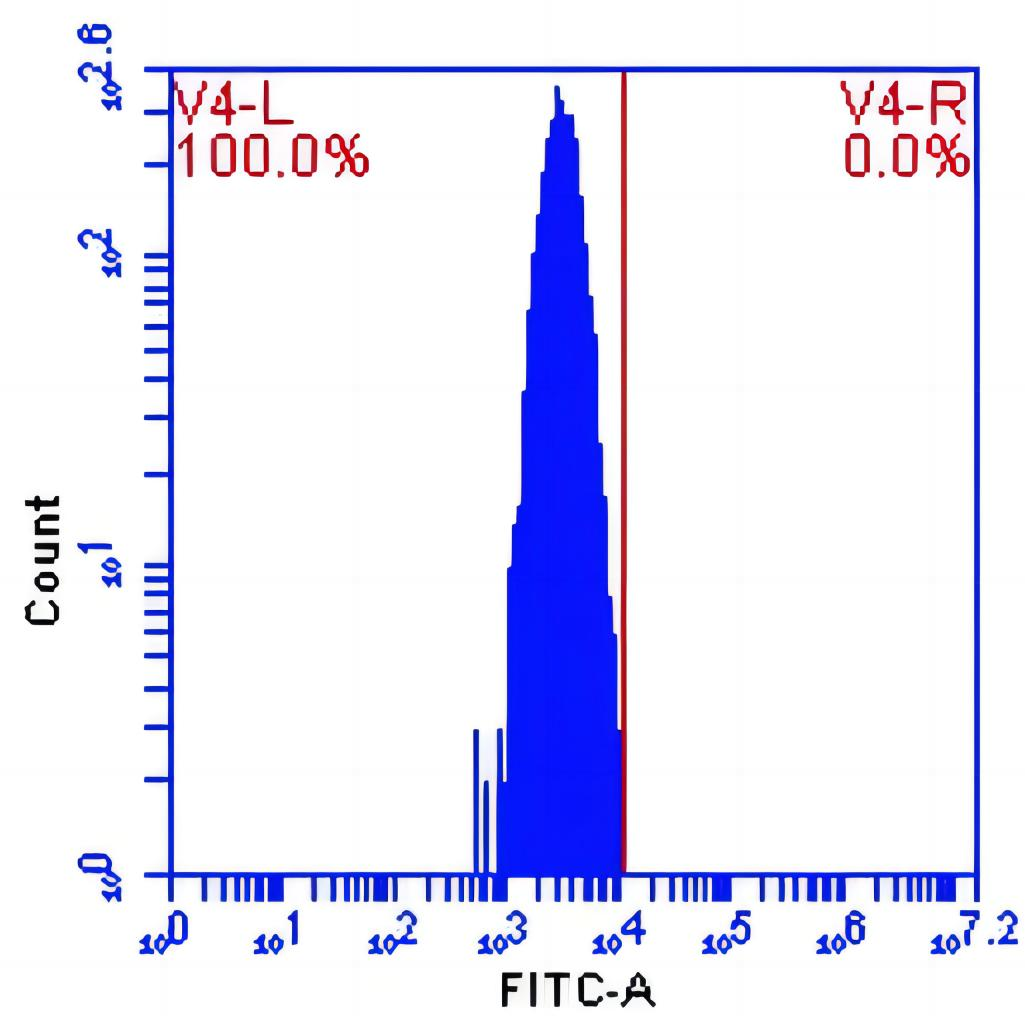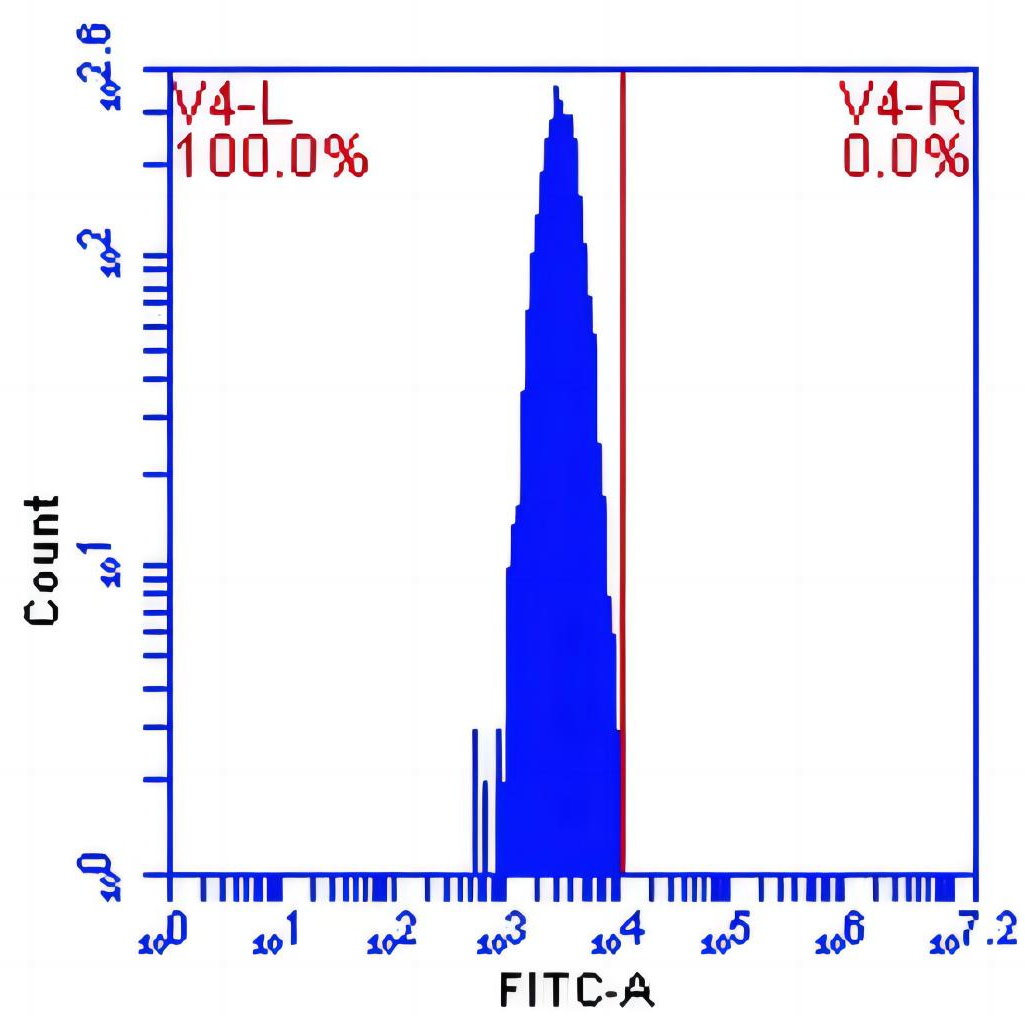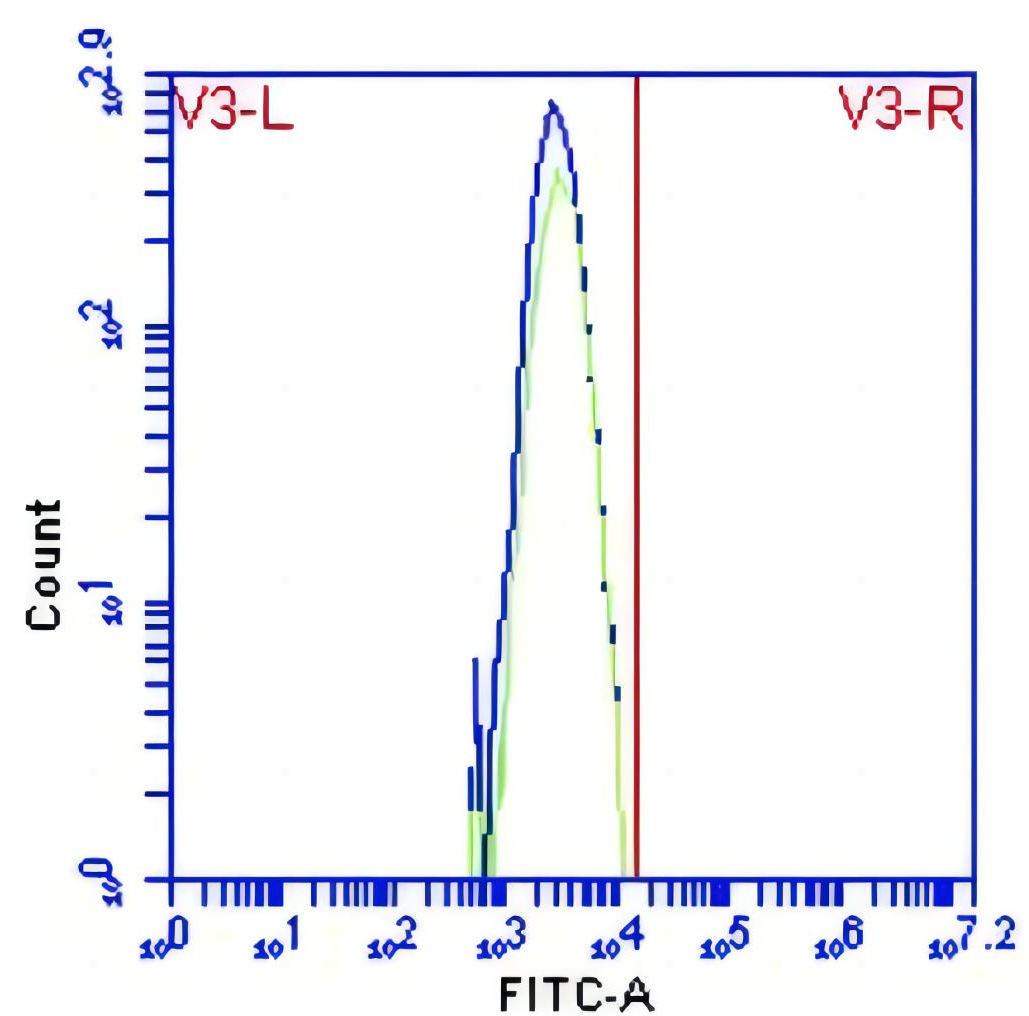

Supplement: Supplemental Information 2 [file peerj-13-18934-s002.zip › Picture supplement/Figure 8/Figure 8A.pdf]

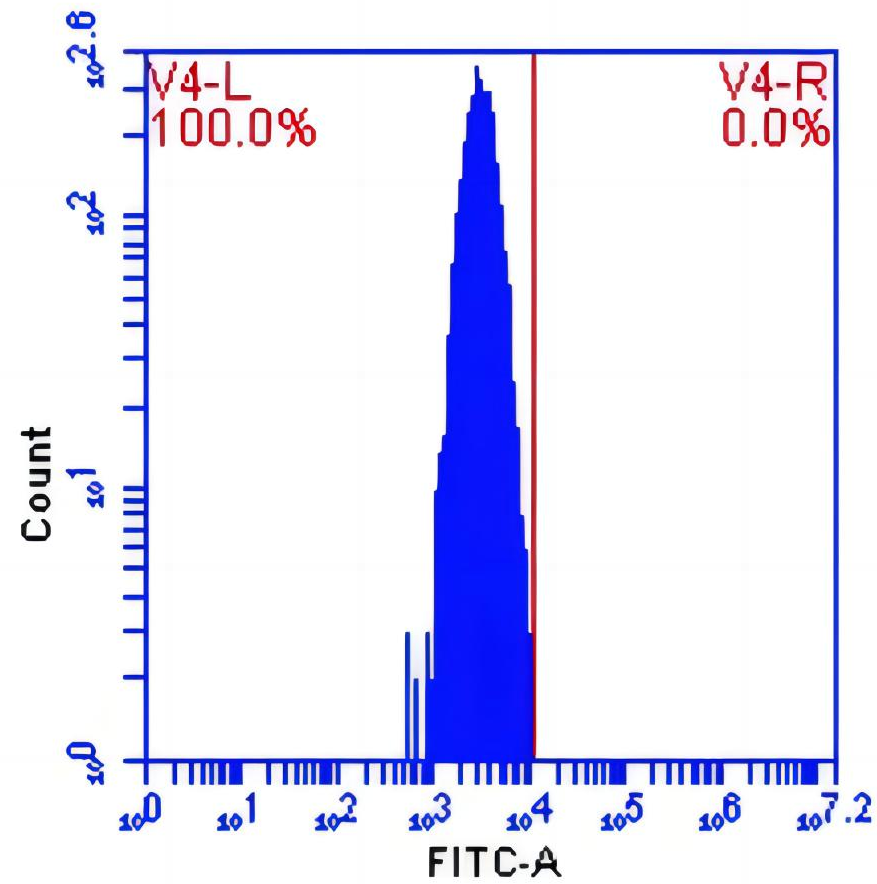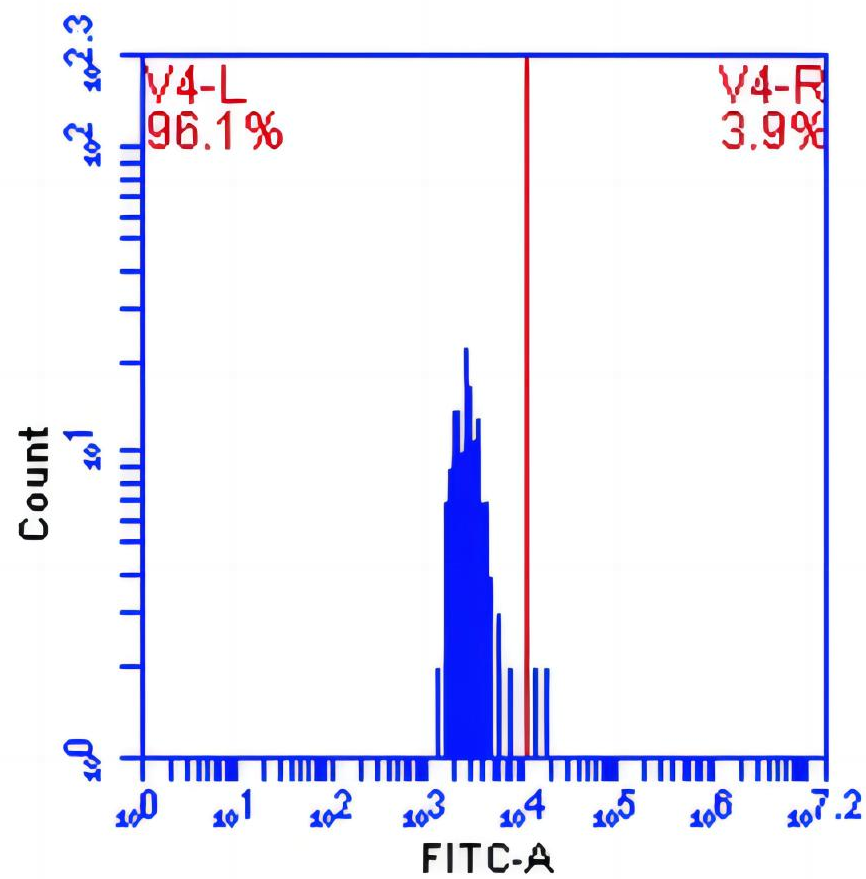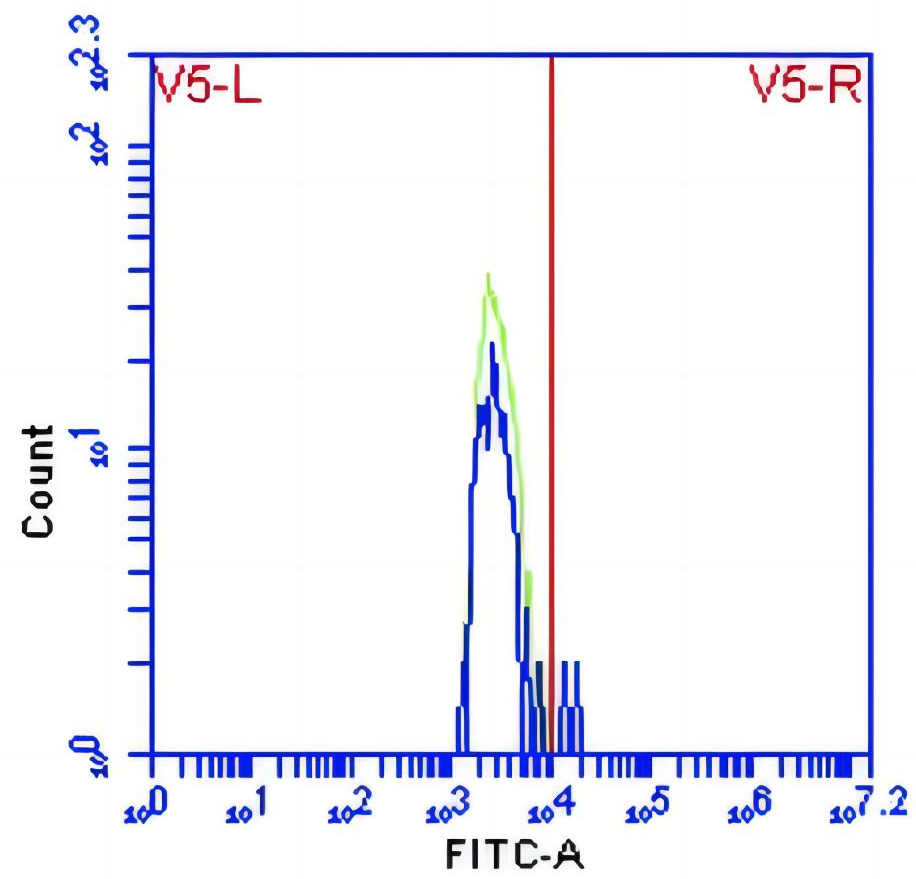

Supplement: Supplemental Information 2 [file peerj-13-18934-s002.zip › Picture supplement/Figure 8/Figure 8B.pdf]

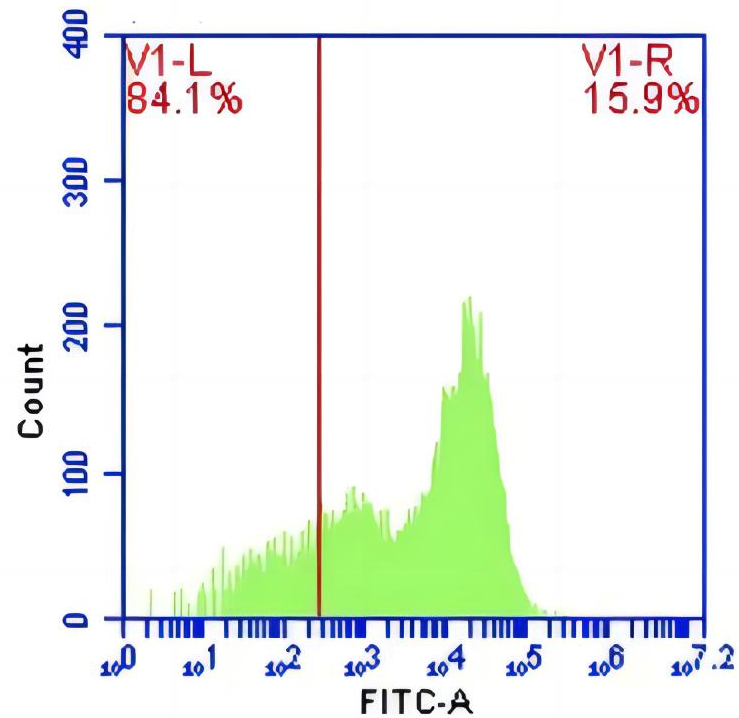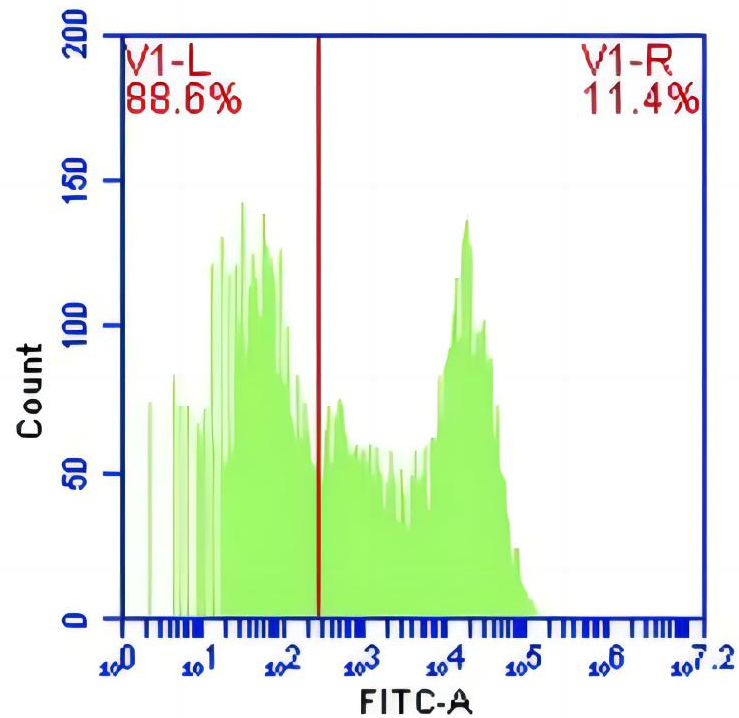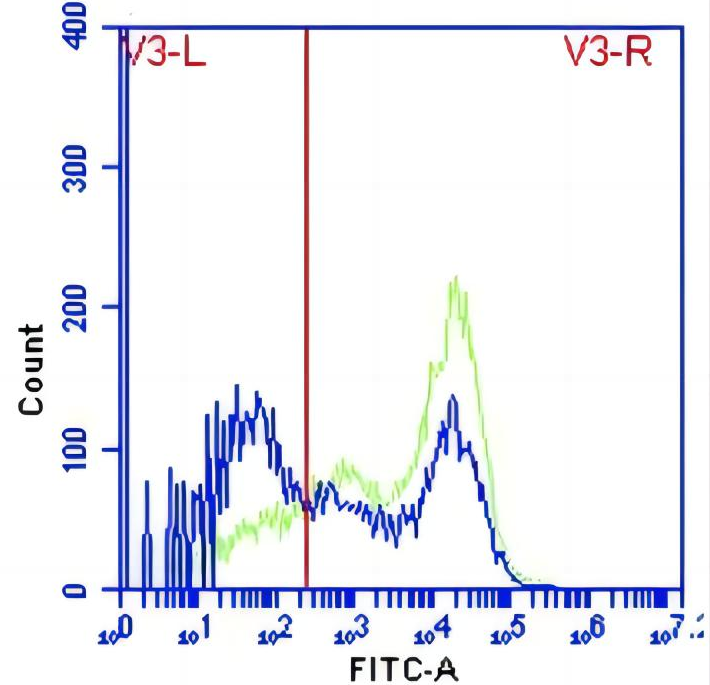

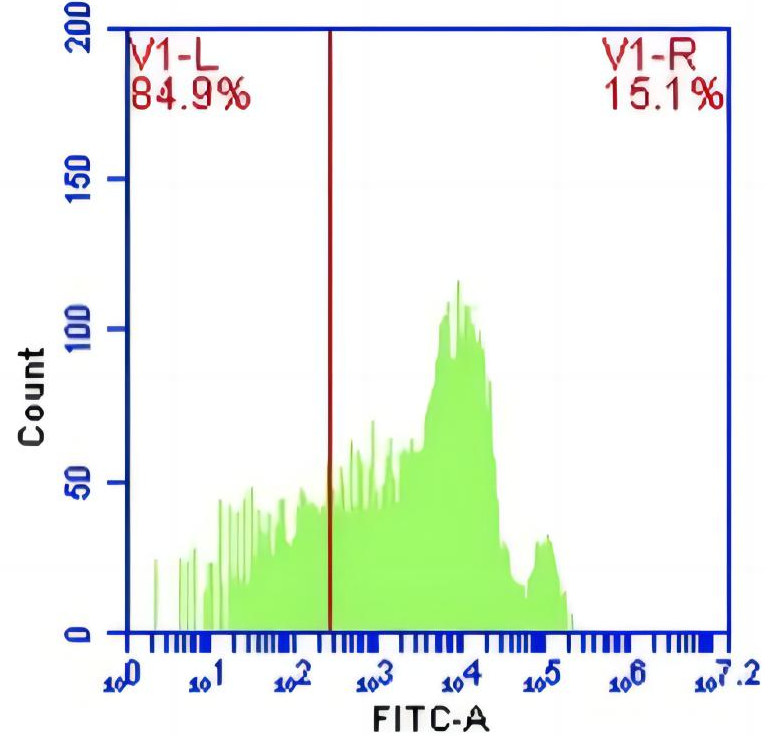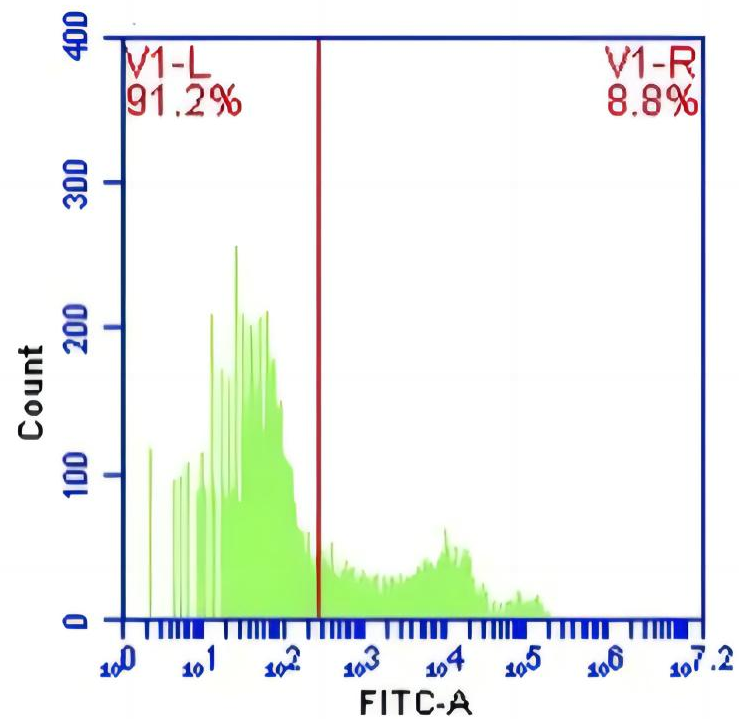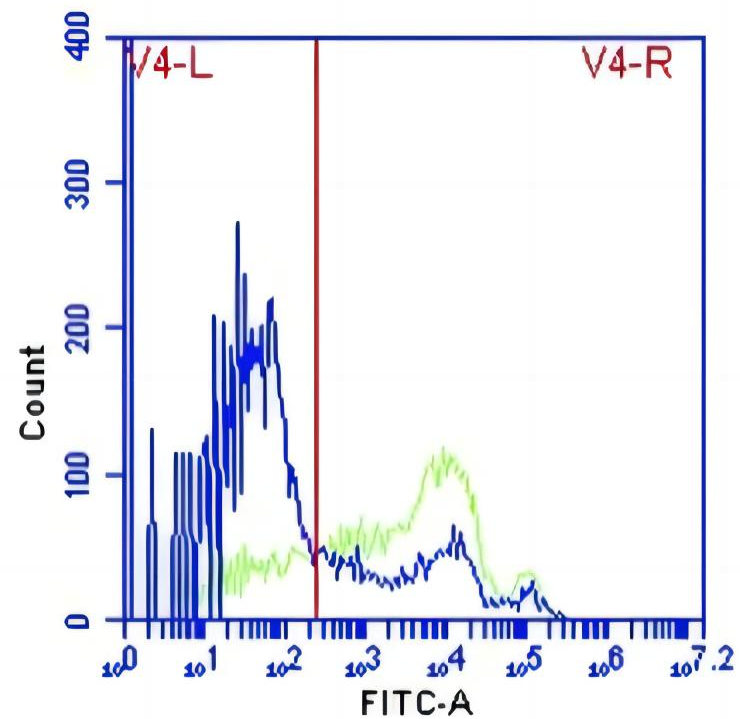

Supplement: Supplemental Information 2 [file peerj-13-18934-s002.zip › Picture supplement/Figure 9/Figure 9.pdf]

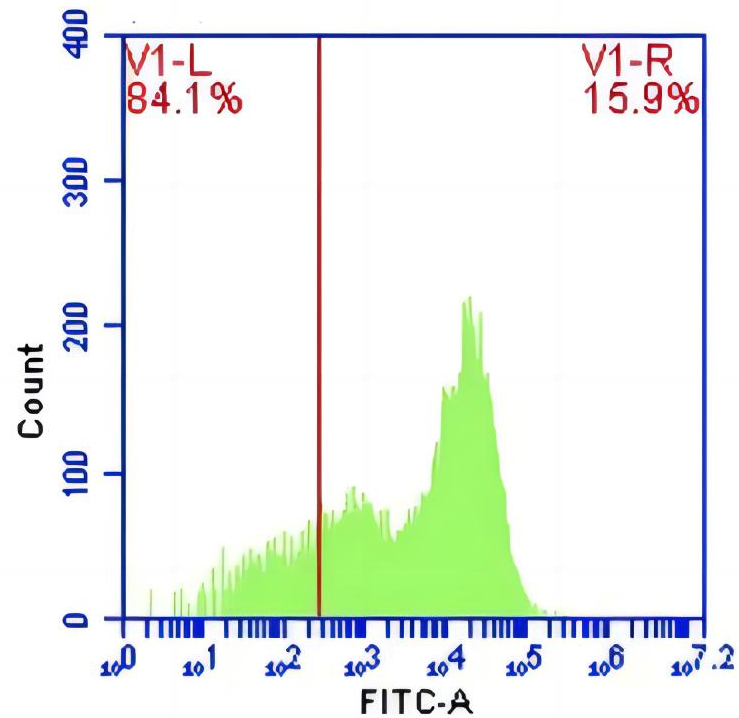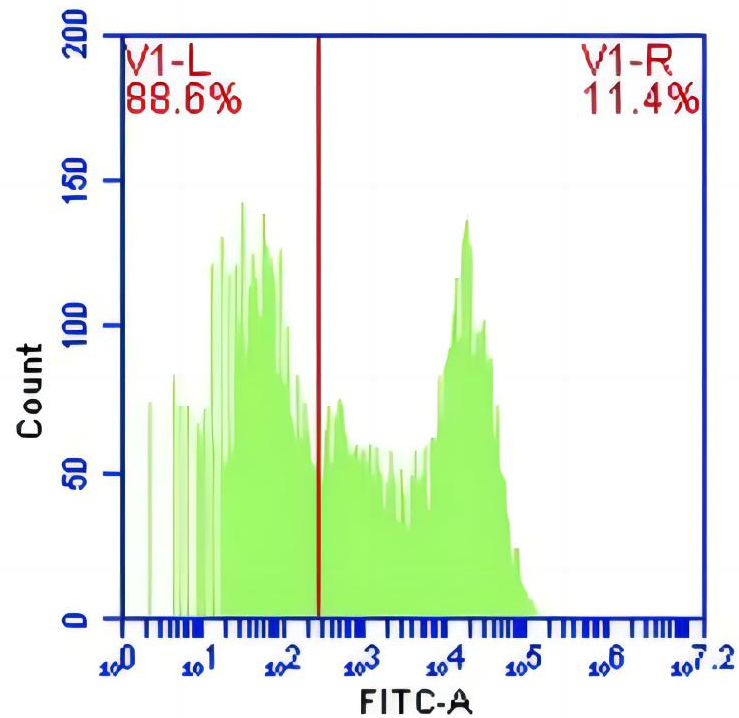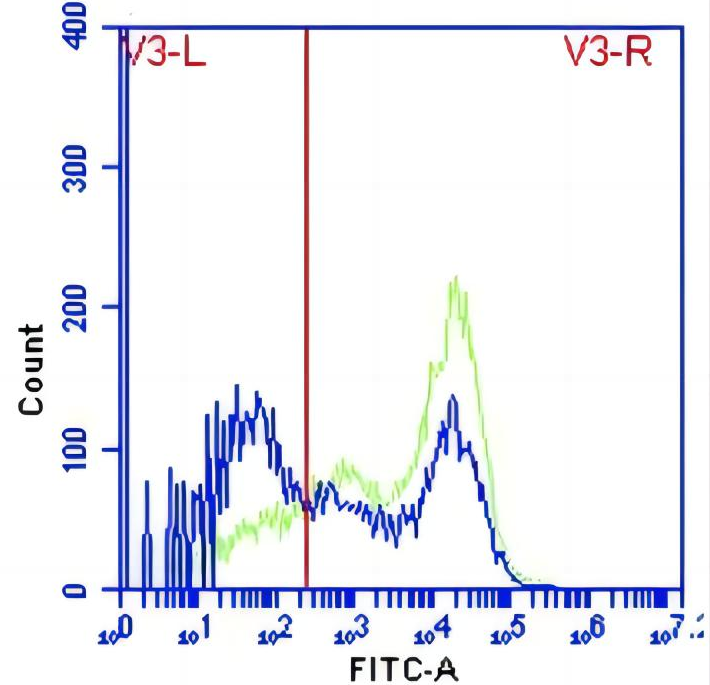

Supplement: Supplemental Information 2 [file peerj-13-18934-s002.zip › Picture supplement/Figure 9/Figure 9A.pdf]

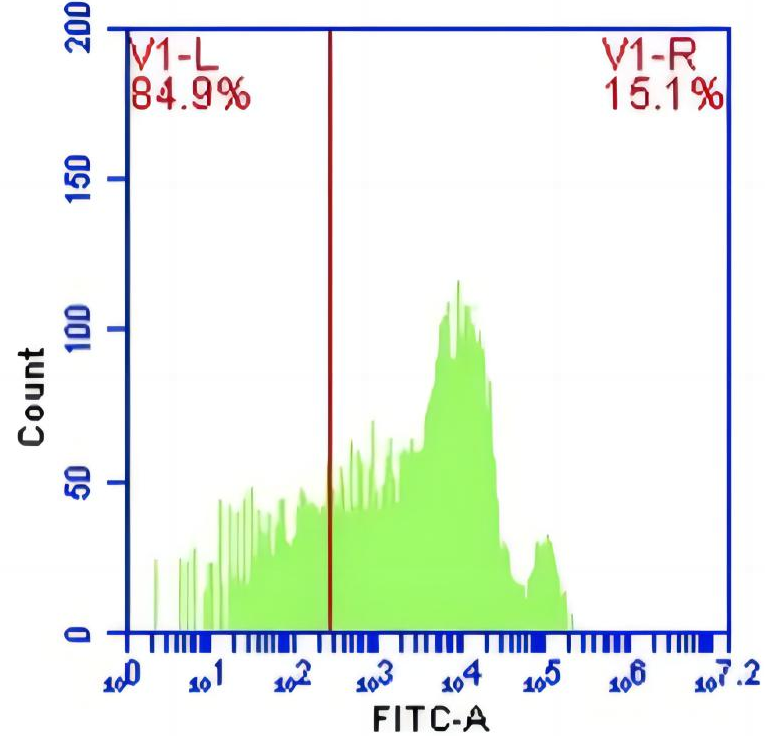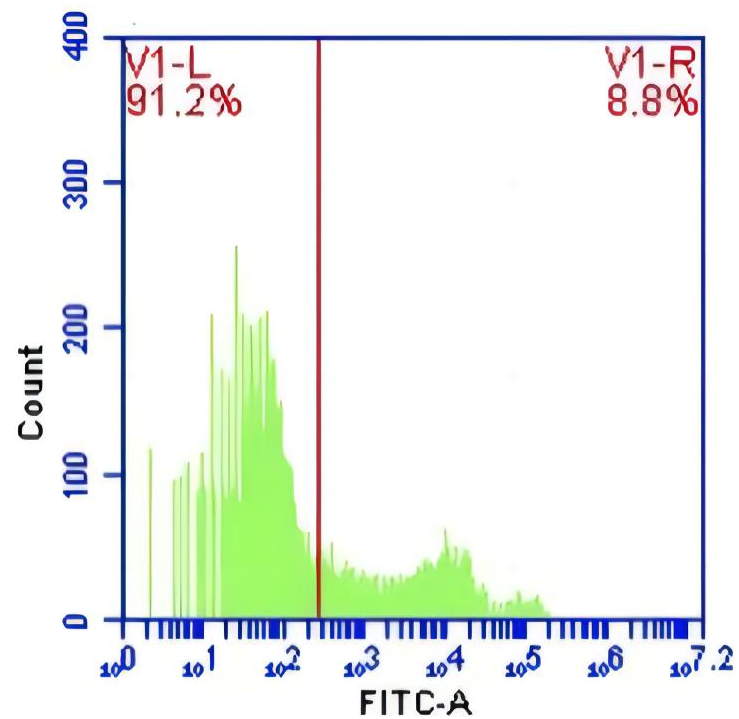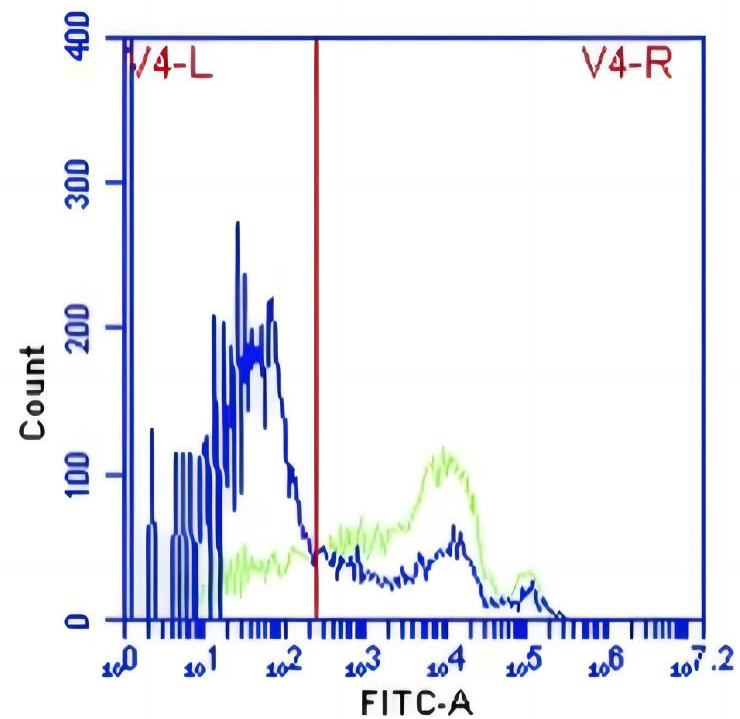

Supplement: Supplemental Information 2 [file peerj-13-18934-s002.zip › Picture supplement/Figure 9/Figure 9B.pdf]

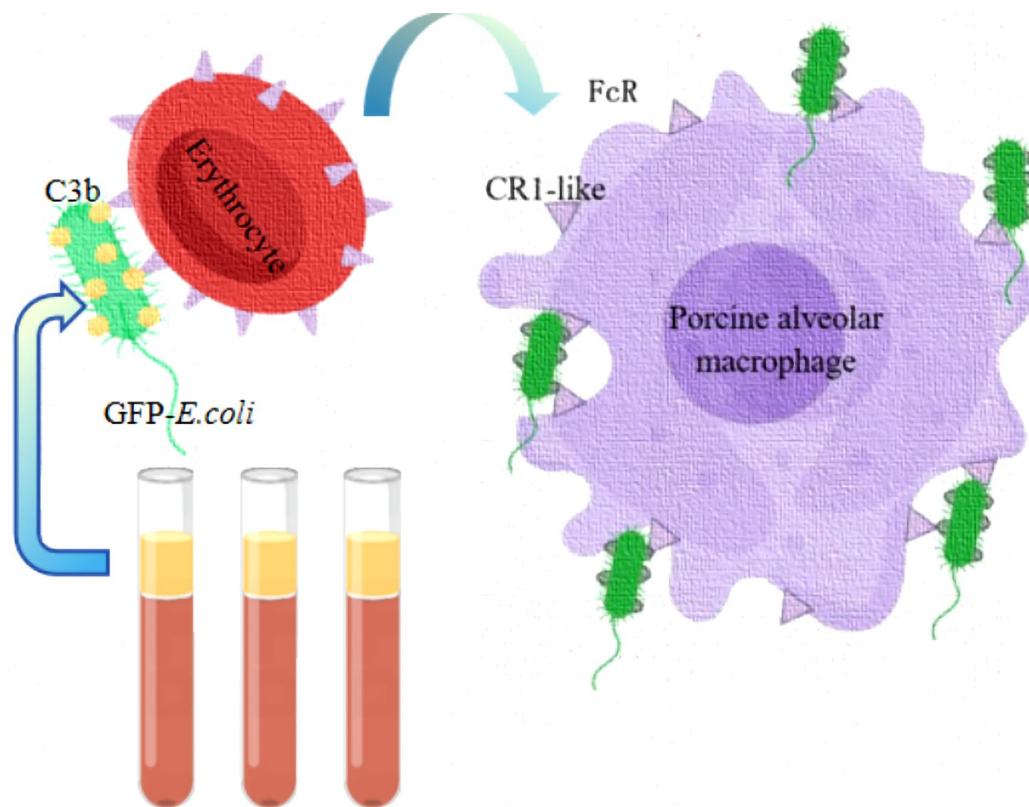

Supplement: Supplemental Information 3 [file peerj-13-18934-s003.pdf]
