# Supplementary material for: Assay of cardiopulmonary bypass system for porcine alveolar macrophages removing GFP-E. coli from erythrocyte surfaces
Source: PeerJ. 2025 Mar 4;13:e18934. doi: 10.7717/peerj.18934 (PMC11887565; doi:10.7717/peerj.18934)

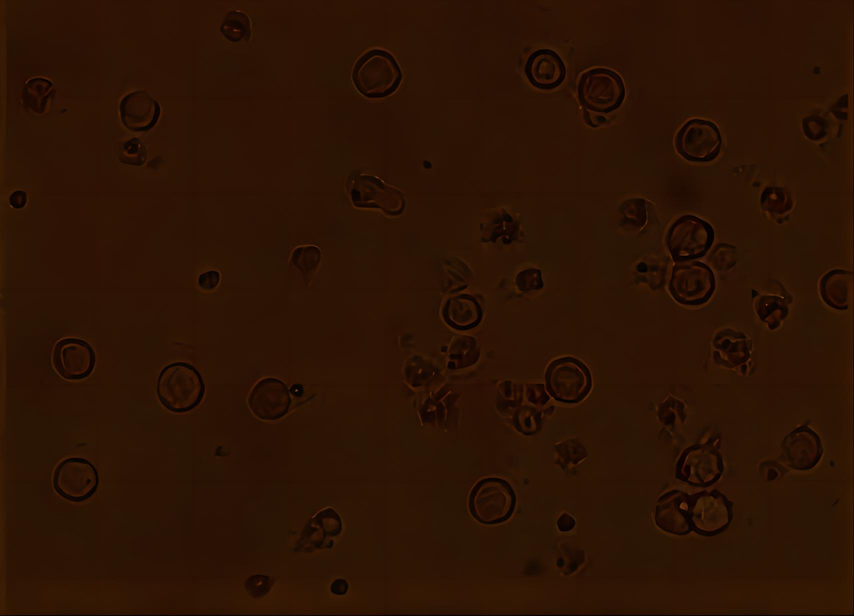

Supplement: Supplemental Information 2 [file Figure 1A-3.pdf › Picture supplement/Figure 1/Figure 1A-3.pdf]
